# Spatiotemporal regulation of DNA repair proteins between Golgi and nucleus maintains genome stability

**DOI:** 10.1083/jcb.202605024

**Published:** 2026-07-28

**Authors:** George Galea, Karolina Kuodyte, Muzamil Majid Khan, Peter J. Thul, Beate Neumann, Emma Lundberg, Rainer Pepperkok

**Affiliations:** 1 Cell Biology and Biophysics Unit, European Molecular Biology Laboratory, Heidelberg, Germany; 2 Collaboration for Joint PhD Degree Between European Molecular Biology Laboratory and Heidelberg University, Faculty of Biosciences, Heidelberg, Germany; 3 Translational Lung Research Center Heidelberg, German Center for Lung Research, Heidelberg, Germany; 4 Science for Life Laboratory, School of Engineering Sciences in Chemistry, Biotechnology and Health, KTH - Royal Institute of Technology, Stockholm, Sweden; 5 Advanced Light Microscopy Facility, European Molecular Biology Laboratory, Heidelberg, Germany; 6 Department of Bioengineering, Stanford University, Stanford, CA, USA; 7 Department of Pathology, Stanford University, Stanford, CA, USA

## Abstract

The Golgi complex serves as a critical hub for cellular homeostasis, yet its communication with the nucleus remains largely unexplored. By analyzing and siRNA-validating localization data from the Human Protein Atlas, we uncovered substantial proteome interconnectivity between the Golgi and nucleus, including an unexpected enrichment for DNA repair factors. We identify a cluster of DNA damage response (DDR) proteins occupying distinct sub-Golgi compartments that redistribute dynamically between the Golgi and nucleus in response to genotoxic stress, with the type of DNA lesion shaping the direction of redistribution. Focusing on the homologous recombination (HR) regulator RAD51C, we show that DNA damage triggers ataxia telangiectasia mutated (ATM)–dependent release of a giantin-tethered Golgi RAD51C pool, with subsequent importin-β–dependent nuclear import, where repair-associated foci form. Giantin depletion prematurely releases RAD51C, producing aberrant nuclear foci lacking key DDR markers, reducing ATM activation and HR efficiency, elevating genome instability, and accelerating proliferation. The Golgi thus acts as a spatiotemporal coordination node for DDR factors safeguarding genomic stability.

## Teaser

The Golgi is a well-established cytoplasmic hub, yet its coordination with the nucleus remains poorly understood. Systematic analysis of Golgi–nuclear proteins uncovers a DNA repair network, where RAD51C is anchored by giantin until DNA damage triggers ATM-dependent release and nuclear import, establishing the Golgi as a spatiotemporal regulator of nuclear DNA repair.

## Introduction

Eukaryotic cells have evolved a highly specialized and coordinated array of membrane-bounded organelles. This compartmentalization allows the segregation of biochemical reactions, ensuring that they are carried out with the highest specificity and efficiency. However, organelles do not function in isolation but rely on the continual exchange of lipids, proteins, and signaling cues to maintain cellular homeostasis. At the center of this cross-coordination of subcellular transport and signaling pathways lies the Golgi complex. It thus contributes well beyond its classical roles of membrane trafficking and posttranslational modification, also acting as a regulatory hub with numerous cellular processes intersecting at this organelle such as autophagy, mitosis, growth signaling, cytoskeletal organization, and energy status regulation (Wilson et al., 2011; Makhoul et al., 2018). Perturbations to the Golgi architecture and mutations of its constituents have been associated with a wide array of human diseases such as neurodegenerative disorders and cancer, among many others (Freeze and Ng 2011; Machamer 2015; Zappa et al., 2018; Liu et al., 2021). Although we have a clearer picture of the Golgi’s interactions and regulatory functions in the cytoplasmic domain, its communication with the nucleus remains largely unexplored.

Emerging themes have started to illustrate the relationship between the Golgi and nuclear compartment, with multi-localization proteins playing a pivotal role. For example, in cholesterol homeostasis and endoplasmic reticulum (ER) stress response, sensing-signaling proteins SREBP and ATF6, respectively, get proteolytically cleaved at the Golgi complex to regulate gene expression through the release of a transcriptionally active amino terminus that makes its way to the nucleus (Haze et al., 1999; Brown et al., 2018). Several studies have also hinted at a link between cytoplasmic organelles, genomic stability, and, in turn, cancer, with the Golgi complex emerging as a central theme (Petrosyan 2015; Kulkarni-Gosavi et al., 2019; Zhang 2021). At a structural level, the Golgi undergoes dramatic morphological changes following the induction of DNA lesions, from ribbon-like perinuclear stack to dispersed fragments (Farber-Katz et al., 2014). This response requires the phosphorylation of the Golgi-resident oncoprotein, Golgi phosphoprotein 3, by DNA-dependent protein kinase (DNA-PK), a DNA damage response (DDR) regulator (Farber-Katz et al., 2014). Golgi morphology alterations are also a common feature across a wide variety of cancer types and are often reflected in changes in the distribution of Golgi-resident proteins (Petrosyan, 2015; Zhang, 2021). Rearrangement of Golgi glycosyltransferases distribution is a recurrent phenomenon in cancer cells, resulting in defective glycosylation, a process thought to promote cancer development (Petrosyan 2015; Bui et al., 2021; Zhang 2021). These studies hint at a clear link between Golgi and nucleus as communicating organelles; however, a systematic approach that could pin down the signaling pathways involved in this communication has been missing.

To this end, here, we utilize localization data and antibody resources from the Human Protein Atlas (HPA) project (Thul et al., 2017) to explore a class of multi-localization proteins as a systematic strategy to identify key signaling pathways that function between the Golgi and nuclear compartment. We validate candidate Golgi–nuclear localizations by siRNA-mediated knockdown revealing a network of DNA repair proteins localized at the Golgi complex. We systematically analyze their subcellular localization within the Golgi, as well as their redistribution patterns in response to different types of DNA damage. Building on these observations, we propose a spatiotemporal regulatory pathway for DDR control between the Golgi complex and nucleus that gates the timed availability of homologous recombination (HR) regulators. Using RAD51C as a representative HR factor, we find that a Golgi-resident pool is released to the nucleus upon DNA damage, where it forms repair-associated foci, and that the Golgi scaffold giantin (GOLGB1) is required for RAD51C localization to the Golgi. Disrupting this regulation through giantin depletion leads to premature nuclear accumulation of RAD51C, reduced ataxia telangiectasia mutated (ATM) activation and HR efficiency, increased genomic instability, and accelerated cell proliferation. Together, these findings highlight the importance of spatial regulation of DDR proteins in maintaining genomic integrity and cellular homeostasis and suggest a broader role for the Golgi as a regulatory platform in DNA repair.

## Results

### Antibody-based systematic analysis and siRNA-mediated validation identifies a network of DDR proteins at the Golgi complex

**Figure 1.**
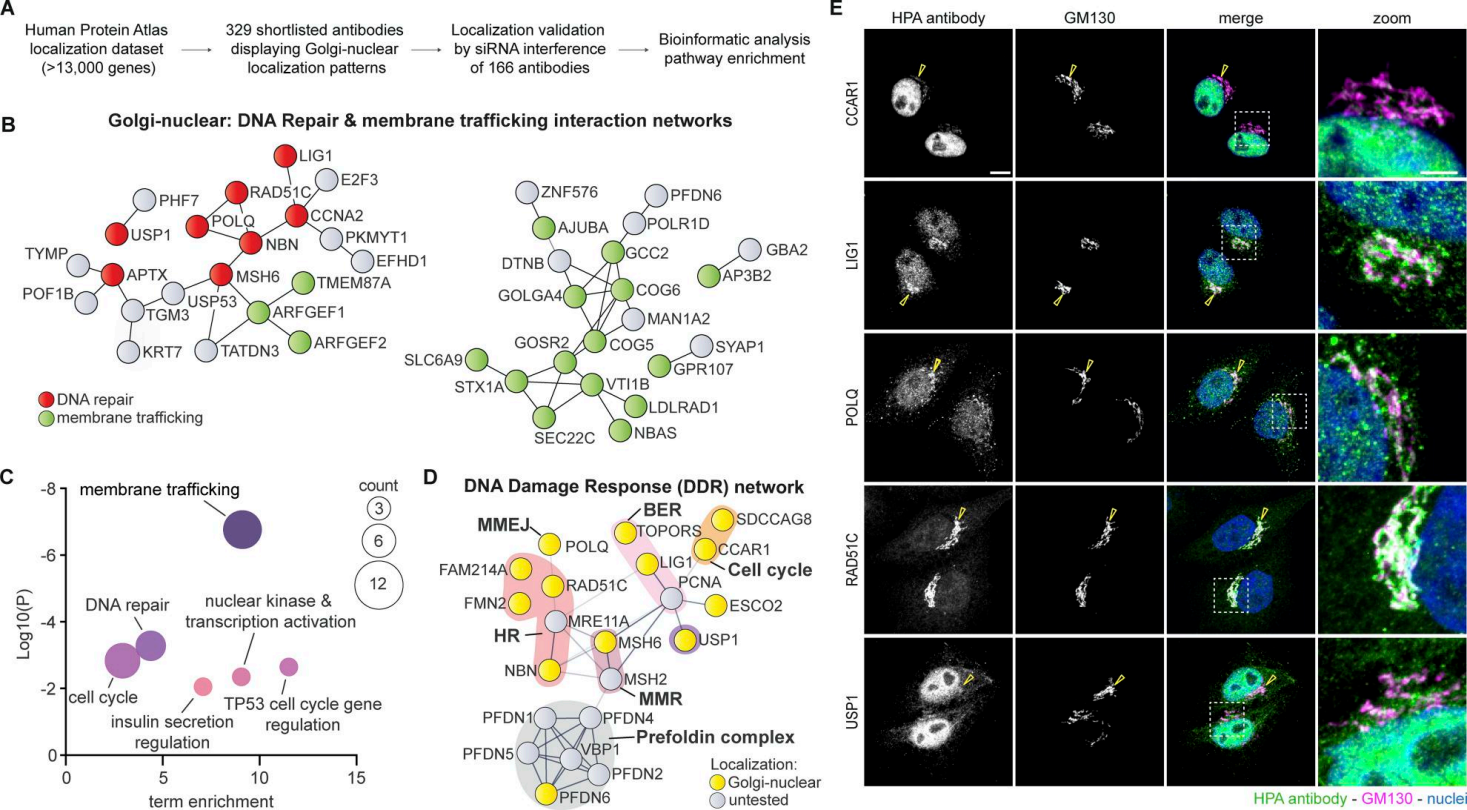
**Antibody-based analysis identifies a network of DDR proteins at the Golgi complex. (A)** Schematic diagram of the screening process for the shortlisting, validation, and characterization of Golgi–nuclear localization proteins, as a strategy to identify linking pathways. **(B)** Experimentally based protein–protein interaction network displaying the DNA repair and membrane trafficking Golgi–nuclear localization proteins validated in this study, categorized by functional pathways. Annotations were assigned based on Gene Ontology terms and literature curation. **(C)** Pathway enrichment analysis of the Golgi–nuclear localization proteins identified in this study. Annotations were assigned based on Gene Ontology terms. **(D)** Experimentally based STRING protein–protein interaction network showing the DDR proteins identified to localize to both the Golgi complex and nucleus. DDR pathway annotations were assigned based on Gene Ontology terms and literature curation. Yellow nodes indicate double-localizing proteins, and grey nodes are filler untested proteins. **(E)** Representative images of HeLa-K cells stained with HPA antibodies against Golgi–nuclear DDR proteins (green), and the Golgi marker GM130 (red). DNA was stained with Hoechst 33342 (blue). Yellow arrowheads denote the Golgi complex. Scale bars, 10 μm (overview), 5 μm (zoomed images).

To systematically explore candidates that might link Golgi to nuclear function or vice versa, we shortlisted 329 proteins annotated by the HPA project (Thul et al., 2017) to localize at both Golgi-like membranes and the nucleus (Fig. 1 A). To ensure the specificity of both localization data and antibody binding, we tested the corresponding HPA antibody for each candidate using an siRNA-mediated knockdown pipeline (Stadler et al., 2012). This approach was specifically designed to exclude false localization arising from antibody cross-reactivity. Given the inherent variability in knockdown efficiency and the fact that screening conditions (fixation, permeabilization, antibody concentration, and blocking) were kept uniform across all candidates rather than optimized for each individual antibody, validation was applied if the 25% reduction threshold was met in at least one of the two experimental replicates in both compartments. Candidates taken forward for mechanistic study were subject to more stringent orthogonal validation.

**Figure S1.**
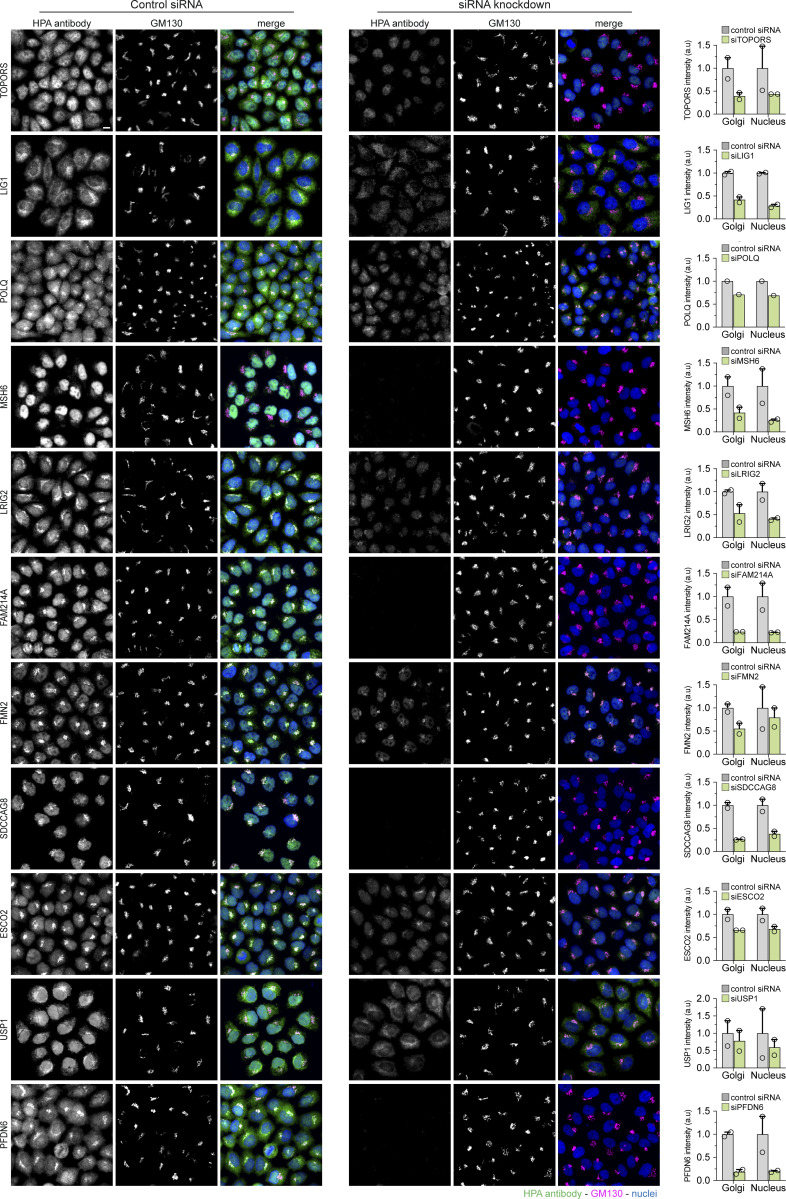
**Validation of DDR protein antibody specificity and localization, related to Fig. 1.** Representative images of HeLa-K cells stained with antibodies against DDR proteins and the Golgi marker, GM130. Cells were treated with a control or an siRNA against the protein of interest to validate the specificity of the antibody. The changes in intensity measurements in the Golgi and nuclear compartment were quantified (*n* = 2 for all proteins except for POLQ, which had *n* = 1, biologically independent experiments with at least 200 cells analyzed for each treatment). Scale bar, 10 μm. Continued on Fig. S2 A.

**Figure S2.**
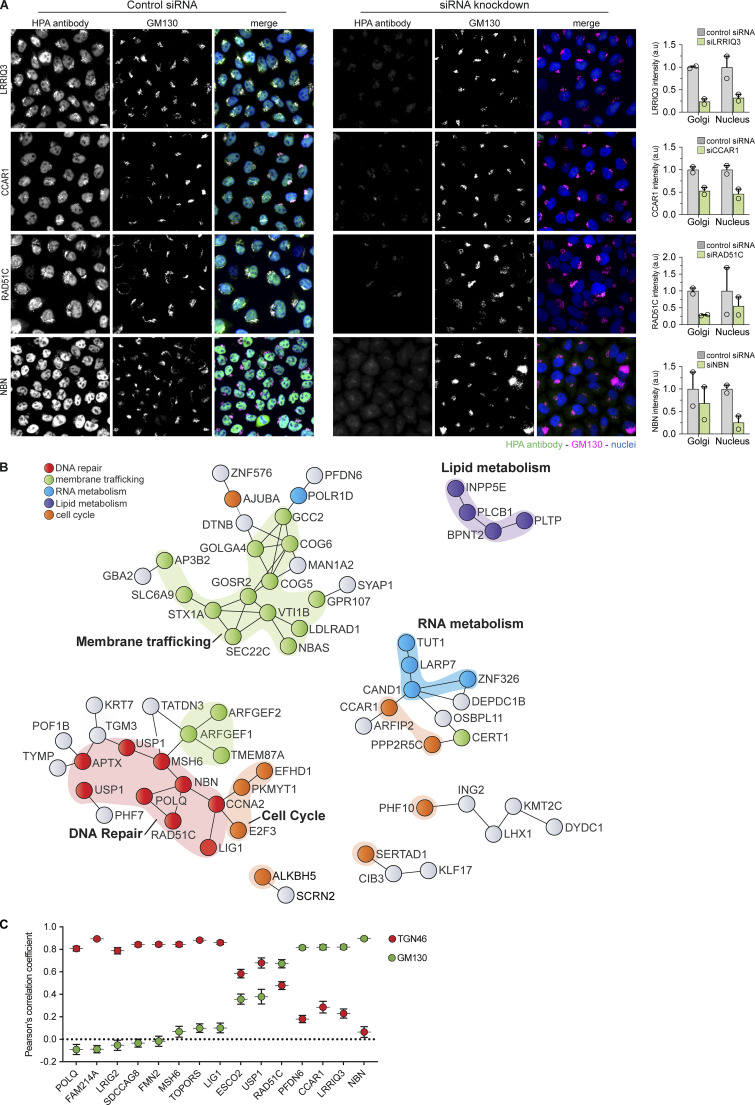
**Validation of DDR protein localization and functional network analysis, related to Fig. 1. (A)** Representative images of HeLa-K cells stained with antibodies against DDR proteins and the Golgi marker, GM130. Cells were treated with a control or an siRNA against the protein of interest to validate the specificity of the antibody. The changes in intensity measurements in the Golgi and nuclear compartment were quantified. (*n* = 2, biologically independent experiments with at least 200 cells analyzed for each treatment.) Scale bar, 10 μm. **(B)** Experimentally based protein–protein interaction networks displaying the Golgi–nuclear localization proteins validated in this study. Proteins are categorized by functional pathways annotated using gene ontology terms and literature curation (Table S1). **(C)** Golgi-cisternal localization analysis of Golgi–nuclear DDR proteins. HeLa-K cells were treated with nocodazole (33 μM, 3 h), fixed, and stained with antibodies against DDR proteins. Quantification of PCC between *cis-*Golgi and *trans-*Golgi markers and DDR proteins; N ≥ 4 cells; *n* ≥ 36 mini-stacks.

Using this strategy, we confirmed the dual localization for 163 proteins (166 HPA antibodies) (Fig. 1 A and Table S1). To further validate the identified candidates and assess the biological significance of the dataset as a whole, we performed bioinformatic network analysis of the dual-localizing proteins, designed to determine whether entire functional pathways, rather than isolated proteins are represented at the Golgi and nuclear compartment. This analysis revealed a number of functional protein networks (Fig. 1 B), primarily composed of two major pathways: membrane trafficking and, surprisingly, DDR (Fig. 1, B and C; Fig. S1; and Fig. S2 A). The siRNA-mediated antibody validation data for the DDR candidates are shown in Fig. S1 and Fig. S2 A demonstrating a reduction in both Golgi and nuclear signal upon knockdown across proteins from multiple repair pathways. The full experimentally derived protein–protein interaction network of the validated dual-localizing proteins is shown in Fig. S2 B. Additionally, we identified enrichment for cell cycle regulation, RNA metabolism, and lipid metabolism regulators (Table S1 and Fig. S2 B). Within the membrane trafficking enriched cluster, we identified several essential components of intra-Golgi trafficking machinery, including golgins, SNAREs, COG tethering complex, and RAB GTPase exchange factors (Fig. 1 B).

An enriched cluster centered on the DDR emerged through the combination of DNA repair proteins and an array of cell cycle regulators (Fig. 1 D, Fig. S1, and Fig. S2). Within the DDR cluster, we identified proteins spanning the core DNA repair pathways, HR, mismatch repair (MMR), microhomology-mediated end joining (MMEJ), and base excision DNA repair (BER), as well as other integral regulators of DDR, including ubiquitination, cell cycle, and signaling factors (Fig. 1, D and E).

### Golgi-cisternal localization analysis of dual-localizing DDR proteins identifies specific distribution patterns that correlate with their function

**Figure 2.**
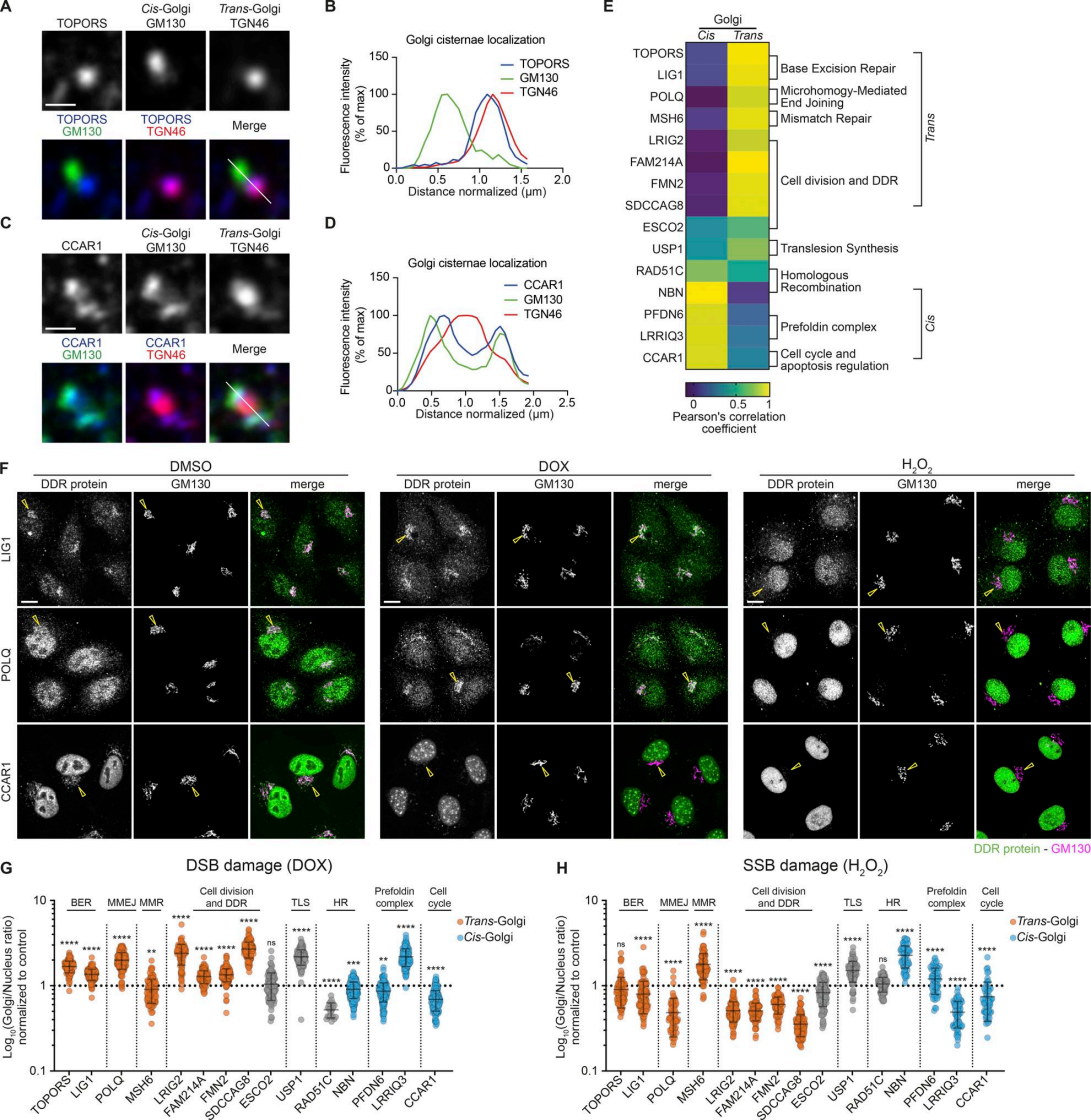
**Systematic analysis of Golgi–nuclear DDR proteins localization. (A–D)** Golgi-cisternal localization analysis of Golgi–nuclear DDR proteins. HeLa-K cells were treated with nocodazole (33 μM, 3 h), fixed, and stained with antibodies against the *cis*-Golgi marker GM130, the *trans*-Golgi marker TGN46, and DDR proteins. An enlarged view of a single isolated mini-stack stained for TOPORS (A) and CCAR1 (C) are shown; white lines across the stack indicate the regions used for line-plot analyses shown in B and D. Scale bars, 1 μm. **(E)** Quantification of PCC between *cis*-Golgi and *trans*-Golgi markers and DDR proteins; *n* ≥ 36 mini-stacks. **(F)** Representative images of localization changes in dual-localizing DDR proteins upon DNA damage. HeLa-K cells were treated with DOX (40 μM, 3 h) or H_2_O_2_ (50 μM for 20 min, followed with 15-min recovery), fixed, and stained with antibodies against DDR proteins (LIG1, POLQ, and CCAR1) and the Golgi marker, GM130. Yellow arrowheads denote the Golgi membranes. Scale bars, 10 μm. **(G and H)** Quantification of DDR protein Golgi–nuclear distribution ratio after treatment with (G) DOX or (H) H_2_O_2_. Data represent the mean ± standard error of the mean (SEM) (*n* = 3 biologically independent experiments with at least 200 cells analyzed for each protein in control and treatment conditions). Proteins are classified according to their Golgi localization patterns from E: orange, co-localizing with the *trans-*Golgi marker TGN46; blue co-localizing with the *cis-*Golgi marker GM130; grey, no marked correlation with either markers. Statistical significance was determined using a two-tailed unpaired Student’s *t* test; ns, **P < 0.01, ***P < 0.001, and ****P < 0.0001, compared with untreated control.

To start addressing the functional meaning of DDR proteins localization at the Golgi and provide an additional layer of localization validation, we shortlisted 15 candidates based on bioinformatic annotations and their literature-described roles in DNA repair and maintenance pathways, ensuring representation of the major repair pathways identified in our screen. We first analyzed their subcellular distribution within the organelle. The Golgi apparatus consists of *cis*-, *medial*, and *trans*-Golgi cisternae, extending into a tubular *trans*-Golgi network (TGN), with resident proteins strategically distributed along the Golgi stack to serve their function (Kulkarni-Gosavi et al., 2019). Since the ribbon-like Golgi structures make subdomain mapping difficult, we used nocodazole treatment to disperse the ribbon into isolated mini-stacks, a standard approach that enables high-resolution analysis of cisternal distribution (Dejgaard et al., 2007). We then performed confocal microscopy with co-staining for the *cis*-Golgi marker GM130 and the *trans*-Golgi marker TGN46. Fluorescence intensity line profiles were acquired across individual mini-stacks, and the relative localization of each DDR protein was quantified by calculating Pearson’s correlation coefficients (PCCs) with the *cis*- and *trans*-markers (Dejgaard et al., 2007) (Fig. 2, A–E, Fig. S2 C, and Table S2).

**Figure 3.**
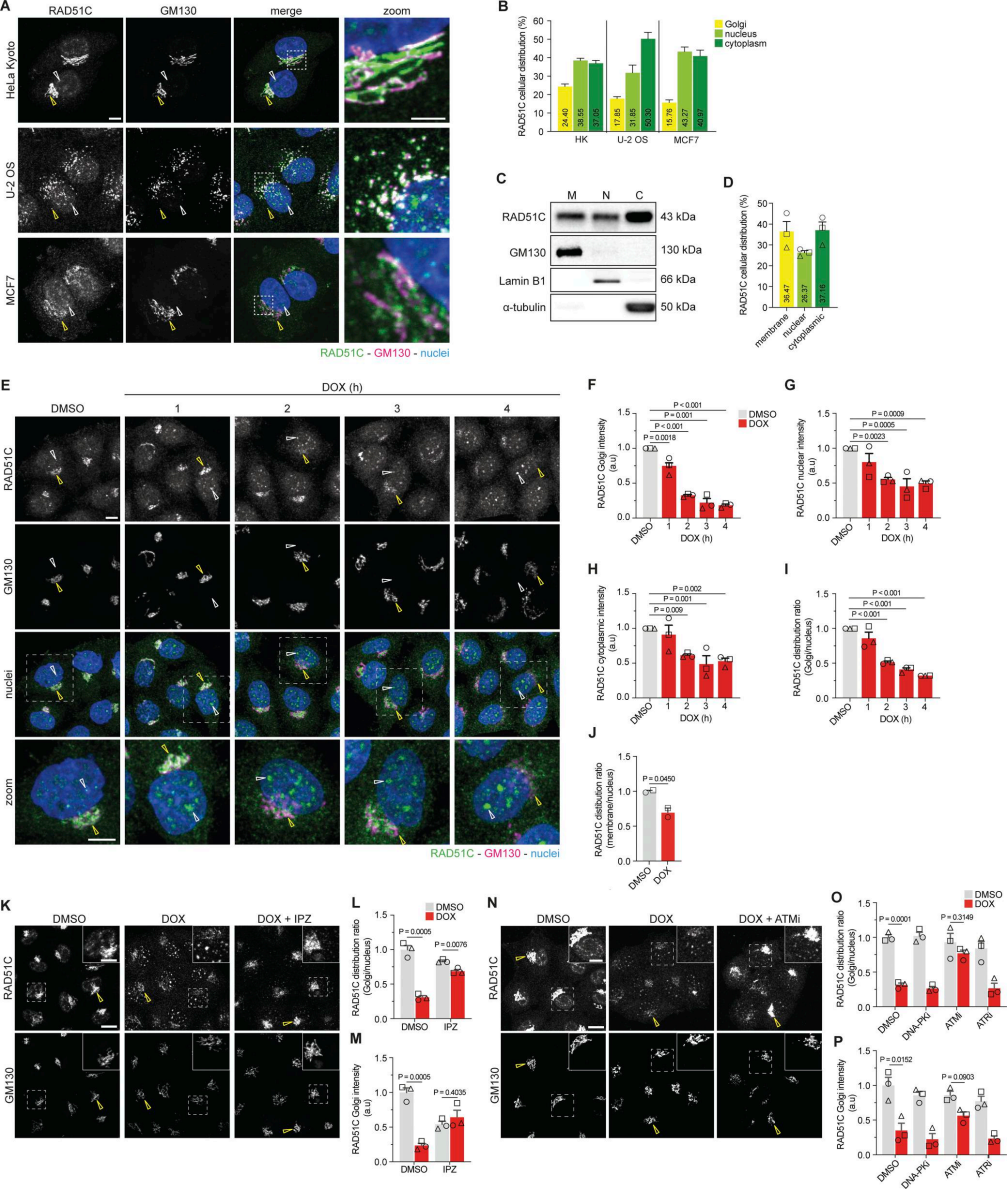
**Redistribution of RAD51C Golgi fraction is required for the formation of RAD51C nuclear foci and is dependent on the kinase ATM.** Immunofluorescence in this figure was performed using RAD51C antibody ab72063; biochemical fractionation in C used RAD51C antibody ab95069. Antibody specificity is shown in Fig. S6. **(A)** Representative images of HeLa-K, U-2 OS, and MCF7 cells stained with antibodies against RAD51C (green) and GM130 (red). DNA stained with Hoechst 33342 (blue). Scale bars, 10 μm (overview), 5 μm (zoomed images). **(B)** Quantification of RAD51C distribution across the Golgi, nucleus, and cytoplasm in the cell lines shown in A measured from immunofluorescence images. **(C)** Western blot analysis showing the subcellular membrane (M), nuclear (N), and cytoplasmic (C) fractions of RAD51C. Compartment markers: GM130 (Golgi membranes), Lamin B1 (nuclear), and α-tubulin for the (cytoplasmic). **(D)** Quantification of western blot analysis (C), showing the distribution of RAD51C across 3 cell fractions: membrane, nuclear, and cytoplasmic; *n* = 3 biologically independent experiments; data represent the mean ± SEM. **(E)** Representative images of HeLa-K cells treated with DOX (40 μM), for increasing lengths of time. Yellow arrowheads denote the Golgi membrane; white arrowheads denote nuclear foci. Scale bars, 10 μm (overview), 10 μm (zoomed images). **(F–I)** Quantification of RAD51C sum intensity at the Golgi (F), in the nucleus (G), and in the cytoplasm (H), and RAD51C Golgi–nuclear distribution ratio (I), following DOX treatment. Data represent the mean ± SEM (*n* = 3 biologically independent experiments with a total of 2,343 cells analyzed). Statistical significance determined by one-way ANOVA with Dunnett’s post hoc test vs. DMSO control. **(J)** Quantification of RAD51C membrane-nuclear distribution ratio after DOX treatment, calculated from subcellular fractions (Fig. S6 D). **(K)** Representative images of HeLa-K cells treated with DMSO or IPZ (20 μM) prior to a 3-h DOX treatment. Yellow arrowheads denote the Golgi membranes; white denote nuclear foci. Scale bars, 10 μm (overview), 5 μm (zoomed images). **(L and M)** Quantification of RAD51C Golgi–nuclear distribution ratio (L) and relative RAD51C Golgi intensity (M) following IPZ and DOX treatment. Data represent the mean ± SEM. (*n* = 3 biologically independent experiments; 445 total cells analyzed). Statistical significance determined by two-tailed unpaired Student’s *t* test. **(N)** Representative images of HeLa-K cells treated with DMSO, ATM phosphorylation inhibitor (KU55933; 30 μM), ATR inhibitor (VE-821; 10 μM), or DNA-PK inhibitor (NU7441; 10 μM) prior to a 3-h DOX treatment. Yellow arrowheads denote the Golgi membrane. Scale bars, 10 μm (overview), 5 μm (zoomed images). **(O and P)** Quantification of RAD51C Golgi–nuclear distribution ratio (O) and relative RAD51C Golgi intensity (P) following kinase inhibitor and DOX treatment. Data represent the mean ± SEM (*n* = 3 biologically independent experiments; 1,679 total cells analyzed). Statistical significance determined by two-tailed unpaired Student’s *t* test comparing DMSO and DOX conditions within each inhibitor treatment. Source data are available for this figure: SourceData F3.

This analysis resolved three major localization patterns throughout the Golgi cisternae: (1) a subset preferentially correlating with the *trans*-Golgi marker, TGN46 (Fig. 2, A, B, and E), (2) proteins correlating with the *cis*-Golgi marker, GM130 (Fig. 2, C–E), and (3) proteins without a strong preference for either marker. Although the number of proteins examined was limited, emerging trends suggest pathway-related distribution (Fig. 2 E). For instance, BER and MMEJ proteins TOPORS, LIG1, and POLQ exhibited co-localization with the *trans-*Golgi marker, TGN46, while HR factors nibrin (NBN) and RAD51C had a high correlation with the *cis-*Golgi marker, GM130 (Fig. 2 E and Fig. S2 C).

These observations suggested that DDR proteins may not only localize to the Golgi but also occupy distinct cisternal subdomains, raising the possibility that sub-Golgi positioning could influence how different repair pathways respond to DNA damage. We therefore examined whether these proteins dynamically redistribute between the Golgi and the nucleus in response to genotoxic stress.

### Dual-localizing DDR proteins dynamically redistribute between the Golgi and nucleus in response to specific types of DNA lesions

**Figure S3.**
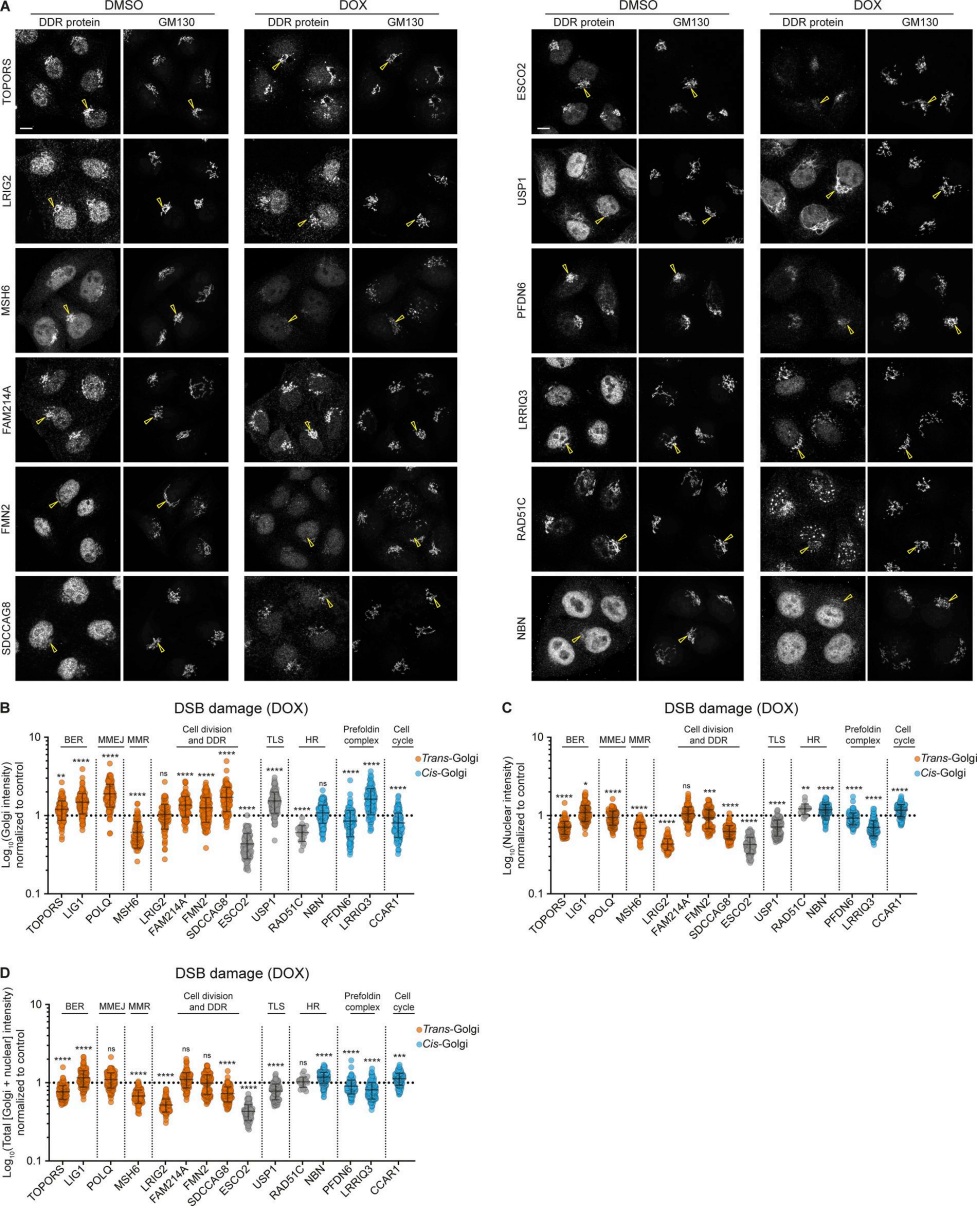
**DOX-induced redistribution, related to Fig. 2. (A)** Representative images of HeLa-K cells stained with antibodies against DDR proteins and the Golgi marker, GM130. Cells were treated with DOX (40 μM, 3 h), prior to fixation. Yellow arrowheads denote Golgi membranes. Scale bars, 10 μm. **(B–D)** Quantification of DDR protein intensity changes upon DOX treatment. Relative intensity of DDR proteins (B) at the Golgi and (C) in the nucleus and (D) total Golgi and nuclear signal normalized to untreated control. Data represent the mean ± SEM (*n* = 3 biologically independent experiments with at least 200 cells analyzed for each protein in control and treatment conditions). The proteins are classified according to their Golgi localization patterns (Fig. 2 E). Proteins shown in orange co-localize with the *trans-*Golgi marker TGN46, while those in blue co-localize with the *cis-*Golgi marker GM130. Proteins whose localization showed no clear preference for either marker are shown in grey. Statistical significance was determined using a two-tailed unpaired Student’s *t* test; ns, *P < 0.05, **P < 0.01, ***P < 0.001, and ****P < 0.0001, compared with untreated control.

**Figure S4.**
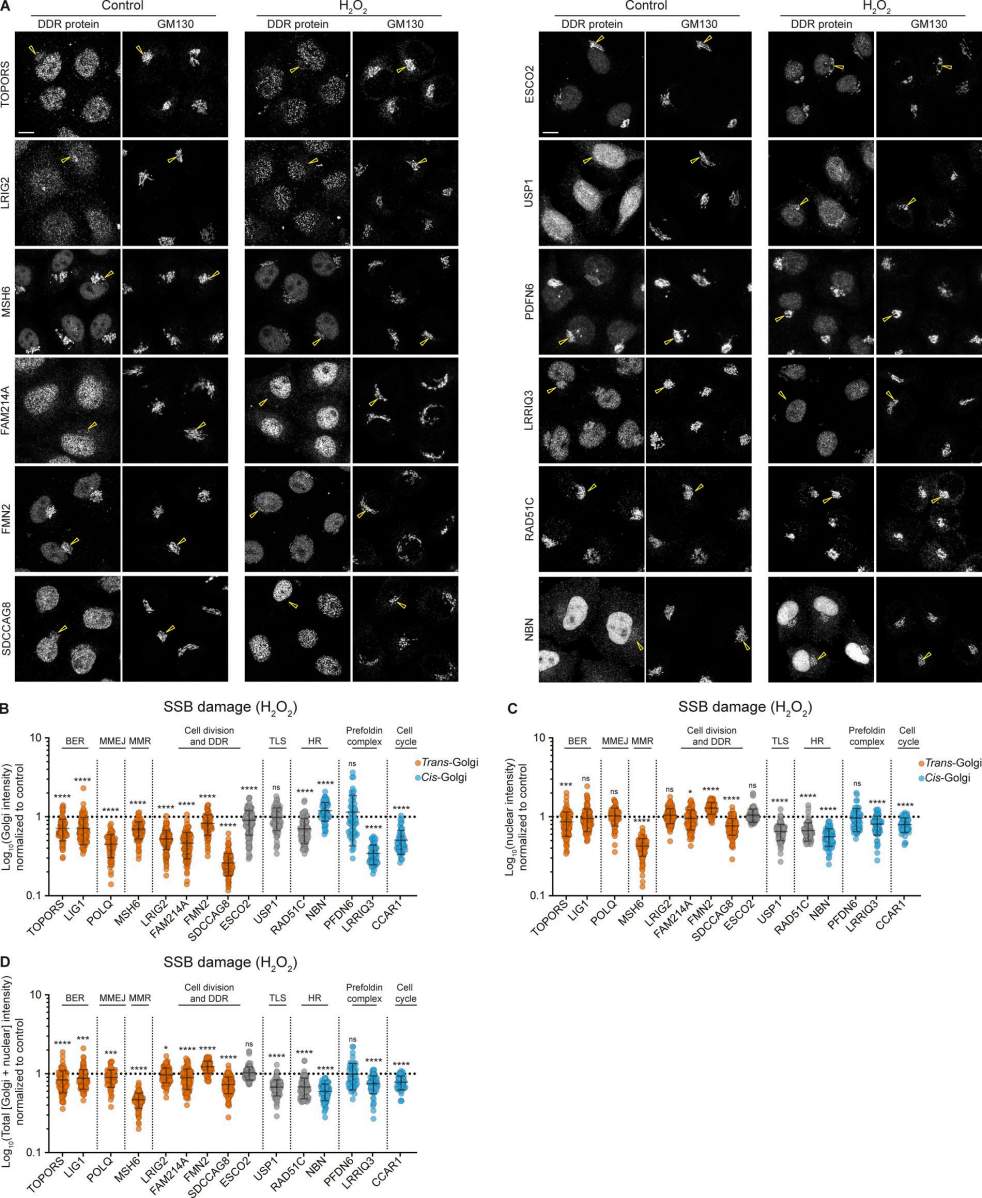
**H**_**2**_ **O**_**2**_ **-induced redistribution of DDR proteins, related to Fig. 2. (A)** Representative images of HeLa-K cells were stained with antibodies against the indicated DDR proteins and the Golgi marker, GM130 following treatment with H_2_O_2_ (50 μM, for 20 min followed by 15-min recovery). Yellow arrowheads denote the Golgi membranes. Scale bars, 10 μm. **(B–D)** Quantification of DDR protein intensity changes upon H_2_O_2_ treatment. Relative intensity of DDR proteins (B) at the Golgi and (C) in the nucleus and (D) total Golgi and nuclear signal normalized to untreated control. Data represent the mean ± SEM (*n* = 3 biologically independent experiments with at least 200 cells analyzed for each protein in control and treatment conditions). The proteins are classified according to their Golgi localization patterns (Fig. 2 E). Proteins shown in orange co-localize with the *trans-*Golgi marker TGN46, while those in blue co-localize with the *cis-*Golgi marker GM130. Proteins whose localization showed no clear preference for either marker are shown in grey. Statistical significance was determined using a two-tailed unpaired Student’s *t* test; ns, *P < 0.05, ***P < 0.001, and ****P < 0.0001, compared with untreated control.

**Figure S5.**
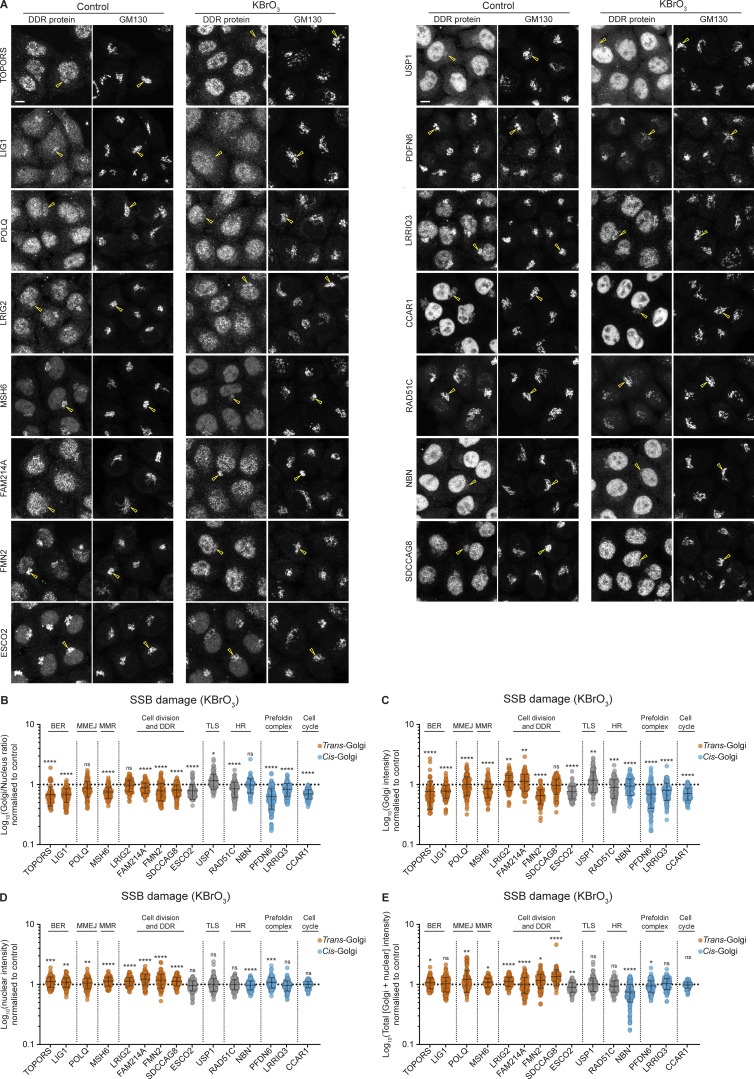
**KBrO**_**3**_ **-induced redistribution of DDR proteins, related to Fig. 2. (A)** Representative images of HeLa-K cells were stained with antibodies against DDR proteins and the Golgi marker, GM130. HeLa-K cells were treated with KBrO_3_ (5 mM, 3 h), fixed, and stained with antibodies against DDR proteins and the Golgi marker, GM130; yellow arrowheads denote the Golgi membranes. Scale bars, 10 μm. **(B)** A ratio of DDR protein Golgi–nuclear distribution after treatment with KBrO_3_. **(C–E)** Quantification of DDR protein intensity changes upon KBrO_3_ treatment. Relative intensity of DDR proteins (C) at the Golgi and (D) in the nucleus and (E) total Golgi and nuclear signal normalized to untreated control. Data represent the mean ± SEM (*n* = 3 biologically independent experiments with at least 200 cells analyzed for each protein in control and treatment conditions). The proteins are classified according to their Golgi localization patterns (Fig. 2 E). Proteins shown in orange co-localize with the *trans-*Golgi marker TGN46, while those in blue co-localize with the *cis-*Golgi marker GM130. Proteins whose localization showed no clear preference for either marker are shown in grey. Statistical significance was determined using a two-tailed unpaired Student’s *t* test; ns, *P < 0.05, **P < 0.01, ***P < 0.001, and ****P < 0.0001, compared with untreated control.

We next examined whether DDR proteins with dual Golgi–nuclear localization respond dynamically to DNA damage. To this end, we compared two classes of DNA damage: double-strand DNA breaks (DSBs) induced by doxorubicin (DOX), which acts primarily through topoisomerase II poisoning to generate DSBs (Thorn et al., 2011) and oxidative DNA lesions induced by hydrogen peroxide (H_2_O_2_) (Henle and Linn, 1997) or potassium bromate (KBrO_3_) (Loft et al., 1998) (Fig. 2, F–H, Fig. S3, Fig. S4, and Fig. S5).

Using this experimental approach, we monitored for any genotoxic stress–induced changes in protein distribution pattern of the 15 Golgi–nuclear localizing DDR proteins. Initial inspection revealed that all three treatments triggered marked changes in both the subcellular localization distribution and the overall signal intensity of many of the 15 proteins tested. Two predominant redistribution patterns were observed: either in a Golgi-to-nuclear manner or, conversely, in a nucleus-to-Golgi direction. To quantify these shifts, we measured fluorescence intensities in Golgi and nuclear masks and calculated a Golgi-to-nuclear distribution ratio for each protein. Ratios <1 indicate Golgi-to-nuclear redistribution, whereas ratios >1 reflect nucleus-to-Golgi shifts. This approach normalized for differences in protein abundance and cell-to-cell heterogeneity.

The quantifications of the Golgi–nuclear ratio revealed a correlation between the redistribution patterns of these DDR proteins and their subcellular distribution with the Golgi complex. Here, treatment with DOX (Fig. 2, F and G; and Fig. S3, A–D) resulted in a predominantly consistent localization pattern change, where *cis*-Golgi DDR proteins (3 out of 4) shifted from the Golgi to the nucleus, whereas *trans*-Golgi proteins (7 out of 8) displayed a redistribution pattern from the nucleus to the Golgi. Conversely, exposure to H_2_O_2_ (Fig. 2, F and H; and Fig. S4) or KBrO_3_ (Fig. S5) elicited the opposite response, with the majority of *trans*-Golgi DDR proteins (7 out of 8) displaying a redistribution from the Golgi compartment to the nucleus, while 2 out of the 4 *cis*-Golgi DDR proteins displayed a redistribution pattern, from the nucleus to the Golgi.

When analyzed by pathway annotation, broad distinctions also emerged, BER proteins (TOPORS and LIG1) and MMEJ factor POLQ shifted from nucleus to Golgi in response to DOX, whereas the MMR protein MSH6 and HR-associated proteins (RAD51C and NBN) redistributed from Golgi to nucleus (Fig. 2 G and Fig. S3). Specifically, with DOX treatment, BER and MMEJ proteins shifted from the nucleus to the Golgi, while MMR and HR proteins relocated from the Golgi to the nucleus. Conversely, this trend was reversed with H_2_O_2_ or KBrO_3_ treatments (Fig. 2 H, Fig. S4, and Fig. S5).

While pathway-level trends were evident, some proteins (for example, CCAR1) exhibited similar directionality under DOX and H_2_O_2_, underscoring that pathway membership is not exclusive and many DDR factors act in multiple contexts. Nonetheless, these findings demonstrate that DDR proteins redistribute between Golgi and nucleus in a manner that depends on both the type of DNA lesion and their steady-state Golgi-cisternal localization.

### Redistribution of RAD51C Golgi fraction is required for the formation of RAD51C repair nuclear foci and is dependent on the kinase ATM

To mechanistically dissect the molecular basis of this Golgi–nucleus link, we focused on the HR repair protein RAD51C. As a member of the RAD51 paralog family, RAD51C is essential for regulating HR-mediated repair of DSBs. Previous studies have demonstrated RAD51C’s involvement across multiple stages of HR repair: promoting the DNA damage checkpoint (Badie et al., 2009), aiding in RAD51 filament formation, stabilizing replication forks, and resolving Holliday junctions to complete repair (Prakash et al., 2022; Greenhough et al., 2023; Rawal et al., 2023). RAD51C also functions in replication stress responses (Somyajit et al., 2012) underscoring its versatility. This multifunctionality, combined with a substantial Golgi-localized population compared with other DDR proteins in our screen, prompted us to investigate RAD51C as a representative candidate for understanding how Golgi-associated DDR factors are dynamically recruited to repair complexes.

**Figure S6.**
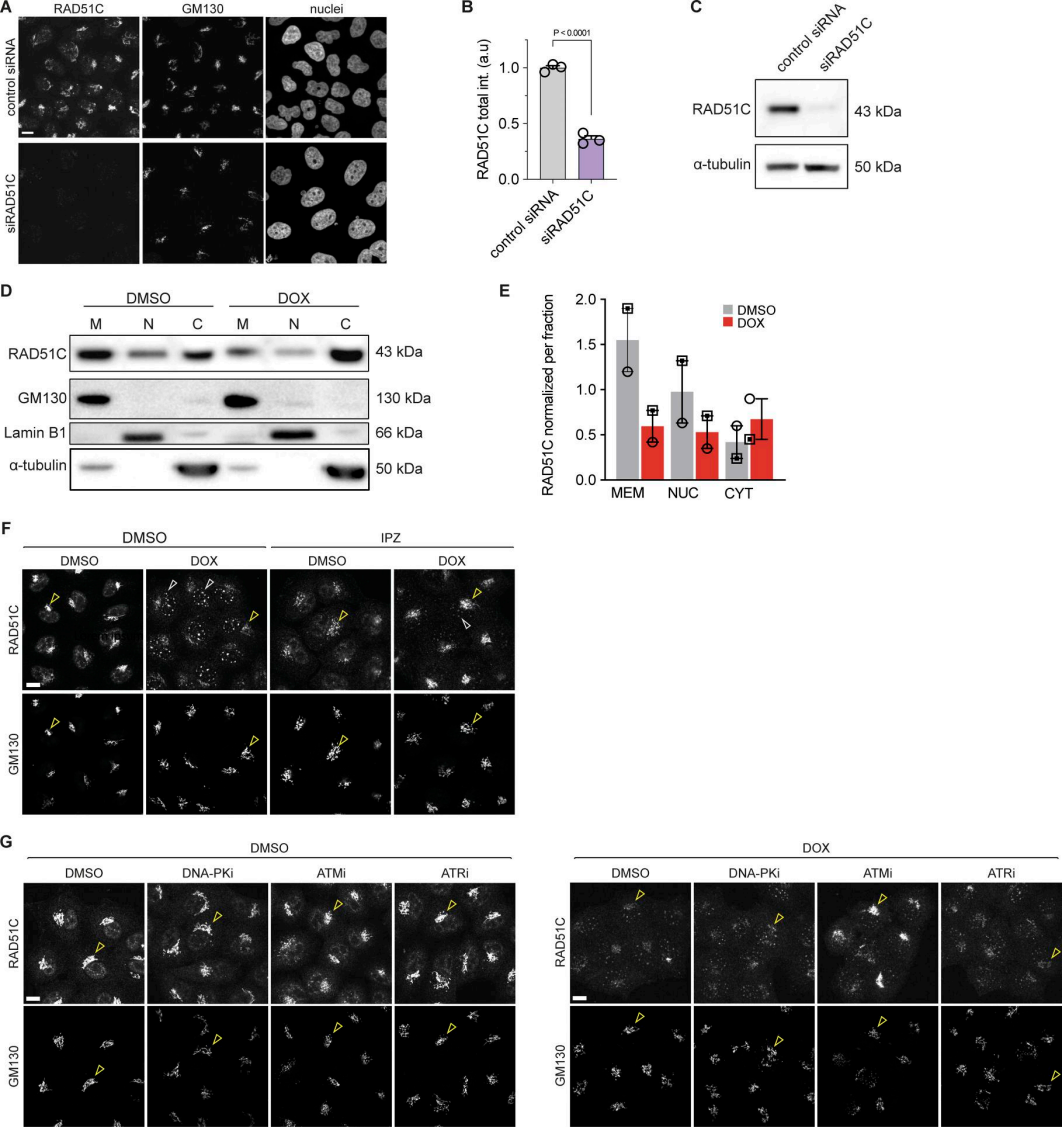
**Validation of RAD51C subcellular localization and population redistribution upon treatment with IPZ and inhibition of DDR signaling, related to Fig. 3. (A)** Representative images showing the specificity of the RAD51C antibody ab72063 by siRNA-mediated depletion. HeLa-K cells were transfected with control or RAD51C siRNA and stained with antibodies against RAD51C (ab72063) and GM130; DNA was stained with Hoechst 33342. Scale bar, 10 μm. **(B)** Quantification of RAD51C sum intensity after RAD51C depletion from A. Data represent the mean ± SEM. (*n* = 3 biologically independent experiments with a total of 831 cells analyzed). **(C)** Western blot analysis showing the RAD51C protein level, in control and RAD51C-depleted HeLa-K cells, detected with RAD51C antibody ab95069 (*n* = 3 biologically independent experiments). Statistical significance was determined using a two-tailed unpaired Student’s *t* test. **(D)** Western blot analysis showing the subcellular distribution of RAD51C in membrane (M), nuclear (N), and cytoplasmic (C) fractions from HeLa-K cells treated with DMSO or DOX (40 μM; 3 h). Fraction purity was confirmed using GM130 (membrane), Lamin B1 (nuclear), and α-tubulin (cytoplasmic) as markers. The protein levels were normalized against the respective fraction control (RAD51C^mem^/GM130; RAD51C^nuc^/Lamin B1; RAD51C^cyt^/tubulin). Data represent the mean ± SEM (*n* = 2 biologically independent experiments). **(E)** Quantification of RAD51C membrane-nuclear distribution ratio after treatment with DOX, calculated from isolated fractions. Data represent mean ± SEM. (*n* = 2 biologically independent experiments.) **(F)** Representative images of the RAD51C protein redistribution upon treatment with IPZ and DOX. HeLa-K cells were stained with antibodies against RAD51C and GM130. Cells were treated with DMSO or IPZ prior to a 3-h treatment with DOX. Yellow arrowheads denote the Golgi membrane; white denotes nuclear foci. Scale bar, 10 μm. The DMSO control image shown in this panel is the same control image shown in Fig. 3 K, as these panels derive from the same experiment and were split between the main and supplemental figures for comparison with the corresponding IPZ and DOX treatment conditions. **(G)** Representative images of the RAD51C protein redistribution upon treatment with DDR signaling inhibitors and DOX. HeLa-K cells were treated with DMSO (control) alone or with ATM inhibitor (KU55933), ATR inhibitor (VE-821), or DNA-PK inhibitor (NU7441) prior to a 3-h treatment with DOX. Yellow arrowheads denote the Golgi membrane; white denotes nuclear foci. Scale bars, 10 μm. The DMSO control image shown in this panel is the same control image shown in Fig. 3 N, as these panels derive from the same experiment and were split between the main and supplemental figures for comparison with the corresponding inhibitor and DOX treatment conditions. Source data are available for this figure: SourceData FS6.

Following the identification of RAD51C as a dual-localizing DDR protein in our initial HPA-based antibody screen (using RAD51C antibody, HPA061958), we sought to further validate its localization using additional commercial antibodies targeting distinct RAD51C epitopes. Our immunofluorescence experiments using RAD51C antibody, ab72063 across several cell lines confirmed RAD51C enrichment in a juxtanuclear compartment co-localizing with the Golgi marker GM130 (indicated by the yellow arrowhead; Fig. 3, A and B), together with diffuse cytoplasmic staining and discrete nuclear foci (indicated by white arrowheads; Fig. 3, A and B). The specificity of the antibodies was confirmed by the depletion of RAD51C in HeLa Kyoto (HeLa-K) cells (Fig. S6, A and B). To corroborate these results biochemically, we fractionated HeLa-K cell lysates into nuclear, membrane, and cytoplasmic compartments using RAD51C antibody, ab95069, whose specificity was confirmed by siRNA-mediated depletion (Fig. S6 C). RAD51C was detected in all fractions, with the majority present in the membrane and cytoplasmic pool (Fig. 3, C and D), consistent with the immunofluorescence data. Together, the consistent localization pattern observed across three independent RAD51C antibodies (HPA061958, ab72063, and ab95069) provides orthogonal confirmation of the dual Golgi and nuclear localization. The RAD51C antibodies ab72063 and ab95069 were used for all subsequent immunofluorescence and biochemical assays, respectively.

Although RAD51C has been extensively characterized biochemically and structurally, few studies have systematically examined its subcellular distribution. Earlier work has largely focused on *in vitro* complex formation and DNA repair mechanisms, rather than on the spatial distribution, likely contributing to the Golgi-associated pool remaining unrecognized. Notably, previous work, particularly Badie et al. (2009), showed a juxtanuclear RAD51C signal (see Fig. 1 D of that study), although this localization was not further discussed. Building on this overlooked observation and having now established the steady-state Golgi localization of RAD51C, we set out to examine its compartmental dynamics following DSB induction with DOX. Upon drug addition (Fig. 3, E–I), we observed an overall decrease in RAD51C protein level, with the Golgi-localized fraction (co-localized with GM130, yellow arrowheads) decreasing more rapidly than the nuclear fraction. Concurrently, the diffuse nuclear RAD51C pattern changed into distinct nuclear foci (white arrowheads), which became more pronounced with longer DOX exposure (Fig. 3 E).

Quantitative analysis of these experiments allowed us to measure the changes in RAD51C levels at the Golgi (Fig. 3 F) and the nuclear compartment (Fig. 3 G) and in the cytoplasm (Fig. 3 H) and to calculate a RAD51C Golgi–nuclear distribution ratio (Fig. 3 I). This redistribution was further validated by subcellular fractionation (Fig. 3 J and Fig. S6 D). Similarly, the total protein level of RAD51C was observed to decrease after 3-h treatment, with a much larger reduction in the membrane compartment when compared with the nuclear fraction. Overall, in both experiments, the RAD51C Golgi–nucleus distribution ratio was seen to decrease significantly after 3-h DOX treatment in both the immunofluorescence and biochemical assay (Fig. 3, I and J); although the cytoplasmic signal differed in direction between the two readouts: a modest decrease in the IF cytoplasmic compartment (Fig. 3 H) and a modest increase in the biochemical cytoplasmic fraction (Fig. S6 D).

To identify a mechanism for RAD51C redistribution between compartments, we induced DSBs using DOX while inhibiting importin-β–mediated nuclear import with the small molecule inhibitor importazole (IPZ) (Soderholm et al., 2011). IPZ treatment (Fig. 3, K–M and Fig. S6 F; Golgi fraction marked with yellow arrowheads; nuclear foci marked with white arrowheads) inhibited the DOX-induced RAD51C redistribution. Instead, the majority of the protein population remained co-localized with the Golgi marker, GM130, and nuclear foci formation was significantly inhibited (Fig. 3 K). IPZ alone did not alter RAD51C distribution relative to DMSO control, indicating that the inhibitor does not perturb steady-state localization (Fig. S6 F). However, under DOX plus IPZ, RAD51C remained Golgi localized rather than redistributing to the nucleus, suggesting that importin-β–dependent transport contributes to, or is tightly coupled with, the RAD51C dissociation step.

We next sought to test whether the phosphorylation of DDR kinases mediate RAD51C redistribution in response to DSBs. The three master kinases that are active in response to DNA damage are ATM, ataxia telangiectasia and Rad3-related (ATR), and DNA-PK (Ciccia and Elledge, 2010). Cells were treated with phosphorylation inhibitors specific for each kinase and RAD51C redistribution was analyzed following DOX treatment (Fig. 3, N–P and Fig. S6 G; Golgi fraction marked with yellow arrowheads). DOX combined with the ATM phosphorylation inhibitor, KU55933 (Hickson et al., 2004), significantly inhibited RAD51C redistribution, with the majority of the protein remaining co-localized with GM130 and nuclear foci formation was blocked (Fig. 3 N). In contrast, the combined treatment of DOX with ATR or DNA-PK phosphorylation inhibitors, VE-821 (Fokas et al., 2012) and NU7441 (Tavecchio et al., 2012), respectively, had no apparent impact on the redistribution of RAD51C when compared with cells treated with DOX only. Inhibitor-only treatments did not alter RAD51C localization (Fig. S6 G).

**Figure S7.**
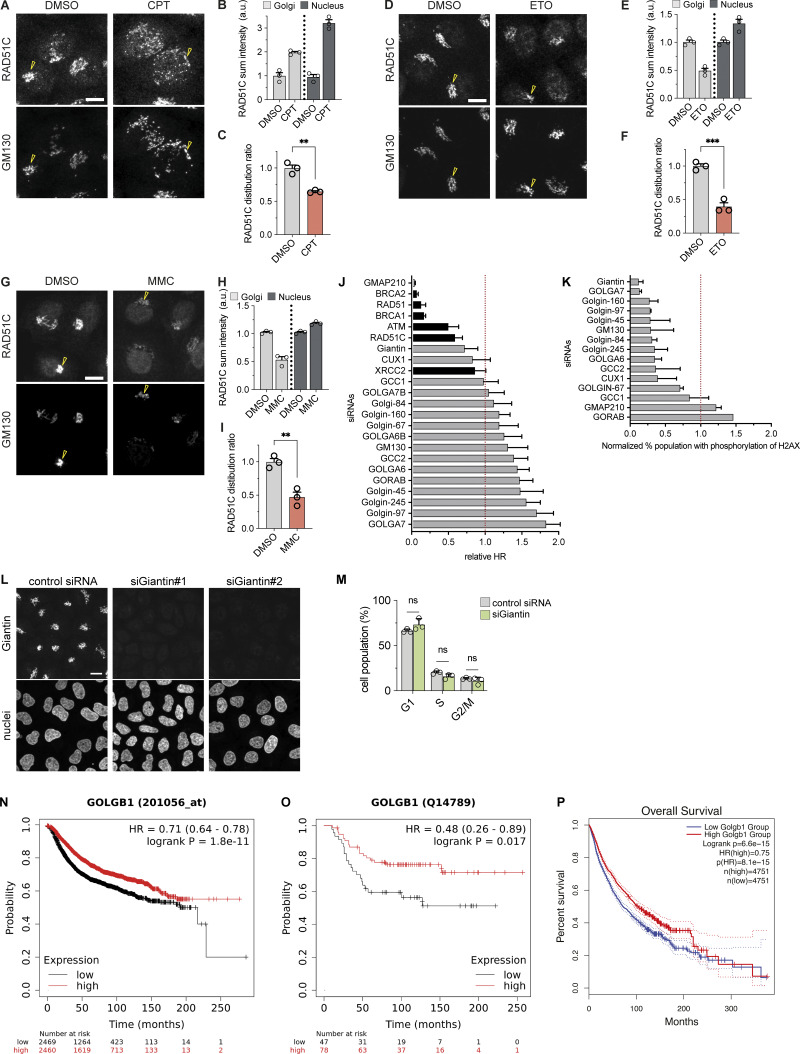
**RAD51C protein localization and redistribution following treatment with additional DNA damage agents, related to Fig. 3, impact of giantin and other golgins on DNA repair, cell cycle, and patient survival, related to Figs. 4 and 5. (A)** Representative images of HeLa-K cells stained with antibodies against RAD51C and GM130 after treatment with CPT (0.1 μM) for 16 h followed by media change for 2 h. Yellow arrowheads denote the Golgi membrane. Scale bar, 10 μm. **(B)** Quantification of RAD51C distribution between the Golgi and nuclear compartments after CPT treatment. **(C)** Quantification of RAD51C distribution ratio between the Golgi and nuclear compartments after CPT treatment. **(D)** Representative images of HeLa-K cells stained with antibodies against RAD51C and GM130 after treatment with ETO (50 μM) for 16 h followed by media change for 2 h. Yellow arrowheads denote the Golgi membrane. Scale bar, 10 μm. **(E)** Quantification of RAD51C distribution between the Golgi and nuclear compartments after ETO treatment. **(F)** Quantification of RAD51C distribution ratio between the Golgi and nuclear compartments after ETO treatment. **(G)** Representative images of HeLa-K cells stained with antibodies against RAD51C and GM130 after treatment with MMC (5 μM) for 16 h followed by media change for 2 h. Yellow arrowheads denote the Golgi membrane. Scale bar, 10 μm. **(H)** Quantification of RAD51C distribution between the Golgi and nuclear compartments after MMC treatment. **(I)** Quantification of RAD51C distribution ratio between the Golgi and nuclear compartments after MMC treatment. Data represent the mean ± SEM (*n* = 3 biologically independent experiments with more than 600 cells analyzed per treatment). Statistical significance was determined using a two-tailed unpaired Student’s *t* test comparing treated with untreated control. **P < 0.01; ***P < 0.001. **(J)** Reanalyzed siRNA screen data (Adamson et al., 2012) showing the relative HR repair rate upon systematic knockdown of the golgin protein family (grey) and HR complex proteins (black). The dataset is normalized to the negative control set at 1. **(K)** Reanalyzed siRNA screen data (Paulsen et al., 2009) showing the relative percent cell population with phosphorylation of H2AX upon systematic knockdown of the golgins. The datasets are normalized to the negative control set at 1. **(L)** HeLa-K cells were transfected with control, or giantin siRNAs for 72 h. The cells were stained with antibodies against giantin; nuclei were stained with Hoechst 33342. Scale bar, 10 μm. **(M)** Cell cycle profile of HeLa-K cells treated with giantin or control siRNA. Data represent the mean ± SEM. (*n* = 3 biologically independent experiments). **(N and O)** Kaplan–Meier survival plots in patients with high and low giantin expression in breast cancer, (N) at mRNA level (Győrffy, 2021) and (O) protein level (Ősz et al., 2021). Plots were generated using the KM Plotter database (https://kmplot.com). **(P)** Pan-cancer overall survival map for GOLGB1 expression across all available TCGA cohorts, generated using GEPIA2 (Tang et al., 2019). Tumor type–specific hazard ratios, sample sizes, and *P* values are provided in Table S3.

To confirm that these observations were a direct result of DSBs and not an off-target drug effect, we tested other DSB-causing agents (Jekimovs et al., 2014): camptothecin (CPT) (Fig. S7, A–C), etoposide (ETO) (Fig. S7, D–F), and mitomycin C (MMC) (Fig. S7, G–I). All treatments led to significant RAD51C redistribution from the Golgi to the nuclear compartment. Notably, CPT treatment (Fig. S7 A) caused a shift in nuclear RAD51C from a diffuse distribution to distinct foci, while ETO or MMC treatments (Fig. S7, D and G) increased the nuclear RAD51C population without obvious foci formation. These distinct nuclear phenotypes likely reflect lesion class and timing: CPT generates replication associated breaks that favor discrete HR foci, whereas ETO (Topo II poisoning) and MMC (interstrand cross-links) produce damage that, within our treatment window, increases nuclear RAD51C with a less pronounced punctate pattern.

### Golgi localization of RAD51C is dependent on the golgin giantin

**Figure 4.**
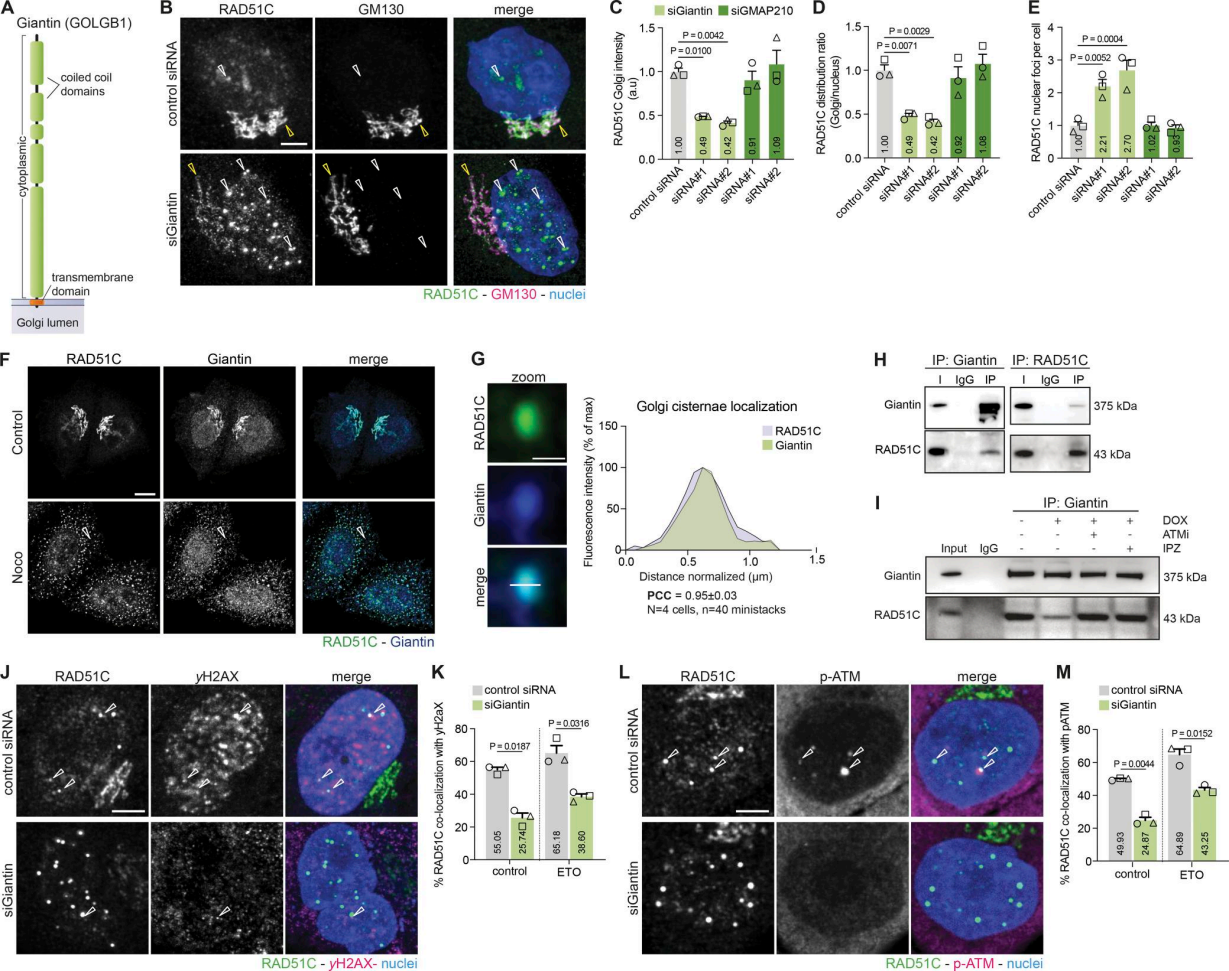
**RAD51C Golgi localization is dependent on the golgin protein, giantin.** Immunofluorescence in this figure was performed using RAD51C antibody ab72063; co-IP in H and I used RAD51C antibody ab95069 and giantin antibody AF8159. **(A)** Schematic diagram highlighting the domain organization of giantin. **(B)** RAD51C protein redistribution upon depletion of giantin. HeLa-K cells were transfected with either control or giantin siRNAs for 72 h, then immunostained for RAD51C and the Golgi marker GM130. Yellow arrowheads mark the Golgi, and white arrowheads denote RAD51C nuclear foci. Scale bars, 10 μm. **(C–E)** Quantifications of RAD51C localization following depletion of giantin or GMAP210: (C) total Golgi-associated RAD51C intensity, (D) the Golgi-to-nucleus RAD51C intensity ratio, and (E) the number of nuclear RAD51C foci per cell. Data are presented as mean ± SEM. (*n* = 3 biologically independent experiments, 1,445 total cells). Statistical significance determined by one-way ANOVA with Tukey’s post hoc test. **(F)** Golgi-cisternal co-localization analysis. HeLa-K cells treated with nocodazole (33 μM, 3 h), fixed, and stained with antibodies against giantin and RAD51C. Scale bar, 10 μm. **(G)** Enlarged view of single isolated mini-stack from F; white line across the stack was used for line-scan analysis. Scale bar, 1 μm. **(H and I)** co-IP of endogenous giantin with RAD51C under control conditions (H) or after DOX treatment and co-treatments with ATM inhibitor (ATMi) and IPZ (I) in HeLa-K extracts. **(J–M)** Co-localization experiment of cells treated with control siRNA, or giantin siRNA under control conditions or after treatment with ETO. Cells stained with antibodies against RAD51C (green) and HR DDR markers (red): (J) γ-H2AX and (L) p-ATM. Co-localizing foci are indicated by arrowheads. Scale bars, 10 μm. Quantification of percentage RAD51C foci co-localizing with (K) γ-H2AX and (M) p-ATM. Data represent the mean ± SEM. (*n* = 3 biologically independent experiments with at least 200 cells and at least 550 RAD51C nuclear foci analyzed for the co-localization experiments for each condition). Statistical significance determined by two-tailed Student’s *t* test. Source data are available for this figure: SourceData F4.

To identify potential Golgi membrane anchors for RAD51C, we surveyed publicly available genome-wide siRNA screens of HR and DDR regulators and identified two independent datasets (Paulsen et al., 2009; Adamson et al., 2012) with broad coverage of Golgi-resident proteins. Mining these screens, we found that multiple golgin family members emerged as hit candidate genes. In particular, knockdown of giantin and GMAP210 led to a marked reduction in HR repair rates (Adamson et al., 2012) (golgin results summarized in Fig. S7 J), and the depletion of giantin strongly inhibited H2AX phosphorylation, a crucial event in DDR signaling regulation (Paulsen et al., 2009) (golgin results summarized in Fig. S7 K). Given the structural properties of golgins (Fig. 4 A), which are predominantly coiled-coil proteins anchored to the Golgi membrane by their carboxy terminus and projected into the surrounding cytoplasm (Witkos and Lowe, 2015), they are ideally suited for capturing or tethering nearby membranes and potentially retaining DDR proteins such as RAD51C.

To investigate whether RAD51C localization is dependent on these golgins, we performed siRNA-mediated depletion of giantin and GMAP210 and compared their effects on RAD51C distribution to a control siRNA treatment (Fig. 4, B–E). GMAP210 depletion had no significant effect on RAD51C distribution, whereas giantin knockdown led to a notable redistribution of RAD51C (Fig. 4 B). Specifically, we observed a marked decrease in RAD51C co-localization with the Golgi marker GM130 (yellow arrowheads) and an increase in nuclear RAD51C localization with distinct bright nuclear foci appearing (white arrowheads). Quantifications revealed a significant reduction of the RAD51C Golgi population (Fig. 4 C), and the distribution ratio (Fig. 4 D) was reduced by more than half. The number of RAD51C foci (Fig. 4 E) increased by more than twofold upon depletion of giantin with either siRNA. There was no significant difference in RAD51C Golgi intensity, distribution ratio or foci number (Fig. 4, C–E) between the GMAP210-depleted and control cells. Sub-Golgi cisternae localization analysis confirmed that giantin and RAD51C are distributed in a similar manner throughout the organelle (Fig. 4, F and G). The effective knockdown of giantin (Fig. S7 L) and GMAP210 was tested by immunofluorescence.

To determine if RAD51C physically interacts or is part of a larger complex with giantin we performed an immunoprecipitation (IP) assay (Fig. 4, H and I). Endogenous giantin and RAD51C were found to co-IP from HeLa-K protein extracts (Fig. 4 H). To explore the dynamics of this interaction in response to DNA damage, we induced DSBs and performed co-IP experiments (Fig. 4 I). Our results showed that RAD51C and giantin dissociate upon DSB induction. However, co-treatment with either an ATM inhibitor or IPZ, alongside DOX, prevented this dissociation, corroborating our earlier immunofluorescence observations (Fig. 3, E–P).

To gain insight into the nature of RAD51C foci induced by giantin depletion, we carried out co-localization assays to determine whether these structures contain standard DDR markers, phosphorylated H2AX (γ-H2AX) (Fig. 4, J and K) and phosphorylated ATM (p-ATM) (Fig. 4, L and M) under physiological conditions and induction of DSBs by ETO. Both proteins are well-established markers for DSB repair sites and are important for the recruitment of the HR repair machinery (Vítor et al., 2020). In cells treated with a control siRNA, we observed that approximately half of the RAD51C nuclear foci were decorated with either γ-H2AX or p-ATM (Fig. 4, J–M; co-localizing foci are denoted with an arrowhead), as previously described (Vítor et al., 2020). RAD51C foci induced by the depletion of giantin, however, showed significantly lower co-localization with both markers regardless of DSB induction. Overall, giantin-depleted cells showed fewer γ-H2AX and p-ATM nuclear foci, consistent with previous reports (Paulsen et al., 2009) (Fig. S7 K).

### Giantin depletion impairs ATM signaling and HR efficiency, increasing genomic instability and cell proliferation

**Figure 5.**
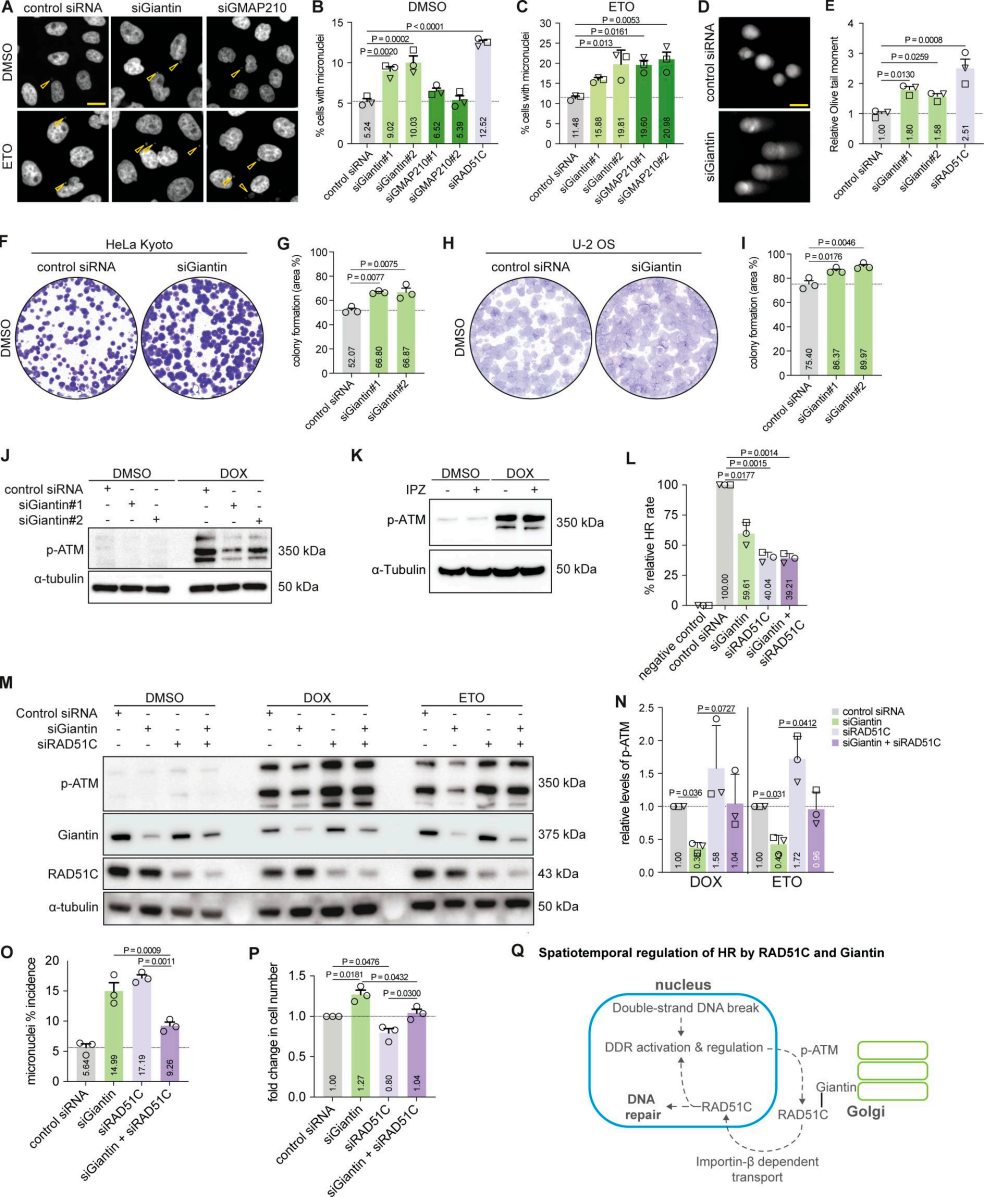
**Depletion of giantin impairs ATM signaling and HR efficiency, increasing genomic instability and cell proliferation. (A)** Representative images of HeLa-K cells with nuclei labeled with Hoechst 33342 following siRNA treatment. Yellow arrowheads denote micronuclei structures. Scale bar, 10 μm. **(B and C)** Quantification of micronuclei formation following siRNA treatment under (B) basal conditions and (C) after ETO-induced DNA damage (10 μM; 16 h followed by 6-h recovery). Note: siRAD51C is not shown in panel C as RAD51C depletion combined with ETO treatment resulted in insufficient cell viability for reliable quantification. Data represent the mean ± SEM (*n* = 3 biologically independent experiments with >10,000 cells analyzed). Statistical significance determined by one-way ANOVA with Tukey’s post hoc test. **(D)** Representative comet assay images measuring genomic DNA fragmentation in HeLa-K cells treated with control, giantin, or RAD51C targeting siRNA. Scale bar, 50 μm. **(E)** Quantification of Olive tail moment from comet assay. Data represent the mean ± SEM (*n* = 3 biologically independent experiments). Statistical significance determined by one-way ANOVA with Tukey’s post hoc test. **(F–I)** Colony formation assays in HeLa-K (F and G) and U-2 OS (H and I) cells transfected with control or giantin siRNAs. Representative images (F and H) and quantification of colony area coverage (G and I). Data represent the mean ± SEM (*n* = 3 biologically independent experiments). Statistical significance determined by one-way ANOVA with Tukey’s post hoc test. **(J)** Representative western blot analysis of HeLa-K cells transfected with control and giantin siRNA and treated with DOX (40 μM; 3 h). Extracts were prepared and immunoblotted as indicated (*n* = 2 biologically independent experiments). **(K)** Western blot of HeLa-K cells treated with DOX (40 μM; 3 h) and IPZ (20 μM; 3 h). (*n* = 2 biologically independent experiments). **(L)** DR-GFP reporter assay measuring HR efficiency following siRNA-mediated depletion of giantin, RAD51C, or both. HEK293T-DR-GFP cells were transfected with the indicated siRNAs for 72 h, followed by transfection of the I-SceI expression plasmid pCBASce for 48 h. Cells transfected with siRNAs but without pCBASce served as a no-DSB background control (shown as negative control). GFP-positive cells were quantified by automated high-content imaging, and the percentage of GFP-positive cells was normalized to the nontargeting siRNA control set to 1. Data represent the mean ± SEM (*n* = 3 biologically independent experiments). Statistical significance determined by one-way ANOVA with Tukey’s post hoc test. **(M and N)** Western blot of HeLa-K cells transfected with siRNAs against giantin, RAD51C, or a combination of both followed by treatment with DMSO, DOX (40 μM; 3 h) (M), or ETO (10 μM; 3 h) (N) with associated quantification showing the relative levels of pATM; (*n* = 3 biologically independent experiments); data represent the mean ± SEM statistical significance determined by one-way ANOVA with Tukey’s post hoc test. **(O and P)** Quantification of micronuclei formation and fold change in cell number after siRNA treatments (mean ± SEM, *n* = 3, ≥1,000 cells per condition). Statistical significance determined by one-way ANOVA with Tukey’s post hoc test. **(Q)** Proposed model for the regulation of HR-mediated repair through the activation of RAD51C at the Golgi complex. RAD51C, a regulatory HR protein, is anchored to the Golgi through its interaction with the cytoplasmic tail of giantin, in response to DSBs; this RAD51C Golgi population redistributes to form nuclear foci. This response requires importin-β–mediated nuclear import and the phosphorylation of ATM protein kinase. We propose that the Golgi functions as a spatiotemporal timing module that gates RAD51C (and other DDR factors) availability to ensure proper HR and DNA-damage signaling. Source data are available for this figure: SourceData F5.

Having shown that giantin regulates Golgi tethering of RAD51C and its DNA damage–triggered release, we asked whether depletion of giantin impacts genome stability and DDR signaling. We analyzed GMAP210 alongside giantin because prior HR/DDR screens implicated both golgins (Paulsen et al., 2009; Adamson et al., 2012). As a first readout, we assessed micronuclei formation, a well-established marker of genotoxic stress (Fig. 5, A–C) (Krupina et al., 2021). Under basal conditions, depletion of giantin or RAD51C significantly increased the percentage of cells displaying micronuclei and aberrant nuclear structures compared with controls, while GMAP210 knockdown had no significant effect (Fig. 5 B). To test repair capacity, we induced DNA damage and allowed recovery (Fig. 5 C). Strikingly, after recovery both GMAP210 and giantin knockdown led to a sustained and significant increase in micronuclei formation after recovery, indicating persistent genomic instability and defective repair dynamics. RAD51C-depleted cells could not be assessed under these conditions due to insufficient viability following combined RAD51C depletion and ETO treatment. Because our mechanistic model centers on a giantin–RAD51C tether and the strongest basal phenotype mapped to giantin, subsequent assays focus on giantin.

To corroborate basal genomic instability, we performed comet assays (Møller, 2018) to measure levels of fragmented genomic DNA (Fig. 5, D and E). Cells with defective DNA repair or exposed to DNA-damaging agents display long comet tails, whereas healthy cells show shorter or no tails. Knockdown of giantin or RAD51C resulted in notably longer comet tails compared with control siRNA treatment (Fig. 5 D). Quantification revealed a 2.5-fold increase in Olive tail moment for RAD51C-depleted cells, and 1.6- to 1.8-fold increases for giantin-depleted cells (siGiantin#1 and siGiantin#2, respectively) (Fig. 5 E).

We next examined the long-term consequences for cell proliferation and colony formation in HeLa-K and U-2 OS cells (Fig. 5, F–I). Giantin knockdown significantly enhanced both proliferation and colony formation. To determine whether these effects were associated with cell cycle changes, we analyzed the cell cycle profiles of giantin-depleted cells via FACS (Fig. S7 M). No significant cell cycle alterations were detected, suggesting that the increased proliferation is not due to major cell cycle perturbations.

Having observed that giantin knockdown increases basal genomic instability and displaces RAD51C from the Golgi, we next assessed ATM activation as a proximal readout of HR-linked DDR signaling (Fig. 5 J). In control siRNA-treated cells, DOX treatment increased p-ATM levels in response to DSBs. However, giantin-depleted cells (Fig. 5 J) showed significantly lower ATM phosphorylation levels under the same conditions. This is consistent with our earlier observations that RAD51C nuclear foci induced by giantin depletion show reduced co-localization with p-ATM (Fig. 4, L and M) and with the previously reported reduction in H2AX phosphorylation upon giantin knockdown in a genome-wide screen (Paulsen et al., 2009; Fig. S7 K).

To investigate whether the redistribution of HR factors from the Golgi to the nucleus plays a role in ATM signaling, we used IPZ to inhibit importin-β–mediated nuclear import and subsequently induced DSBs with DOX (Fig. 5 K). Here, we found that IPZ treatment did not significantly alter ATM signaling, regardless of the induction of DSBs. These findings suggest that the regulated redistribution of HR factors is less impactful on ATM signaling than their mislocalization, implying that giantin’s spatial regulation of HR factors is crucial for proper ATM activation. This dissociation of nuclear import from ATM activation argues that the giantin-dependent regulation of ATM signaling acts upstream of, or in parallel to, RAD51C nuclear translocation, rather than as a downstream consequence of altered nuclear pools.

Having shown that giantin’s spatial regulation of HR factors influences upstream DDR signaling, we next asked whether this spatial regulation also affects HR repair capacity itself. To directly measure HR efficiency, we employed the DR-GFP reporter assay, a well-established system (Pierce et al., 1999) in which I-SceI–induced DSBs are repaired by HR to restore GFP fluorescence (Fig. 5 L). Depletion of giantin reduced HR efficiency to ∼60% of control levels, consistent with the HR reduction upon giantin knockdown previously reported in a genome-wide HR screen (Adamson et al., 2012). RAD51C depletion reduced HR efficiency further, to ∼40% of control. Co-depletion of giantin and RAD51C reduced HR to levels comparable with RAD51C depletion alone, suggesting that the effect of giantin on HR efficiency is mediated primarily through RAD51C.

Having established that giantin depletion impairs both ATM signaling and HR efficiency, and that RAD51C is a primary mediator of this effect, we next asked whether co-depletion of RAD51C could rescue the broader phenotypes associated with giantin loss. To this end, we carried out co-depletion of RAD51C and giantin, along with single depletions (Fig. 5, M and N). Consistent with previous observations, giantin knockdown led to a significant decrease in ATM phosphorylation levels, while RAD51C knockdown increased ATM phosphorylation. Co-depletion of RAD51C and giantin restored ATM phosphorylation levels to those comparable with control cells. To validate the restoration of DDR signaling through co-depletion, we assessed genomic stability via micronuclei incidence (Fig. 5 O). Individual knockdowns of giantin and RAD51C elevated micronuclei formation, indicative of genomic instability, whereas co-depletion significantly reduced micronuclei incidence, approaching levels observed in control cells. Lastly, we evaluated relative cell number following siRNA treatment as a measure of proliferative capacity (Fig. 5 P) consistent with the colony formation data presented above (Fig. 5, F–I). While giantin knockdown increased relative cell number and RAD51C knockdown reduced it, co-depletion resulted in cell numbers comparable with control, further supporting the epistatic relationship between giantin and RAD51C.

## Discussion

In this study, we reveal evidence for a previously underappreciated and functionally meaningful spatiotemporal regulatory system connecting the Golgi complex and the nucleus, with broad potential implications for HR and other DNA repair pathways. Our analysis of HPA localization data (Thul et al., 2017) and experimental validation via siRNA knockdowns uncovered numerous double-localizing Golgi–nuclear proteins. Among these, we identified a cluster of DNA repair proteins that exhibit steady-state localization in both compartments. This cluster encompasses crucial regulatory proteins involved in various DNA repair pathways (Fig. 1 D), not only specific for HR-mediated DNA repair but also MMR, BER, and MMEJ, as well as other integral regulators of DNA repair cellular responses such as chromatin cohesion, ubiquitination, cell cycle regulation, and signaling, positioning the Golgi as a coordination node that links cytoplasmic organization with nuclear repair programs.

Our analysis revealed enrichment of these DNA repair proteins in distinct sub-Golgi regions, suggesting a compartmentalized platform where components of related DNA repair pathways gather to facilitate protein–protein interactions and tune the timing and specificity of downstream signaling. The enriched association of the Golgi scaffold giantin with HR factor RAD51C in particular is consistent with a structural or scaffolding role in organizing these repair proteins, where spatial sequestration could help preserve pathway fidelity by discouraging premature activation and by supporting timely recruitment of DDR components upon damage.

In response to genotoxic stress, we observed dynamic shuttling of DNA repair factors between the Golgi and the nucleus, dependent on the type of DNA damage. The correlation between steady-state Golgi localization and their redistribution patterns upon DNA damage is consistent with a role for the Golgi in temporally coordinating DDR. We propose that the Golgi serves as a hub from which DDR proteins are dynamically partitioned between compartments in a damage- and pathway-specific manner, released to the nucleus to support repair (as we demonstrate for RAD51C) or accumulated at the Golgi for sequestration or assembly (as observed for several other factors in our screen). In this model, spatiotemporal partitioning functions as a potential checkpoint that coordinates repair factor dosage and timing, thereby integrating cytoplasmic signals with nuclear lesions. Analogous behavior has been described for shuttling proteins such as breast cancer gene 1 (BRCA1), whose movements between nucleus and cytoplasm modulate DNA repair, cell cycle regulation, and apoptosis (Feng et al., 2004; Rodriguez et al., 2004; Thompson, 2010). By extension, Golgi-mediated spatiotemporal control of multiple factors may contribute to maintaining genomic stability.

To explore this spatiotemporal regulation, we outline a pathway linking the Golgi to HR repair (Fig. 5 Q). Based on our findings and consistent with established models, we propose that the pathway begins with the detection of DSBs in the nucleus by the MRN complex, which activates ATM kinase. Although canonical MRN function is indispensable for initiating ATM activation at DNA breaks, our data indicate that once ATM is activated in the nucleus, an ATM-dependent signal or possibly a fraction of active ATM can subsequently access the Golgi. This is consistent with emerging evidence that ATM binds phosphatidylinositol-4-phosphate at the Golgi membrane and that this Golgi-resident pool modulates the magnitude and kinetics of the nuclear DDR (Ovejero et al., 2023). Importantly, recent work suggests that Golgi-associated ATM is not merely a passive reservoir but is enzymatically active and capable of phosphorylating Golgi-resident substrates (Soulet et al., 2026, *Preprint*), providing a plausible mechanistic basis for how damage-induced ATM signaling could reach the Golgi to license RAD51C release. In this view, the Golgi acts downstream of the break sensor as an amplification and timing module rather than a primary sensor, representing an organelle level checkpoint that conditions release on damage context, akin to cytoplasmic ATM pools observed in oxidative stress responses (Shiloh and Ziv, 2013). While the role of the cytoplasm as a transit compartment in this process remains to be fully resolved, our imaging and biochemical readouts did not reveal major accumulation of RAD51C in the cytoplasm following damage.

Within this framework, our data place giantin as a spatial modulator of this Golgi–nuclear timing system. Giantin depletion increases basal genomic instability, accelerates cell proliferation, reduces ATM activation after DSB induction, and displaces a RAD51C pool to the nucleus. RAD51C depletion alone elevates genome instability but increases p-ATM after damage, while co-depletion of RAD51C with giantin partially restores p-ATM and genome stability toward control and attenuates the giantin-dependent proliferation increase. Direct measurement of HR efficiency using the DR-GFP reporter assay confirms this functional dependency: Giantin depletion reduces HR to ∼60% of control, and co-depletion with RAD51C produces no further reduction beyond RAD51C depletion alone, consistent with RAD51C as the principal mediator of giantin’s effect on HR. Together, these findings establish a functional epistatic relationship between giantin and RAD51C, although they do not formally prove that the physical interaction is the sole mechanistic basis of the phenotype, and contributions from additional giantin-associated factors cannot be excluded. Golgins are well established as multi-cargo scaffolding platforms with diverse roles in membrane trafficking and cytoplasmic signaling. The DDR role uncovered here positions giantin at the interface of these established cytoplasmic functions and DNA repair, offering a potential point of integration between organelle homeostasis, trafficking, and the DDR. Separation-of-function experiments, including domain mapping of the giantin–RAD51C interface and the use of interaction-deficient mutants, will be important to dissect this further.

RAD51C’s established functions can plausibly account for part of the giantin-loss phenotype. As a member of the RAD51 paralog complexes BCDX2 and CX3, RAD51C orchestrates RAD51 filament formation and strand invasion required for DSB repair, stabilizes replication forks, and contributes to checkpoint signaling through interaction with ATR–RPA and CHK2 (Badie et al., 2009; Somyajit et al. 2012; Somyajit et al. 2015; Sullivan and Bernstein 2018; Berti et al., 2020; Rein et al., 2021; Prakash et al., 2022). Mislocalization could undermine these processes: prematurely released or improperly retained RAD51C may produce disorganized filaments and error-prone repair, and dysregulated checkpoint signaling could allow cells to progress through the cell cycle despite harboring lesions. Importantly, the consistent detection of a nuclear RAD51C pool at steady state across all cell lines examined indicates that the Golgi pool does not represent the entirety of cellular RAD51C, but coexists with a constitutive nuclear fraction. The relative contributions of these pools likely reflect a complex and context-dependent balance, with the nuclear fraction potentially supporting constitutive replication-associated roles and the Golgi-tethered fraction acting as a damage-responsive reserve whose regulated release is essential, as demonstrated by the aberrant foci and impaired ATM signaling that follow its premature displacement upon giantin depletion. Further dissection of these pools, including their kinetics and functional specialization across cell types and damage contexts, will be a valuable direction for future work. Whether Golgi association and damage-induced release involve RAD51C alone or extend to other BCDX2 or CX3 members is a particularly compelling open question, and the nuclear localization and export signals previously identified in RAD51C (Miller et al., 2005) provide tractable targets for future mutagenesis studies.

Beyond RAD51C, our screen identified other DDR regulators with dual Golgi–nuclear localization, notably the HR regulator NBN, and candidates spanning chromatin regulation, ubiquitination, and cell cycle signaling. These observations argue for a cohort model in which multiple Golgi-proximal factors are clustered at steady state and released with tuned timing, such that their combined availability, rather than any single protein alone, shapes HR pathway behavior. In this view, the Golgi provides a spatiotemporal checkpoint that modulates factor dosage and order of arrival at lesions. Our dataset supports this timing perspective through changes in upstream signaling, genome-stability metrics, and direct HR measurements. However, precisely how the Golgi-released pool of RAD51C contributes to repair complex assembly and HR execution in the nucleus remains to be established. Systematic follow-up should broaden the catalog of Golgi-associated DDR factors, benchmark their relative contributions, and couple temporal proteomics with multiplex perturbations to resolve how joint release kinetics influence HR fidelity and fork protection and restart.

In a clinical context, RAD51C is a well-established hereditary breast and ovarian cancer susceptibility gene (Meindl et al., 2010), providing direct relevance for a study centered on RAD51C spatial regulation. Perturbation of golgins, including giantin, has similarly been linked to elevated breast cancer risk (Wang et al., 2020; Mathioudaki et al., 2021; Craven et al., 2021; Pipek et al., 2023; Leighton et al., 2023; Ghannoum et al., 2023), and lower Giantin expression is associated with decreased patient survival in breast cancer at both mRNA and protein levels (Fig. S7, N and O) (Győrffy, 2021; Ősz et al., 2021). To place this in a broader context, we extended the survival analysis of GOLGB1 across all available cohorts in The Cancer Genome Atlas (TCGA) using GEPIA2 (Tang et al., 2019). In the pooled pan-cancer analysis, higher GOLGB1 expression is significantly associated with improved overall survival (Fig. S7 P). When stratified by tumor type (Table S3), significant associations are observed in kidney renal clear cell carcinoma and lower-grade glioma with the opposite effect. Although the larger KM Plotter cohort showed a significant association between low GOLGB1 expression and worse breast cancer survival (Fig. S7, N and O), this signal was not reproduced in the smaller TCGA-BRCA cohort, consistent with reduced statistical power. These context-dependent associations, together with reports implicating additional Golgin family members across various cancers (Baschieri et al., 2015; Bhat et al., 2017), are compatible with a broader tumor context-dependent role for Golgi scaffolding in genomic integrity. This raises the prospect of therapeutically modulating organelle-level checkpoints either by tuning Golgi-dependent timing of factor release or by leveraging Golgi–nuclear communication to sensitize tumors to genotoxic agents.

This study provides, to our knowledge, the first systematic characterization of the Golgi complex as a spatiotemporal coordination node for DNA repair factors, with regulated release of a Golgi-anchored pool contributing to the nuclear DDR. Several questions remain open and represent natural directions for future investigation. Direct visualization of RAD51C translocation dynamics in living cells was not achievable here, as both CRISPR-mediated endogenous tagging and ectopic expression strategies compromised physiological behavior, a difficulty common to RAD51 paralog complexes more broadly. Whether the nuclear RAD51C that accumulates after genotoxic stress originates specifically from the Golgi-anchored pool remains to be formally established, although reciprocal redistribution kinetics, biochemical fractionation, and inhibitor data are collectively consistent with this model. Finally, the sub-Golgi localization patterns reported here for 15 DDR proteins represent an initial map that invites systematic expansion. Spatially restricted proximity labeling approaches such as giantin-TurboID, together with new tagging strategies compatible with essential, complex-embedded proteins, will be key tools to address these questions. Together with our broader observation that many proteins dually localize to the Golgi and nucleus, spanning membrane trafficking, RNA metabolism, and lipid regulation, these findings support the concept of the Golgi as a dynamic integration hub for multiple essential pathways, whose regulatory roles extend well beyond its classical functions in secretion and posttranslational modification. Collectively, this argues for a shift from concentration-based to timing- and compartment-aware models of the DDR and identifies the Golgi–nucleus axis as a previously unrecognized target for understanding and potentially modulating the DDR in cancer.

## Materials and methods

### Antibodies and chemicals

The following commercial antibodies and chemicals were used in this study, including RAD51C (ab72063; Abcam; 1:500 for immunostaining) and (ab95069; Abcam, 1:2,000 for western blotting; 1:500 for IP), GOLGB1/Giantin (AF8159; R&D systems; 1:500 for immunostaining and IP, 1:2,000 for western blotting) and (G1/M1; Enzo Life Sciences, 1:500 for immunostaining), GM130 (610822; BD Biosciences; 1:500 for immunostaining, 1:2,000 for western blotting), TGN46 (AHP500GT; Bio-Rad; 1:1,000 for immunostaining; 1:2,000 for western blotting), ATM (MA1-23152; Invitrogen; 1:2,000 for western blotting), pATM Ser1981 (MA1-2020; Invitrogen; 1:500 for immunostaining; 1:2,000 for western blotting), CHK2 pThr68 (PA5-17818; Invitrogen; 1:2,000 for western blotting), DNA-PKcs (Ser2056) (PA5-78130; Invitrogen, 1:2,000 for western blotting), gamma-H2AX pSer139 (613402; BioLegend; 1:500 for immunostaining, 1:2,000 for western blotting), alpha-tubulin (MS-581; Thermo Fisher Scientific; 1:10,000 for western blotting), Lamin B1 (ab16048; Abcam; 1:2,000 for western blotting), doxorubicin (ab120629; Abcam); importazole (SML0341; Sigma-Aldrich), KU55933 (ATMi, SML1109; Sigma-Aldrich), VE-821 (ATRi, HY-14731; MedChemExpress), NU7441 (DNA-PKi, HY-11006; MedChemExpress), etoposide (ab120227; Abcam), mitomycin C (M7949; Sigma-Aldrich), hydrogen peroxide (H1009; Sigma-Aldrich), potassium bromate (104912; Merck Millipore), camptothecin (ab120115; Abcam), SYBR Gold (S11494; Invitrogen), and crystal violet (C0775-100G; Sigma-Aldrich).

### Cell lines, cell culture, and siRNA transfection

HeLa-K, U-2 OS, and MCF7 cells were cultured in DMEM (Life Technologies) supplemented with 10% FBS (Invitrogen) and 1% L-glutamine (Invitrogen). Cells were routinely tested for mycoplasma contamination by PCR. siRNA transfections were performed with Lipofectamine 2000 (Invitrogen) using Silencer Select siRNAs (Ambion) according to the manufacturer’s instructions. Transfections were carried out for 72 h, and the final siRNA concentrations used were 15 nM for all siRNAs. Giantin siRNA-1: s5951 and siRNA-2: s5953; GMAP210 siRNA-1: s17811 and siRNA-2: s17812; RAD51C siRNA: s11737.

### Localization validation siRNA assay

A custom siRNA library targeting our proteins of interest (Ambion) was designed and prepared in 96-well glass-bottom plates (Miltenyi Biotec) using a protocol for solid phase reverse transfection as previously described (Erfle et al., 2008; Stadler et al., 2012). Nontargeting siRNA was used as a negative control. 72 h after cell seeding, cells were fixed with 4% PFA, permeabilized with 0.1% Triton-X 100 and immunostained against the HPA antibodies of interest and a Golgi marker, GM130, each diluted to 2 μg/ml in 4% FBS in 0.1% Triton-X100. Hoechst 33342 was used as a nuclear stain. siRNAs and antibodies details are available in Table S1. Images were acquired on a fully automated Molecular Devices IXM with a 10×/0.45 NA P-APO objective. The resulting images were analyzed using Cell Profiler software (Carpenter et al., 2006) for quantitative and automated measurements of fluorescence from the antibodies as previously described (Stadler et al., 2012). Briefly, nuclei were segmented in the nuclear channel, the Golgi complex was segmented in the Golgi marker channel, and using the segmented nuclei as seeds, the two structures were associated. Intensity profiles of each compartment were acquired. A reduction of 25% or more of the antibody staining in both Golgi and nuclear compartments was considered as a validation of the antibody specificity (Figs. S1 and S2; and Table S1).

### Golgi-cisternal localization analysis

As previously described (Dejgaard et al., 2007), HeLa-K cells were treated with nocodazole (33 μM) or the solvent control methanol for 3 h. After treatment, cells were fixed with 4% PFA, permeabilized with 0.1% Triton-X100 and immunostained using antibodies against *trans*-Golgi marker, TGN46, *cis*-Golgi marker, GM130, and the protein of interest. Images of the Golgi mini-stacks were acquired using a confocal microscope Olympus Fluoview FV3000 with a 60×/1.3 NA silicon oil immersion apochromatic objective. Golgi mini-stacks were visually inspected, and those that were in the same plane were selected for analysis. Images were analyzed using Fiji (Schindelin et al., 2012); the relative position of the fluorescence profile of the protein of interest against the *trans*- and *cis-Golgi* markers was measured using a plot profile tool. The measured distances were used to calculate PCC between *cis*-Golgi and *trans*-Golgi markers and proteins of interest.

### Drug treatments

Cells were treated 24 h after seeding. For CPT, ETO, and MMC experiments, cells were treated for 16 h at a concentration of 0.1, 50, and 5 μM, respectively (unless indicated otherwise). After treatment cells were incubated with fresh medium for 2 h. H_2_O_2_ experiments were performed by treating cells for 20 min at a concentration of 50 μM, followed by change with fresh medium and 15-min recovery. For nocodazole and KBrO_3_ experiments, cells were treated for 3 h at the concentration of 33 μM and 5 mM, respectively. DOX was used at a concentration of 40 μM for 3 h unless indicated otherwise. For kinase and importin-β inhibitor treatments, cells were pretreated for 30 min with the described inhibitor prior to the addition of DOX. The inhibitors were used at the following concentrations: IPZ (20 μM), KU55933 (ATMi) (30 μM), NU7441 (DNA-PKi) (10 μM), and VE-821 (ATRi) (10 μM).

### Immunofluorescence assay

Cells were fixed with 4% paraformaldehyde in PBS for 15 min and permeabilized with 0.1% Triton X-100 for 15 min at room temperature, then cells were blocked with 5% bovine serum albumin in 0.05% Triton X-100 for 60 min and incubated with primary antibodies in blocking buffer at room temperature for 3 h. Following three washes with PBS, cells were incubated with fluorescent dye-conjugated secondary antibodies diluted in a blocking buffer for 1 h at room temperature.

### Image and data analysis

Confocal microscopy was performed on fixed and immunostained samples using an Olympus FV3000 microscope. Z stacks of images covering the entire cell thickness were acquired. All image analysis was performed using Cell Profiler (Carpenter et al., 2006) and Fiji (Schindelin et al., 2012). Briefly, first nuclei were segmented in the Hoechst channel. When appropriate, the Golgi complex was segmented in the Golgi marker channel, and using the segmented nuclei as seeds, the two structures were associated. Intensity profiles, morphology features, and structure counting analysis were performed when required using Cell Profiler.

### Comet assay

The assay was carried out as previously described (Vodenkova et al., 2020). Briefly, cells were trypsinized, pelletized, and resuspended in ice-cold PBS at a concentration of 25 × 10^4^ cells per ml. The cells were resuspended in 2% low melting agarose (Sigma-Aldrich) and spread quickly onto gel bond film (Biozym) covered in 1% agarose (Sigma-Aldrich). Samples were immersed into a lysis buffer (100 mM EDTA, 2.5 M NaCl, 10 mM Tris-HCl, and 1% Triton-X100; pH 10) overnight at 4°C, followed by a wash with ice-cold water and run in an electrophoresis chamber (alkaline buffer: 1 mM EDTA and 300 mM NaOH; pH 13) at 15 V, 300 mA for 60 min at 4°C. Slides were first washed in a Tris-HCl neutralization buffer (0.4 M; pH 7.5) followed by water, stained with SYBR Gold (Thermo Fisher Scientific) (1:10,000). and finally dried. Comets were imaged by an automated Olympus Scan^R screening microscope, and comet tails were scored using OpenComet plugin (Gyori et al., 2014).

### Western blotting analysis

HeLa-K cells were lysed using radioimmunoprecipitation assay buffer (Thermo Fisher Scientific) with a complete protease inhibitor cocktail (Roche). SDS-PAGE was performed on precast Tris-acetate gels followed by transfer to PVDF transfer membrane (Merck Millipore). Proteins were detected using primary antibodies as described, followed by incubation with secondary antibodies coupled with HRP (Invitrogen). Detection of protein was performed using Pierce ECL Plus Western Blotting Substrate reagent (Thermo Fisher Scientific) and visualized on Azure 280 chemiluminescent imaging system. Golgi enrichment assay was performed using Minute Golgi Apparatus Enrichment Kit (GO-037, Invent Biotechnologies) according to the manufacturer’s instructions. The purity of the *cis* and *trans*-Golgi fractions was confirmed by using Golgi markers GM130 for the *cis*-Golgi and TGN46 for the *trans-*Golgi. Subcellular fractionation was performed using a subcellular protein fractionation kit for cultured cells (78840; Thermo Fisher Scientific) according to the manufacturer’s instructions. Fractions were verified using well-established markers (GM130 for Golgi membranes, Lamin B1 for the nuclear compartment, and α-tubulin for the cytoplasmic fraction) and probed for proteins of interest presence in each compartment. Protein membrane:nuclear distribution ratio was calculated by first dividing the protein level in each fraction by the protein level of the appropriate control; the resulting membrane (M_protein_/M_GM130_) and nuclear (N_protein_/N_laminB1_) ratios were further divided to give the ratio = (M_protein_/M_GM130_)/(N_protein_/N_laminB1_). The ratios obtained from the control were normalized to 1 and compared with the treated group.

### IP

HeLa-K cells were lysed using a lysis buffer (50 mM HEPES, 130 mM NaCl, 1 mM DTT, and 1% NP-40) with a complete protease inhibitor cocktail (Roche). Cell lysates were centrifuged at 16,000 ×g for 10 min at 4°C. For IP, the lysates were incubated with the primary antibody described and rotated overnight at 4°C. Next, the lysates were incubated with Protein G-agarose beads (Roche) and rotated for 4 h at 4°C. The samples were washed with cold lysis buffer and then precipitated proteins were eluted by 2× SDS sample buffer and analyzed by western blotting.

### Colony formation assay

HeLa-K or U-2 OS cells, transfected with either a giantin or control siRNA, were seeded on a 6-well plate (500 cells/well) and allowed to grow for 12 days in a complete culture medium. Where indicated, 24 h after seeding, the cells were treated with a DNA damaging agent, DOX (1 μM), or a solvent control, DMSO for 24 h. Subsequently, the medium was replaced, and it was refreshed every 4 days. The resulting colonies were washed twice with PBS, followed by fixing and staining for 30 min with a solution containing 0.1% (wt/vol) crystal violet and 20% (vol/vol) ethanol. Then the colonies were washed again to remove excess crystal violet with PBS. Finally, after the dishes were dry, digital images of the colonies were acquired using a camera and quantified.

### DR-GFP HR reporter assay

HR efficiency was measured using HEK293T-DR-GFP cells (DSMZ; ACC844), which stably integrate a DR-GFP reporter cassette containing two mutant copies of GFP, one disrupted by an I-SceI restriction site and one truncated, such that I-SceI–induced DSB repair by HR restores a functional GFP open reading frame (Pierce et al., 1999). Cells were transfected with siRNAs targeting giantin, RAD51C, or a nontargeting control at 15 nM using Lipofectamine 2000 for 72 h, followed by transfection of the I-SceI expression plasmid pCBASce (#26477; Addgene) using FuGENE HD (Promega) according to the manufacturer’s instructions for a further 48 h. GFP-positive cells were quantified by automated high-content imaging on an Olympus Scan^R screening microscope and analyzed using CellProfiler software (Carpenter et al., 2006). The percentage of GFP-positive cells per condition was normalized to the nontargeting siRNA control set to 1.

### Statistical analysis

All data were obtained from at least three independent experiments if not otherwise stated. Statistical analyses were performed using a two-tailed unpaired Student’s *t* test for pairwise comparisons and one-way ANOVA with an appropriate post hoc test for multiple comparisons on GraphPad Prism 9. The specific post hoc test applied is indicated in the relevant figure legend. Data are expressed as the standard error of the mean (SEM). *n* values indicate biologically independent samples and experiments. P < 0.05 was considered statistically significant.

### Online supplemental material

Fig. S1 shows validation of DDR protein antibody specificity and the dual Golgi–nuclear localization of the identified proteins by siRNA depletion. Fig. S2 shows additional antibody and localization validation, the experimentally derived protein–protein interaction networks of the validated dual-localizing proteins, and their Golgi-cisternal distribution quantified by Pearson’s correlation analysis. Fig. S3 shows the DOX-induced redistribution of DDR proteins between the Golgi and the nucleus. Fig. S4 shows the H_2_O_2_-induced redistribution of DDR proteins between the Golgi and the nucleus. Fig. S5 shows the KBrO_3_-induced redistribution of DDR proteins between the Golgi and the nucleus. Fig. S6 shows validation of RAD51C antibody specificity and subcellular fractionation, together with RAD51C population redistribution following IPZ treatment and inhibition of ATM, ATR, and DNA-PK signaling. Fig. S7 shows RAD51C redistribution in response to additional DNA-damaging agents (CPT, ETO, and MMC) and the impact of giantin and other golgins on HR repair, cell cycle progression, and patient survival. Table S1 lists the siRNA-mediated validation of the HPA antibody candidates and the functional annotations of the dual-localizing proteins. Table S2 lists the PCCs of the dual-localizing DDR proteins with the *cis*-Golgi marker GM130 and the *trans*-Golgi marker TGN46. Table S3 summarizes the pan-cancer overall survival analysis of *GOLGB1* (giantin) expression across TCGA cohorts.

## Supplementary Material

Review History

Table S1 shows siRNA-mediated validation of HPA antibody candidates and functional annotations of dual-localizing proteins.

Table S2 shows PCCs of dual-localizing DDR proteins with the *cis*-Golgi marker GM130 and the *trans*-Golgi marker TGN46.

Table S3 shows pan-cancer overall survival analysis of GOLGB1 (giantin) expression across TCGA cohorts.

SourceData F3 is the source file for Fig. 3.

SourceData F4 is the source file for Fig. 4.

SourceData F5 is the source file for Fig. 5.

SourceData FS6 is the source file for Fig. S6.

## Data Availability

The data are available from the corresponding author upon reasonable request.
