# Supplementary figures and images for: Spatiotemporal regulation of DNA repair proteins between Golgi and nucleus maintains genome stability
Source: J Cell Biol. 2026 Jul 28;225(9):e202605024. doi: 10.1083/jcb.202605024 (PMC13411647; doi:10.1083/jcb.202605024)

Figure 3C

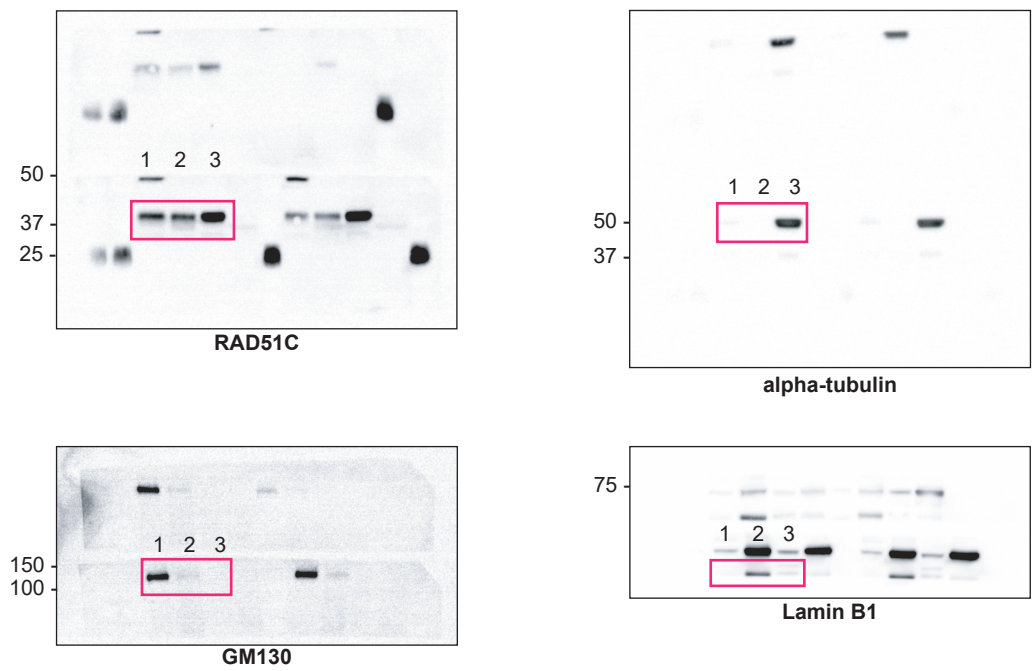

Supplement: SourceData F3 — is the source file for Fig. 3. [file jcb_202605024_sourcedataf3.pdf]
