# Supplementary material for: Spatiotemporal regulation of DNA repair proteins between Golgi and nucleus maintains genome stability
Source: J Cell Biol. 2026 Jul 28;225(9):e202605024. doi: 10.1083/jcb.202605024 (PMC13411647; doi:10.1083/jcb.202605024)
Supplement: SourceData F4 — is the source file for Fig. 4. [file jcb_202605024_sourcedataf4.pdf]

Figure 4H

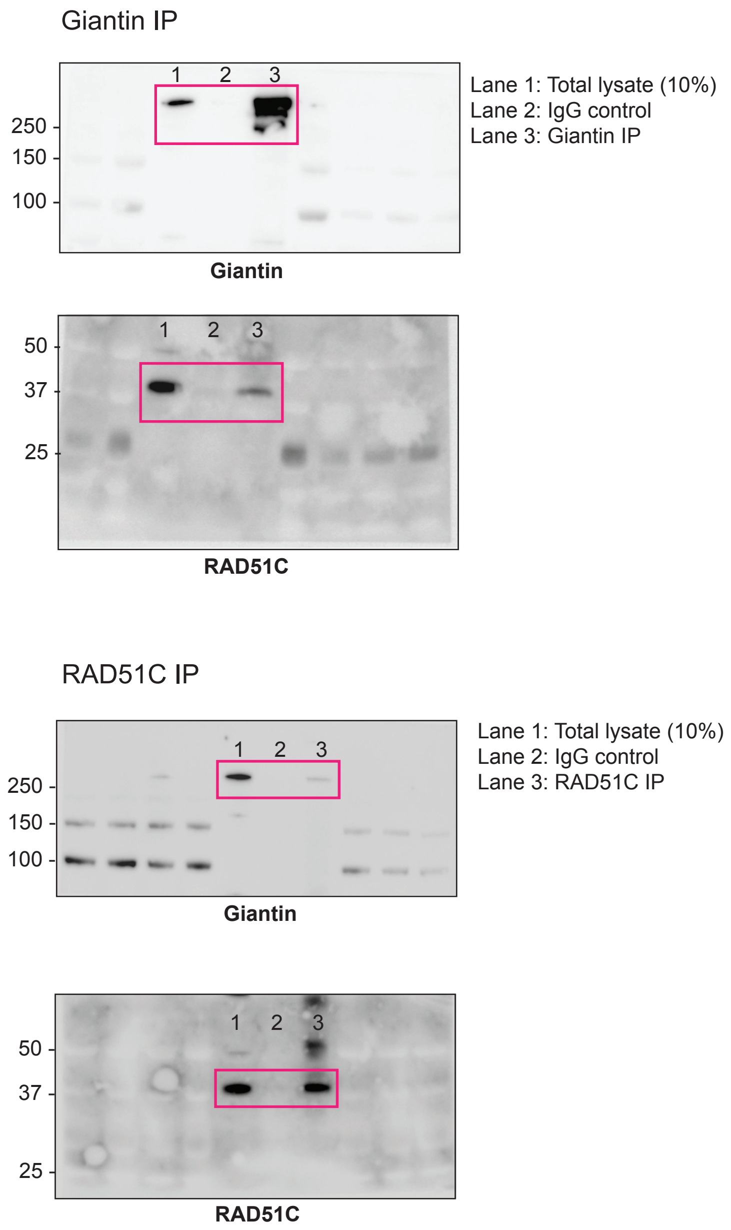
Giantin IP
1 2 3
250
150
100
Lane 1: Total lysate (10%)
Lane 2: IgG control
Lane 3: Giantin IP
Giantin
1 2 3
50
37
25
RAD51C
RAD51C IP
1 2 3
250
150
100
Lane 1: Total lysate (10%)
Lane 2: IgG control
Lane 3: RAD51C IP
Giantin
1 2 3
50
37
25
RAD51C

Figure 4I

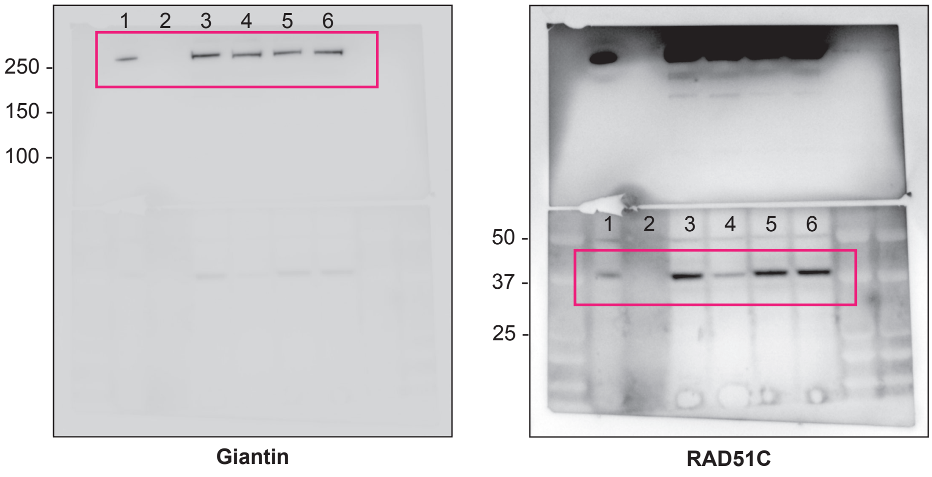

Lane 1: Total lysate (10%)
Lane 2: IgG control
Lane 3: Giantin IP + no additional treatment
Lane 4: Giantin IP + doxorubicin treatment
Lane 5: Giantin IP + doxorubicin + ATM inhibitor treatment
Lane 6: Giantin IP + doxorubicin + IPZ treatment
