# Supplementary material for: Spatiotemporal regulation of DNA repair proteins between Golgi and nucleus maintains genome stability
Source: J Cell Biol. 2026 Jul 28;225(9):e202605024. doi: 10.1083/jcb.202605024 (PMC13411647; doi:10.1083/jcb.202605024)
Supplement: SourceData F5 — is the source file for Fig. 5. [file jcb_202605024_sourcedataf5.pdf]

Figure 5J

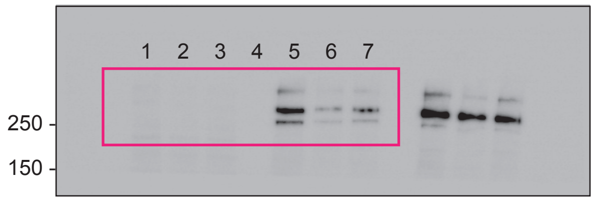

pATM

Lane 1: Control siRNA; DMSO control
Lane 2: Giantin siRNA seq 1; DMSO control
Lane 3: Giantin siRNA seq 2; DMSO control
Lane 4: Ladder
Lane 5: Control siRNA; doxorubicin treatment
Lane 6: Giantin siRNA seq 1; doxorubicin treatment
Lane 7: Giantin siRNA seq 2; doxorubicin treatment

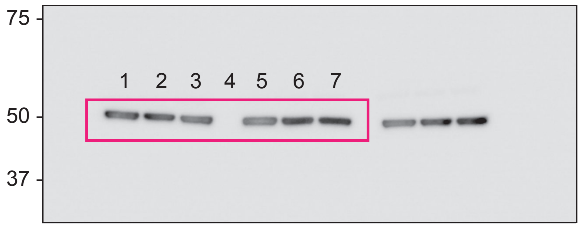

alpha-tubulin

Figure 5K

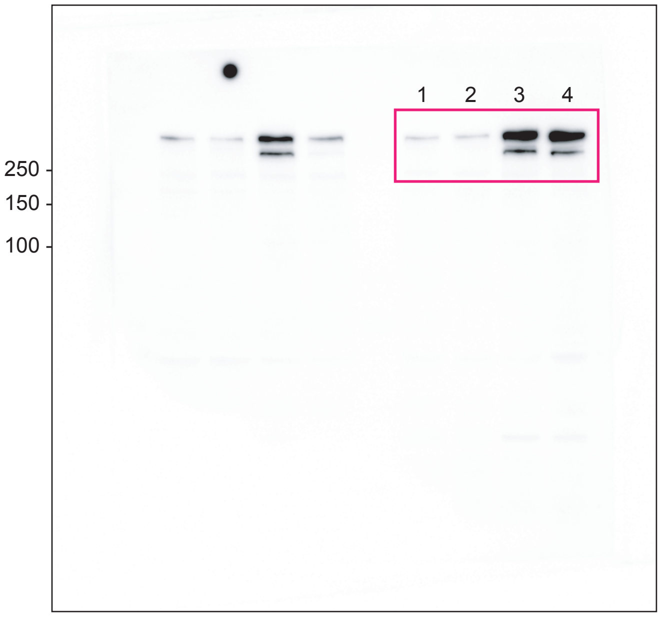

p-ATM

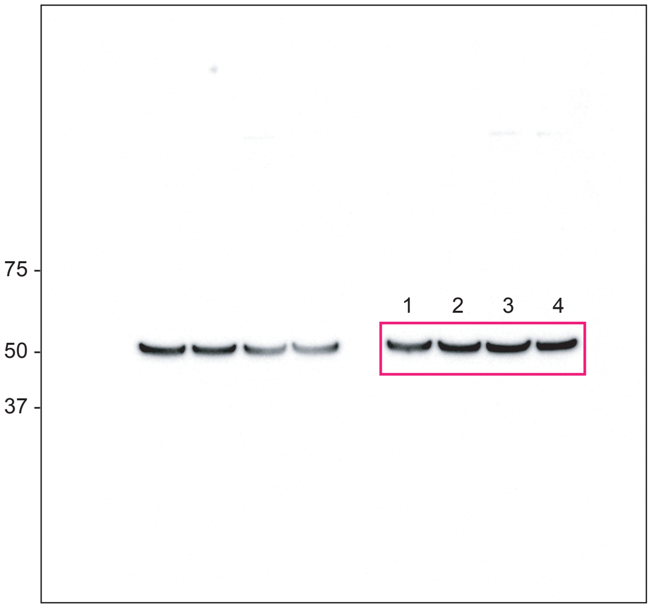

alpha-tubulin

Lane 1: DMSO control
Lane 2: DMSO + IPZ treatment
Lane 3: Doxorubicin treatment
Lane 4: Doxorubicin treatment + IPZ treatment

Figure 5M

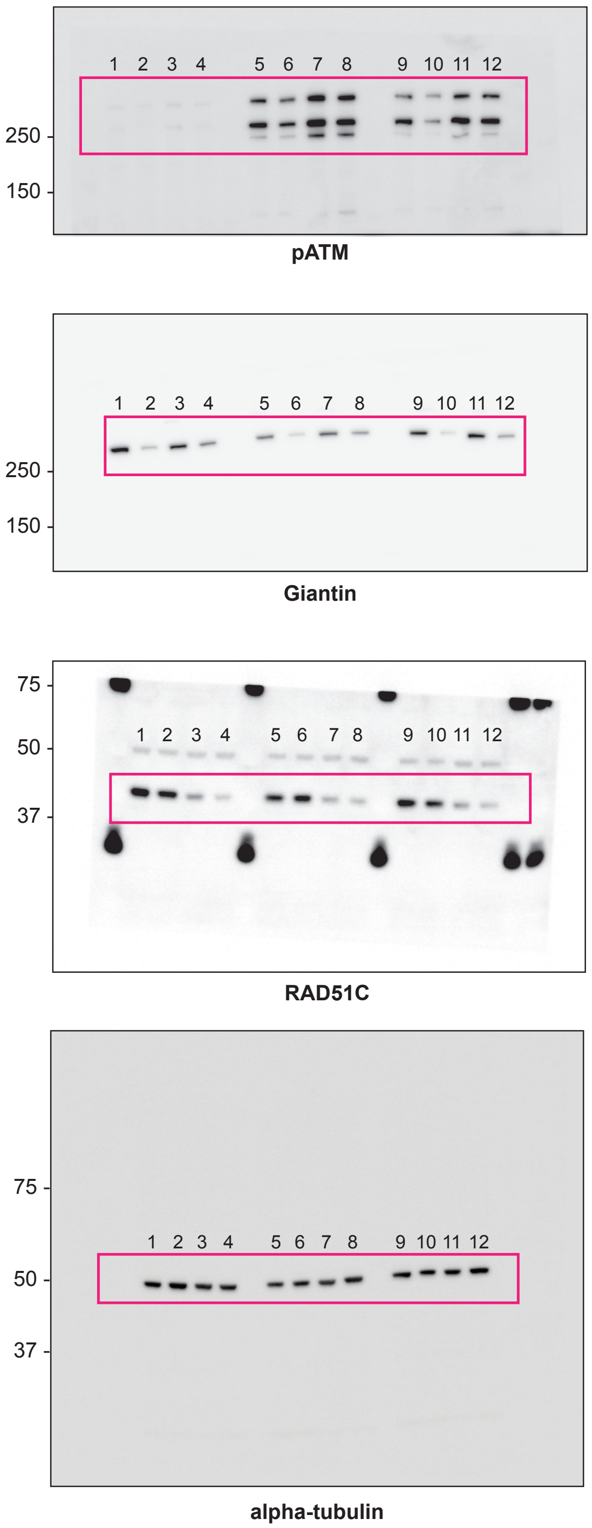

Lane 1: Control siRNA; DMSO control
Lane 2: Giantin siRNA seq 1; DMSO control
Lane 3: RAD51C siRNA seq 1; DMSO control
Lane 4: Giantin + RAD51C seq 1; DMSO control
Lane 5: Control siRNA; doxorubicin treatment
Lane 6: Giantin siRNA seq 1; doxorubicin treatment
Lane 7: RAD51C siRNA seq 1; doxorubicin treatment
Lane 8: Giantin + RAD51C seq 1; doxorubicin treatment
Lane 9: Control siRNA; etoposide treatment
Lane 10: Giantin siRNA seq 1; etoposide treatment
Lane 11: RAD51C siRNA seq 1; etoposide treatment
Lane 12: Giantin + RAD51C seq 1; etoposide treatment
