# Supplementary material for: Spatiotemporal regulation of DNA repair proteins between Golgi and nucleus maintains genome stability
Source: J Cell Biol. 2026 Jul 28;225(9):e202605024. doi: 10.1083/jcb.202605024 (PMC13411647; doi:10.1083/jcb.202605024)
Supplement: SourceData FS6 — is the source file for Fig. S6. [file jcb_202605024_sourcedatafs6.pdf]

Supplementary Figure 6c

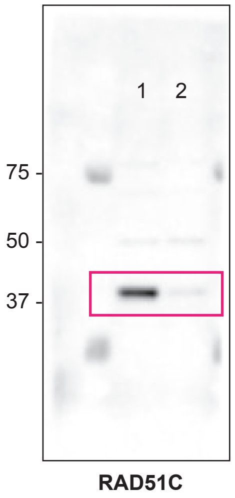

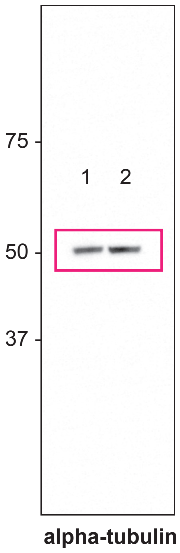

Lane 1: Control siRNA
Lane 2: RAD51C siRNA seq 1

Supplementary Figure 6D

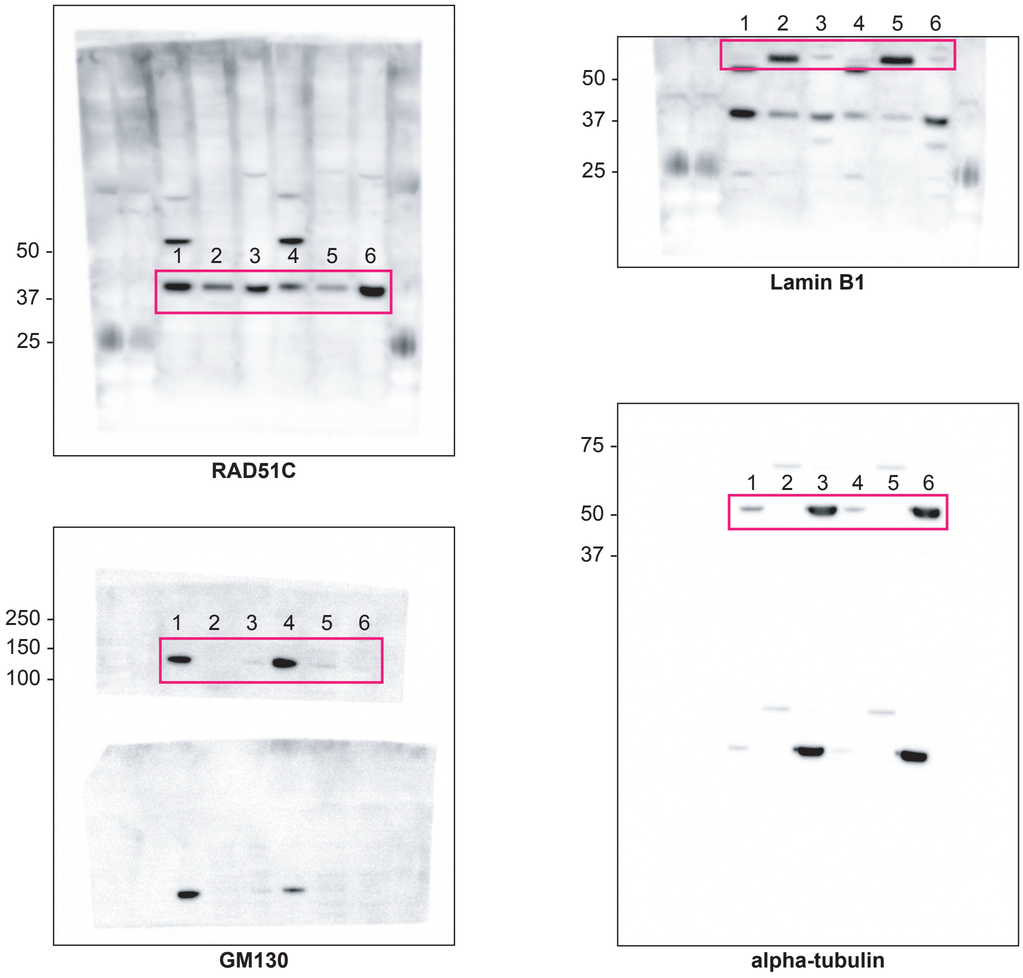

Lane 1: Membrane fraction; DMSO control
Lane 2: Nuclear fraction; DMSO control
Lane 3: Cytoplasmic fraction; DMSO control
Lane 4: Membrane fraction; doxorubicin treatment
Lane 5: Nuclear fraction; doxorubicin treatment
Lane 6: Cytoplasmic fraction; doxorubicin treatment
