# Supplementary material for: Spatiotemporal regulation of DNA repair proteins between Golgi and nucleus maintains genome stability
Source: J Cell Biol. 2026 Jul 28;225(9):e202605024. doi: 10.1083/jcb.202605024 (PMC13411647; doi:10.1083/jcb.202605024)
Supplement: Review History [file jcb_202605024_review_history.pdf]

Spatiotemporal regulation of DNA repair proteins between Golgi and nucleus maintain genome stability

George Galea, Karolina Kuodyte, Muzamil Khan, Peter Thul, Beate Neumann, Emma Lundberg, and Rainer Pepperkok

Corresponding Author(s): George Galea, European Molecular Biology Laboratory

Review Timeline:

Submission Date:	2026-05-07
Editorial Decision:	2026-05-27
Revision Received:	2026-06-11

Monitoring Editor: Roger Greenberg

Scientific Editor: Andrea Marat

Transaction Report:

(Note: With the exception of the correction of typographical or spelling errors that could be a source of ambiguity, letters and reports are not edited. The original formatting of letters and referee reports may not be reflected in this compilation.)

DOI: https://doi.org/10.1083/jcb.202605024

Revision 0

Review #1

1. Evidence, reproducibility and clarity:

Evidence, reproducibility and clarity (Required)

This paper describes the localisation of DNA repair proteins, which carry out their DNA repair function in the nucleus, to the cytoplasmic Golgi apparatus. Using the Human Protein Atlas to identify candidates, the authors use antibody localisation to show that a significant number of DNA repair proteins also localise at the Golgi. It appears that proteins involved in common DNA repair pathways localise to common regions of the Golgi. The Golgi-nucleus distribution of the DNA repairs proteins changes upon DNA damage, indicating a dynamic relationship. The authors focus on the DNA repair protein RAD51C and show that its loss from the Golgi and translocation to the nucleus upon DNA damage is mediated by the ATM kinase. Anchoring at the Golgi is shown to be mediated by the golgin giantin. A functional role for giantin in DNA repair is shown in knockdown studies, supporting a mechanism whereby Golgi anchoring of RAD51C, and possibly other DNA repair proteins, by giantin, is required to maintain proper control of DNA repair.

The data are clear and support the authors' conclusions. The data are carefully quantified throughout. I found the text easy to read.

Major points:

1. To validate the Golgi localisation, KD using siRNA was used. It was deemed that a signal reduction of 25% was enough to indicate specific antibody labelling. This seems like a low number, and not very stringent. For some of the hits, expressing tagged versions of the proteins would greatly strengthen the Golgi assignment. This may not be possible for all, but for RAD51C would seem an important experiment.
2. The total signal should be quantified for each DNA repair protein upon genotoxic stress, in addition to the Golgi to nucleus ratio. For many of the proteins it looks like the total signal goes down, which could influence interpretation.
3. The study would benefit from live imaging of the Golgi to nucleus translocation of RAD51C. This would give a better indication of dynamics.
4. The double depletion experiments suggest a functional relationship between giantin and RAD51C. But they do not formally show it. Experiments to more directly address the functional role of the interaction between these two proteins would strengthen the study.
5. The Kaplan-Meier plots in Fig S9 seems to be quite selective in that only breast cancer is

shown. Does giantin reduction correlate with poor prognosis in other cancers?

Minor points: There are a few grammatical errors here and there. The figures do not appear in the correct order in the text, which makes the early parts of the paper a bit difficult to follow. Some of the figures don't seem to clearly match the text. For example, it is mentioned that RAD51C labelling was done with 3 different antibodies. I could not find this data.

2. Significance:

Significance (Required)

This paper is novel and should be of significant interest to the field. It has important implications for how we think about the Golgi apparatus, and for how DNA repair pathways may be controlled. The pattern is clearly complex, with many DNA repair proteins localising to the Golgi, and some showing opposite dynamics. However, by focussing on RAD51C and giantin, the paper nicely demonstrates a novel mechanism for controlling DNA repair by these proteins.

3. How much time do you estimate the authors will need to complete the suggested revisions:

Estimated time to Complete Revisions (Required)

(Decision Recommendation)

Between 1 and 3 months

4. *Review Commons* values the work of reviewers and encourages them to get credit for their work. Select 'Yes' below to register your reviewing activity at [Web of Science Reviewer Recognition Service](#) (formerly Publons); note that the content of your review will not be visible on Web of Science.

Web of Science Reviewer Recognition

No

Review #2

1. Evidence, reproducibility and clarity:

Evidence, reproducibility and clarity (Required)

Background - Eukaryotic cells rely on tightly regulated DNA repair pathways to preserve genome stability under the constant threat of both endogenous and exogenous genotoxic stress. While the nucleus, and to a lesser extent the mitochondria, is the primary site where DNA damage is detected and repaired, accumulating evidence indicates that extranuclear organelles, particularly the Golgi apparatus, play a surprisingly important role in modulating stress signaling, proteostasis, and the trafficking/activation of key DNA repair factors.

Emerging evidence has shown that genotoxic stress can result in a major remodeling of the Golgi apparatus; however, the crosstalk between the Golgi and the nucleus, and its contribution to the DNA damage response, remains poorly defined. The present study offers timely insight by examining the spatiotemporal behavior of DNA repair proteins that shuttle between the Golgi and the nucleus, and how this trafficking contributes to the maintenance of genomic stability.

Main findings - The authors employed the Human Protein Atlas (HPA) project to shortlist proteins that might link Golgi-nuclear function and validated each candidate using an siRNA-mediated antibody-validation pipeline, thereby identifying 163 proteins that localize to both the Golgi and the nucleus. Bioinformatic analysis of these candidates revealed a significant enrichment for DNA damage response (DDR) regulators, including multiple factors from core DNA repair pathways, suggesting that a portion of the DDR machinery may reside in the Golgi at steady state. Interestingly, the authors observed that dual-localizing DDR proteins undergo lesion-specific redistribution between the Golgi and the nucleus in response to specific types of DNA injuries. For instance, BER and MMEJ proteins shifted from nucleus to Golgi in response to doxorubicin, whereas MMR and HR proteins redistributed from Golgi to nucleus. This trend was reversed with H2O2 or KBrO3 treatments.

To gain further insight into the link between the DDR and Golgi-nuclear communication, the authors focused on the HR factor RAD51C, which also plays a key role during the replicative stress response. The authors noticed that RAD51 is significantly associated with the Golgi, in addition to its known nuclear pool. Interestingly, they demonstrated that doxorubicin triggers the ATM-dependent release of this Golgi-tethered RAD51C pool and its Importin-β-mediated import into the nucleus, where it forms repair-associated foci. They further identified Giantin as the Golgi scaffold that anchors RAD51C at steady state in this subcellular compartment and showed that its depletion leads to premature nuclear accumulation of RAD51C, formation of aberrant RAD51C foci lacking canonical HR markers, reduced ATM activation, elevated genomic instability, and increased cell

proliferation.

Together, this study revealed an underappreciated and functionally meaningful spatiotemporal level of regulation within the DDR, suggesting that the Golgi, rather than functioning solely as a trafficking organelle, acts as a platform that anchors, releases, and temporally controls the availability of key DNA repair factors in response to genotoxic stress. In particular, the authors demonstrated that the timely and regulated release of RAD51C from the Golgi is essential for maintaining genome stability and is dependent on canonical DDR signaling pathways, including ATM activation and Importin-β-mediated nuclear import.

Overall Critique - This manuscript offers a novel and compelling perspective on the regulation of the DDR by positioning the Golgi as an active participant in the spatiotemporal control of DNA repair factors. By integrating multiple experimental layers, including a systematic localization screening, a sub-Golgi mapping, several dynamic redistribution assays, and functional perturbation read-outs, the authors built a strong and coherent case for a biologically meaningful Golgi-nucleus communication axis during the DDR. Therefore, the study is timely and highly relevant for the DNA repair field, with broader implications for our understanding of how subcellular organelles coordinate genome maintenance and cellular homeostasis.

While the manuscript is clearly written and the figures are coherent and supportive of the main findings of the study, several issues should be addressed to ensure full interpretability and reproducibility.

Major Comments

1. Limited use of agents causing genotoxic stress - The authors report intriguing lesion-specific shifts in Golgi-nuclear redistribution, yet much of the mechanistic work relies heavily on doxorubicin, a pleiotropic drug that induces diverse forms of DNA damage beyond DSBs. Expanding the core analysis of the study to include a broader panel of mechanistically defined genotoxins (e.g., etoposide, camptothecin, neocarzinostatin, or ionizing radiation) would substantially strengthen the conclusion that the trafficking patterns reflect damage-type specificity rather than drug-specific off-target effects. Such broader analysis would also clarify whether Golgi-nucleus communication responds differentially to replication-associated breaks, Topo II-dependent lesions, oxidative stress, or crosslinks.
2. Functional implications of RAD51C redistribution for HR efficiency - Although the study

convincingly demonstrates a release of RAD51C from the Golgi and its subsequent nuclear foci formation, it remains unclear how this redistribution influences HR efficiency. Incorporating a functional HR assay (e.g., DR-GFP reporter, RAD51 filament assembly, or fork protection assays) would help determine whether Golgi-anchored RAD51C release is directly required for HR or instead primarily modulates upstream DDR signaling.

In addition, the manuscript does not fully reconcile how Golgi-tethering of RAD51C fits with its well-established nuclear roles during replication stress, where timely availability of RAD51C is essential for fork stabilization and restart.
3. Specificity of Giantin-related phenotypes - The phenotypes observed upon Giantin depletion (e.g., increased micronuclei, comet tail moments, impaired ATM signaling, and elevated proliferation) could partially reflect a global dysfunction of the Golgi rather than RAD51C-specific tethering defects. Although co-depletion of RAD51C provides partial rescue, additional controls examining Golgi integrity, trafficking competence, or rescue with siRNA-resistant Giantin would help confirm specificity and distinguish direct from indirect effects.
4. Positioning of ATM in the Golgi-nuclear signaling - While ATM inhibition prevents RAD51C release, its spatial and mechanistic basis of this regulation remains obscure. It is not clear whether ATM acts locally at the Golgi, through cytoplasmic pools, or indirectly via nuclear feedback signaling. Clarifying or discussing this point in more depth would improve the mechanistic coherence of the proposed model.
5. RAD51C is examined in silo, without consideration for the BCDX2 complex - RAD51C is exclusively analyzed in isolation, despite its well-established function as part of the BCDX2 paralog complex (RAD51B-RAD51C-RAD51D-XRCC2). Because RAD51C does not normally operate as a standalone factor, it is unclear why only RAD51C, among all paralogs, would be subjected to Golgi tethering, ATM-dependent release, and Importin-β-driven nuclear import. This raises important mechanistic questions: Are other BCDX2 members also Golgi-associated? Do they undergo similar trafficking dynamics? Does Golgi tethering selectively regulate RAD51C, or does the complex translocate together? Addressing these points would greatly strengthen the biological plausibility and mechanistic coherence of the proposed model.

Minor Comments

1. Pathway-specific sub-Golgi localization patterns - The finding that DDR proteins map to distinct cis/trans Golgi subdomains is an interesting and potentially important observation. However, the dataset is limited to 15 proteins, making the proposed pathway-level trends (e.g., HR factors enriched in cis-Golgi; BER/MMEJ factors enriched in trans-Golgi)

preliminary. Strengthening this conclusion by increasing the number of DDR proteins analyzed would help determine whether sub-Golgi compartmentalization contributes meaningfully to DNA repair pathway regulation.
2. Is the Golgi-released RAD51C indeed the pool that enters the nucleus? The major assumption of the study is that the RAD51C population released from the Golgi upon DNA damage is the same pool that subsequently accumulates in the nucleus to form repair foci. While the imaging and fractionation data are consistent with this model, the study does not directly track or distinguish Golgi-derived RAD51C from cytoplasmic or pre-existing nuclear pools. Without a method to specifically label, pulse-chase, or track the Golgi-anchored fraction, it remains formally possible that nuclear RAD51C originates from other subcellular reservoirs.

2. Significance:

Significance (Required)

General assessment - This study presents a novel and conceptually compelling view of the DNA damage response (DDR) by positioning the Golgi apparatus as an active regulator of the spatiotemporal availability of DNA repair factors. The strongest aspects of the work include its integration of a systematic immune-localization screening, a sub-Golgi compartment mapping, dynamic redistribution assays, and functional perturbations to build a coherent model of Golgi-nucleus communication during genotoxic stress. The mechanistic focus on RAD51C provides a clear case study linking organelle-level regulation to genome stability.

Advance - To my knowledge, this is the first comprehensive demonstration that the Golgi can serve as a spatiotemporal coordination node for DDR proteins, including those involved in HR. The identification of a substantial pool of RAD51C, and reportedly other DDR factors, anchored within specific Golgi subdomains represents a significant conceptual advance. The demonstration that Golgi-tethered RAD51C is released in an ATM-dependent manner and subsequently participates in nuclear foci formation suggests a previously unrecognized organelle-level regulatory checkpoint in genome maintenance. This work therefore extends current models of the DDR by revealing a layer of intracellular coordination that bridges classical nuclear pathways with cytoplasmic organelle function.

Audience - This study will be of strong interest to a specialized audience in the fields of DNA repair, genome stability, and cell biology, particularly those studying the spatial organization of repair pathways and intracellular stress signaling. It will also appeal to researchers investigating organelle biology, intracellular trafficking, and the broader

coordination of cytoplasmic and nuclear responses to stress. Beyond these communities, the work may be relevant to cancer, as it suggests new mechanisms by which organelle perturbations or Golgi-associated scaffolding proteins could influence therapeutic responses or genomic instability.

Reviewer expertise - Field of expertise: DNA repair, genome stability, organelle biology, cancer cell biology.

3. How much time do you estimate the authors will need to complete the suggested revisions:

Estimated time to Complete Revisions (Required)

(Decision Recommendation)

Between 1 and 3 months

4. *Review Commons* values the work of reviewers and encourages them to get credit for their work. Select 'Yes' below to register your reviewing activity at Web of Science Reviewer Recognition Service (formerly Publons); note that the content of your review will not be visible on Web of Science.

Web of Science Reviewer Recognition

No

Review #3

1. Evidence, reproducibility and clarity:

Evidence, reproducibility and clarity (Required)

This study investigates the communication between the Golgi complex and the nucleus of the cell, which remains a largely unexplored field. The authors used publicly available siRNA and antibody data from the Human Protein Atlas as a basis for finding overlap between the proteomes of the two cellular compartments. In validating the data from the HPA, the study finds a novel cluster of DNA repair proteins present in the Golgi, which they validate and resolve to sub-compartmental localization. To do so they use immunofluorescence (IF) localization on ¬cis- and trans-Golgi cisternae marked by GM130 and TGN46, respectively. The authors find that many of the fully validated proteins present in both the nucleus and Golgi redistribute between the Golgi and the nucleus dependent on the protein and the type of DNA lesion. They focused on RAD51C, a recombination

factor. They show that RAD51C resides in both the ¬cis- and trans- subsections prior to damage and responds to DNA damage in an ATM-dependent manner via release of a Golgi-based pool bound to Giantin, which is then imported into the nucleus via Importin-β. Knockdown experiments showed that Giantin regulates RAD51C spatially and temporally. The work reveals a dynamic interchange of proteins between the Golgi and nucleus that controls cell functions beyond the classic secretory, membrane trafficking, and PTM roles of the Golgi. The authors build on prior work on Golgi impacts on DDR, offering an alternative cellular compartment for storage of DDR factors prior to damage. Overall, the data is timely and relevant, as it finds new roles for the Golgi in DNA damage response (DDR) regulation. The data is largely convincing and well controlled. The IF data is presented in black and white single channels and merged in color, which allows good comparison of the different protein stains. The scope of the initial screen of HPA antibodies and Golgi/Nuclear dual proteomes is impressive, and the overlap of DDR proteins is characterized for fifteen different proteins at a sub-compartmental level. The focus on RAD51C as a member of the HR pathway was a strong choice, and the study presents interesting information on its regulation by Golgi complex members, as well as a feedback look with pATM. The possibility of the Golgi storing specific DDR factors in specific compartments is well-supported and intriguing. There are a few major and minor points that should strengthen the paper and improve clarity prior to publication.

Major Comments:

1. Much of the strength of the IF data is lost in the choice of scale for presentation of the data. In almost all cases, enlarged sections should be shown of the areas currently indicated by arrow, in all channels. This is done well in Figure 3A, where an area of the Golgi is enlarged and the overlap of RAD51C in the GM130-marked Golgi is clearly visible in the merged channel, even when printed out. I would highly recommend including the white box and enlarged in all images and channels, while keeping the representative fields as is (e.g. if the image is 40mm, draw a 7mm box around representative cells/Golgi, and enlarge to 15mm in the bottom left). This change should be made to F1E, F2F, F3E, F3J, and F3M, as well as having enlarged figures in the corners in all supplementary data IF figures. Where possible, a fully enlarged image of the bounding box could also be included. Some of the IF data would be strengthened by using the nuclei stain to draw a masking outline to include in the black and white channels, to clearly delaminate what is Golgi-localized and what is nuclear.
2. There is a lack of consistency in the representative images shown by IF. For example, Figure 1 gives the impression of very little RAD51C in the nucleus but this is rightly shown to not be the case in Supp. Fig 2A. The same is true of the various images of LIG1. The authors

should use representative data that better reflects the distribution of the proteins being studied and maintain consistency across images. If there is a lot of variation in staining patterns, the authors should show images and percentages corresponding to the variations especially for the key gene studied, RAD51C.
3. The initial screening by siRNA-mediated knockdown pipeline that validated and confirmed dual Golgi and nuclear localization of 163 of the 329 dual-localization HPA proteins does not have any data included. This seems like a very large amount of data to gloss over and not include even as supplementary data. This should be included as source data, and discussion of the in-text information should be strengthened. The data included with the networking of these validated proteins is strong, but the process of elimination and validation has not been shown. In addition, the antibody information included in the supplementary data does not include dilution factors or blocking factors is not included, which would be beneficial to future studies to include.
4. The authors should expand upon the paragraph lines 155-162 to include more discussion on Figure S2A and S2B. The expanse of this data is some of the strongest in the paper, and it should be further discussed in-text. Also, the rationale behind the choice in the specific proteins that are included in these analysis / figures is not always clear in -text, and more attention should be spent on the narrowing down of the analysis to the final proteins. This is also especially important as many of the DDR proteins chosen are not the most common DDR proteins. Also note in text that the Golgi marker GM130 (presumably) was used for the screening, which means that some proteins which are only localizing to the TGN46 trans Golgi might have been lost in the validation step (or, explain why this is not the case).
5. The relationship between Giantin loss, increased cell proliferation, and elevated endogenous DNA damage as it relates to RAD51C remains insufficiently resolved and requires further clarification. Several of the proliferation assays used are not optimal for addressing changes in cell growth. For example, Figure 5O appears to quantify cell numbers by counting fields from IF images, which is an unconventional approach. This should be done by growth curves, luminescent viability or colony formation assays. In addition, this point will be greatly strengthened by performing rescue experiments for Giantin directly (instead of co-depletion as a means of rescue) and/or using a mutant of RAD51C that does not bind to Giantin. If these additional experiments are beyond the current scope, the conclusions should be softened in the discussion.
6. It is unclear from the discussion and from presented data whether proteins are directly transported between the Golgi and the nucleus, or whether they go into the cytoplasm for a transient period, presumably when they could interact with Importin β. There is also some data where cytoplasm signal could be quantified to address this (Figure 3E-I).
7. Statistical analysis on experiments with more than two samples need to be performed with ANOVA and a follow up post-hoc test, not with two-tailed unpaired Student's t-test,

which only compares the control and each individual sample. This type of analysis inflates the Type 1 error rates (false positives) in your datasets. For example, the two-tailed unpaired Student's t-test is appropriate in Figure 2F-H, but not in Figure 3 when the samples are timepoints. In this case, a One-way ANOVA with Tukey's post-hoc test (if you want to show all coparisons), or Bonferroni/Sidak if you only need to compare several samples).

Minor Comments:

General

1. Throughout the text, the reference to many figures and supplementary figures in the same sentence, with little discussion of the data therein makes it hard to follow. In-text referencing is particularly confusing in the section "Dual-localising DDR proteins dynamically redistribute between the Golgi and nucleus in response to specific types of DNA injuries," where the reader is switching between multiple figures and supplementary figures.
2. In figures that display technical replicates as individual data points, consider distinguishing each replicate by using different marker shapes (e.g., repeat 1 = upright triangle; repeat 2 = inverted triangle; repeat 3 = diamond). This would provide additional clarity regarding the consistency and repeatability of each technical repeat.
8. Make sure all western blot data includes the marker size (F3C and F5L has none, F4H/I have size of proteins not size of markers).
9. Be consistent with use of capitalization in figure legends and graph/figure labels.

Figure 2

1. In Figure 2A, please include in the figure itself that GM130 is the cis Golgi, and TGN46 is the trans Golgi (Figures should not be dependent on the text for full understanding).
2. Why are LRIG2 and LRRIQ3 not included in the 2E cis vs trans Golgi data, when all other proteins from F1D are included? Include, or comment on in-text.
3. Be sure to include scale bar data in each figure legend (F2A-E is currently missing it), and include updated scales included in the enlarged data.
4. In Figure 2F, make sure that the merged green channel is presented at the same intensity as it is in the single black and white channel, as the green looks very overexposed in several of the merged (CCAR1 DMSO merged is the most noticeable).
5. In Figure 2G, include the grey label in the figure legend.
6. In Figure 2G-H, the method of data presentation in the graphs coupled with the

statistical analysis is confusing and should be expanded upon in the legend.

Figure 3

1. Figure E/F/G: Is there cytoplasmic quantification as well? Your rationale is that the Golgi RAD51C goes into the nucleus, but via the cytoplasm (due to Importin β import); do you see the cytoplasmic levels increase? Or is it too dilute to notice a difference? At least, this omission needs to be mentioned in-text.
2. Figure H/I also include the quantification of the cytoplasmic fraction. It is mentioned in-text on line 272, but not quantified. This comes up as a big question: Do the proteins go directly between the Golgi and nucleus, or do they go through the cytoplasm?
3. Figure 3A, 3E, and if the data is present for 3J and 3M, could all benefit from using the nuclei staining as a mask to draw an outline around the nucleus in the other channels, and then show a merge in full color instead of a nuclei-only channel. Also note from the major comments, that this data especially is so small to see without enlarged images.
4. In-text discussion of the results from Figure 3 has an in-depth discussion of the NLS and NES in RAD51C, but this is not followed up on with site-directed mutagenesis or any data; perhaps move this to the discussion instead of results section.

Figure 4

1. Comments from earlier figures hold, with size of enlarged events and using the nuclei as an outline in the single channels. E.g. Figure 4F arrows appear to point to nothing at the chosen scale. The zoom in 4G is insufficient, as the chosen feature is so small it is not even visible in full fields.
2. Figure 4H and 4I need to show the size of the markers
3. The representative image in 4L for siGiantin pATM has no pATM foci, while the quantification in 4M has a reduction from ~50% to ~25%, so this image is not representative of this data, or the data quantification is not as strong as the actual data.

Figure 5

1. Figure 5A has overexposure of the nuclei stain in order to visualize micronuclei. Readjust the levels, and enlarge the images for better visualization. (is this DAPI-stained? Please label).
2. Figure 5A-C: Figure 5A does not show siRAD51, but it is included in the DMSO only graph. Please either show RAD51 data in 5A and 5C, or do not include in 5B. If the DMSO and ETO experiments were performed separately and that accounts for this discrepancy, then show

separately.
3. Figure 5M the white label is difficult to see in the green box.

Supplementary Figures

1. Consider reordering/ subdividing supplementary figures for ease of reference during reading.
2. SF1 and SF2A: Include enlarged boxes or full images so that data is visible.
3. SF3A, SF4A, and SF5A: Include enlarged images, include nuclei marker if possible (otherwise, the nuclear intensity is not proven nuclear).
4. SF3B-C, SF4B-C, and SF5 B-D: Change the data presentation in the same method as changed for F2G-H.
5. SF3D: List proteins in the same order as in B and C.
6. SF6D: Label M N and C more clearly. Include size labels.
7. SF7A-B: Include enlarged.
8. SF8: Include arrows as in previous experiments, include enlarge.
9. SF9G: G is labelled, but not included.

2. Significance:

Significance (Required)

The work finds new roles for the Golgi in regulation of DNA damage responses and the screen could be an important dataset (but results need to be made available) for the DNA repair community. The scope of the initial screen of HPA antibodies and Golgi/Nuclear dual proteomes is impressive, and the overlap of DDR proteins is characterized for fifteen different proteins at a sub-compartmental level. The work provides important insights into RAD51C regulation, however, there are key mechanistic insights and control experiments missing from the studies involving RAD51C and Giantin, dampening its impact. The idea of an alternative cellular compartment for storage of DDR factors prior to damage is interesting, and suggests the spatial regulation of specific lesion responses are stored in specific sub-compartments of the Golgi, which could contribute to repair regulation.

3. How much time do you estimate the authors will need to complete the suggested revisions:

Estimated time to Complete Revisions (Required)

(Decision Recommendation)

Between 1 and 3 months

4. *Review Commons* values the work of reviewers and encourages them to get credit for their work. Select 'Yes' below to register your reviewing activity at Web of Science Reviewer Recognition Service (formerly Publons); note that the content of your review will not be visible on Web of Science.

Web of Science Reviewer Recognition

Yes

Full Revision

Manuscript number: RC-2025-03297R
Corresponding author(s): Galea George; Pepperkok Rainer

1. General Statements

We are grateful to all three reviewers for the depth and care of their engagement with this manuscript. We were encouraged that all reviewers found the central concept novel and compelling, and their constructive criticism has substantially strengthened the work.

In the revised manuscript we have made the following major additions: a DR-GFP homologous recombination efficiency assay directly linking Giantin to RAD51C-dependent HR capacity; cytoplasmic RAD51C quantification across the doxorubicin time course; a pan-cancer survival analysis of GOLGB1 across all TCGA cohorts with a full hazard ratio summary table; expanded discussion of the ATM-Golgi axis in light of recent literature; and updated statistical analyses throughout with one-way ANOVA replacing multiple pairwise t-tests where appropriate. Source data have been provided for all quantitative datasets as Supplementary Tables.

In addition to the manuscript revisions, the rebuttal letter includes two datasets generated specifically to address reviewer concerns: a VSV-G-YFP trafficking assay confirming that general Golgi secretory function is preserved upon Giantin depletion, and biochemical fractionation data providing evidence for damage-induced ATM recruitment to Golgi fractions. These data are presented within the relevant responses below and are available to the reviewers, but have not been incorporated into the revised manuscript in order to maintain narrative focus.

Detailed point-by-point responses to all three reviewers follow, with all manuscript changes described within the relevant responses.

2. Point-by-point description of the revisions

Reviewer #1 (Evidence, reproducibility and clarity (Required)):
This paper describes the localisation of DNA repair proteins, which carry out their DNA repair function in the nucleus, to the cytoplasmic Golgi apparatus. Using the Human Protein Atlas to identify candidates, the authors use antibody localisation to show that a significant number of DNA repair proteins also localise at the Golgi. It appears that proteins involved in common DNA repair pathways localise to common regions of the Golgi. The Golgi-nucleus distribution of the DNA repairs proteins changes upon DNA damage, indicating a dynamic relationship. The authors focus on the DNA repair protein RAD51C and show that its loss from the Golgi and translocation to the nucleus upon DNA damage is mediated by the ATM kinase. Anchoring at the Golgi is shown to be mediated by the golgin giantin. A functional role for giantin in DNA repair is shown in knockdown studies, supporting a mechanism whereby Golgi anchoring of RAD51C, and possibly other DNA repair proteins, by giantin, is required to maintain proper control of DNA repair. The

data are clear and support the authors' conclusions. The data are carefully quantified throughout. I found the text easy to read.

Major points:

1.) To validate the Golgi localisation, KD using siRNA was used. It was deemed that a signal reduction of 25% was enough to indicate specific antibody labelling. This seems like a low number, and not very stringent. For some of the hits, expressing tagged versions of the proteins would greatly strengthen the Golgi assignment. This may not be possible for all, but for RAD51C would seem an important experiment.

Response: We thank the reviewer for raising the important issue of antibody validation stringency. We agree that for a single-candidate study, a larger reduction after knockdown would generally be preferable. In our case, the 25% cutoff was used only in the primary high-content screening step as part of an intentionally inclusive two-stage workflow, for the following reasons:

First, because this dataset is generated in a screening format across hundreds of targets, knockdown-efficiency, protein turnover, and the relative size of the Golgi associated pool are unknown and highly variable between genes. For many proteins the Golgi pool represents a small fraction of total cellular signal, and a modest change in total abundance can translate into a smaller absolute change in the Golgi ROI after segmentation, background subtraction, and imaging noise. We therefore selected a permissive cutoff to reduce false negatives and ensure we did not systematically miss candidates with slower turnover, partial knockdown, or small Golgi pools. This strategy is consistent with large scale subcellular mapping efforts, including the Human Protein Atlas, where genetic depletion by siRNA is used as a key validation pillar for immunofluorescence localization and is combined with additional validation strategies when deeper confidence is required (Stadler *et al*, 2012). Furthermore, it is important to note that this validation was performed in a high-content screening format in which fixation, permeabilisation, antibody concentration, and blocking conditions were kept uniform across all candidates rather than optimised for each individual antibody. In standard single-target immunofluorescence experiments, these parameters would be titrated to maximise signal-to-noise for the specific antibody and antigen in question. Under non-optimised screening conditions, the absolute magnitude of signal change upon knockdown is inherently attenuated compared to what would be expected from a purpose-optimised assay. We therefore consider a 25% reduction threshold under these uniform, non-optimised screening conditions to be a meaningful and appropriately calibrated criterion.

Second, we wish to clarify that the primary intent of our screen was not to validate the Golgi-nuclear localisation of any single protein in isolation, but rather to identify whether entire functional pathways are represented at the two organelles. This is precisely why the bioinformatic network analysis was performed as an integral part of the workflow, and not as an afterthought. The finding that the validated hit list is significantly enriched for coherent functional clusters, most notably a network spanning multiple core DNA repair pathways (HR, MMR, BER, MMEJ) serves as an *in silico* validation of the dataset as a whole. The emergence of pathway-level organisation, with proteins from the same repair pathways co-associating, localising to the same Golgi sub-

compartments, and redistributing in the same direction upon genotoxic stimuli, provides biological coherence that goes beyond what individual antibody validation can offer, and substantially reduces the likelihood that the Golgi signal represents a collection of unrelated false positives.

Third, our mechanistic conclusions do not rely on the 25% screening threshold. For RAD51C, we used multiple orthogonal validation approaches, including independent antibodies recognizing distinct RAD51C epitopes and genetic depletion, supported by biochemical evidence.

In response to this comment, we have provided the full screening validation dataset as source data (**Supplementary Table S1**), including intensity changes for the candidates, so that readers can inspect the distributions and apply their own thresholds. We have also clarified in the Results section the rationale behind our screening strategy (**lines 128-139**) and the role of the bioinformatic network analysis as an integral validation step (**lines 141-156**).

Turning to the specific suggestion of tagged RAD51C, we fully agree that tagged proteins can provide valuable orthogonal validation. We attempted endogenous tagging using CRISPR-mediated homologous recombination but were unable to obtain viable colonies following editing, consistent with the essential role of RAD51C in homologous recombination. We also attempted ectopic expression of tagged RAD51C but were unable to obtain constructs that preserved physiological expression levels, maintained robust cell viability or produced interpretable localization. This difficulty is not unique to our laboratory: colleagues working on RAD51 paralog complexes have reported that tagging or overexpression of RAD51C perturbs both its localisation and its ability to form functional paralog complexes (Greenhough *et al*, 2023; Rawal *et al*, 2023; Somyajit *et al*, 2015; Berti *et al*, 2020) all use purified complexes or untagged proteins for functional assays. We discussed these challenges extensively with experts in the DNA damage repair field at several international meetings (EMBO Sounio, Keystone Symposia, German DNA Repair Society). For these reasons, we relied on orthogonal approaches that do not require tagging (genetic depletion plus independent antibodies, and biochemical fractionation) to support the Golgi localization claim. We agree with the reviewer that this represents a limitation of this study, and we addressed these concerns in the discussion of our revised manuscript (**lines 630-641**).

2.) The total signal should be quantified for each DNA repair protein upon genotoxic stress, in addition to the Golgi to nucleus ratio. For many of the proteins it looks like the total signal goes down, which could influence interpretation.

Response: We thank the reviewer for this important point. We wish to clarify that our imaging pipeline uses marker-based segmentation throughout, the Golgi compartment is segmented using GM130 and the nucleus using Hoechst, as unsegmented whole-cell masks without organelle markers yield unreliable intensity measurements in this experimental setup. True total cellular signal is therefore not directly accessible in this dataset. In the revised manuscript we provide the absolute fluorescence intensities for both the Golgi and nuclear compartments separately. In addition, we now include total (Golgi + nuclear) intensity measurements for each protein (**Supplementary Figures 3D, 4D,** and **5E**) as the most reliable proxy for overall protein

distribution. These data are presented alongside the redistribution ratio to enable comprehensive interpretation.

As the reviewer correctly notes, a subset of proteins shows a reduction in total signal after treatment, particularly with doxorubicin. This is consistent with known effects of doxorubicin-induced DNA damage on cellular proteostasis, including widespread ubiquitination and suppression of protein translation (Halim *et al*, 2018). Several DDR regulators are subject to ubiquitin-dependent turnover following genotoxic stress, such as CHK1 (Zhang *et al*, 2005). More broadly, ubiquitin and proteasome mediated regulation is an integral component of the DNA damage response and can affect the abundance and detectability of DDR factors (Brinkmann *et al*, 2015). Changes in abundance are therefore an expected biological feature of the response. For this reason, we used the Golgi-to-nucleus ratio as the primary redistribution readout, as it captures relative compartmental partitioning independently of changes in total protein levels.

3.) The study would benefit from live imaging of the Golgi to nucleus translocation of RAD51C. This would give a better indication of dynamics.

Response: We agree that live imaging would directly visualize the dynamics of RAD51C redistribution between the Golgi and the nucleus. This was indeed one of our initial goals following the identification of the Golgi-associated RAD51C pool. However, as described above in our response to Major Comment 1, live imaging requires a fluorescently tagged RAD51C construct, and all tagging strategies we attempted, both endogenous CRISPR-mediated tagging and ectopic expression, failed to yield cell lines with robust signal while preserving physiological behaviour. This appears to be a broader challenge for highly conserved and functionally constrained DNA repair proteins, and is not unique to our laboratory.

Given these constraints, we focused on tag-independent approaches: multiple independent RAD51C antibodies combined with genetic depletion controls, quantitative fixed-cell time courses, and biochemical fractionation. These orthogonal datasets together support compartment-specific changes over time in a manner consistent with redistribution. We have clarified this limitation explicitly in the manuscript and avoided any wording that could be interpreted as implying direct single-molecule tracking in live cells. We present this as an important avenue for future work, contingent on the development of viable RAD51C-expressing cell lines (**lines 630-641**).

4.) The double depletion experiments suggest a functional relationship between giantin and RAD51C. But they do not formally show it. Experiments to more directly address the functional role of the interaction between these two proteins would strengthen the study.

Response: We agree with the reviewer that double depletion alone cannot formally prove that the physical Giantin–RAD51C interaction is the sole determinant of the observed DDR phenotypes. However, we would like to highlight the breadth of evidence we have assembled in support of this functional relationship:

- Physical interaction between endogenous Giantin and RAD51C demonstrated by colocalisation (**Figure 4F-G**) and co-immunoprecipitation (**Figure 4H-I**).

- Damage-induced dissociation of the Giantin–RAD51C complex that is prevented by ATM inhibition or Importazole treatment, directly linking the interaction to the DDR signalling axis (**Figure 3K-P**)
- Premature nuclear accumulation of RAD51C upon Giantin depletion, producing aberrant nuclear foci lacking canonical HR markers and impaired ATM signalling (**Figure 4B-E & J-M**)
- DR-GFP reporter assay confirming that Giantin depletion reduces HR efficiency to approximately 60% of control, consistent with the reduction previously reported in the genome-wide HR screen (Adamson et al. 2012) and validating the functional significance of Giantin in HR (**Figure 5L**).
- Partial rescue of ATM phosphorylation, genomic instability and proliferation phenotypes by RAD51C co-depletion, arguing for RAD51C as a functionally relevant conduit of the Giantin-dependent phenotype (**Figures 5M–5P**).

These observations are further supported by the established literature on RAD51C function, its roles in CHK2 phosphorylation, replication fork stabilisation, and RAD51 filament formation (Badie *et al*, 2009; Somyajit *et al*, 2015; Prakash *et al*, 2022) providing a mechanistically coherent framework in which mislocalisation of RAD51C, whether directly or indirectly through Giantin, leads to dysregulation of DDR signalling and repair capacity, as we directly demonstrate with the HR efficiency assay.

Nonetheless, we fully agree that the most direct proof of the functional relevance of the physical Giantin–RAD51C interaction would come from separation-of-function experiments, ideally using an interaction-deficient Giantin mutant or an RAD51C variant unable to bind Giantin. We wish to be transparent that both approaches face substantial technical barriers in this system. RAD51C tagging consistently compromised cell viability and protein function, precluding the generation of interaction-deficient variants at physiological expression levels. Engineering an interaction-deficient Giantin mutant presents an independent challenge: Giantin is one of the largest Golgi matrix proteins (~376 kDa), composed almost entirely of extended coiled-coil domains that are resistant to structural prediction, and identifying a discrete RAD51C interaction interface without disrupting broader scaffolding function would require a dedicated structural and biochemical programme. We have framed these explicitly as the most important future priorities in the Discussion (**lines 555-564**), rather than over-interpreting the current data.

5.) The Kaplan-Meier plots in Fig S9 seems to be quite selective in that only breast cancer is shown. Does giantin reduction correlate with poor prognosis in other cancers?
Response: We thank the reviewer for this suggestion. We initially focused on breast cancer because RAD51C is a clinically established hereditary breast and ovarian cancer susceptibility gene (Meindl *et al*, 2010; Ghannoum *et al*, 2023), providing direct clinical context for a study centred on RAD51C dynamics and genome stability. We agree however that restricting the survival analysis to a single cancer type can appear selective.

To address this directly, we expanded the *in-silico* survival analysis of Giantin (GOLGB1) using GEPIA2 (Tang *et al*, 2019) across all available TCGA cohorts (overall survival, median cutoff, FDR correction). In the pooled pan-cancer analysis, higher GOLGB1 expression is significantly associated with improved overall survival (HR(high) = 0.75, p = 6.6×10^{-15}). When stratified by tumour type, the majority of individual associations do not reach statistical significance. The two most robust statistically significant associations are kidney renal clear cell carcinoma (KIRC; HR(high) = 0.57, p = 3.4×10^{-4}), where high GOLGB1 expression is associated with improved survival, and lower-grade glioma (LGG; HR(high) = 1.5, p = 0.036), where the association is in the opposite direction. A significant association is also observed in thymoma (THYM; HR(high) = 7.3, p = 0.031), though this should be interpreted with caution given the small cohort size (n = 59). Notably, the breast cancer association observed in the KM Plotter analysis (HR = 0.71, p = 1.8×10^{-11}; n = 4,929) does not reach significance in the TCGA BRCA cohort (HR = 1.1, p = 0.68; n = 1,070), most likely reflecting the substantially smaller sample size of the TCGA cohort, which is approximately 4.6-fold smaller and therefore underpowered to detect a modest effect. These context-dependent associations are consistent with the tumour-type-specific roles of Golgi scaffolding proteins and are discussed accordingly in the revised manuscript.

In the revised manuscript we have retained the original breast cancer Kaplan-Meier plots and supplemented them with a pan-cancer survival map across all TCGA cohorts (**lines 611-625**; **Figure S9G**) and a summary table (**Supplementary Table 3**) reporting hazard ratios, sample sizes, and p-values for each tumour type, allowing readers to assess the clinical relevance of GOLGB1 expression.

Minor points: There are a few grammatical errors here and there. The figures do not appear in the correct order in the text, which makes the early parts of the paper a bit difficult to follow. Some of the figures don't seem to clearly match the text. For example, it is mentioned that RAD51C labelling was done with 3 different antibodies. I could not find this data.
Response: We thank the reviewer for these helpful observations. In the revised manuscript we have (i) carefully proofread the text and corrected grammatical errors throughout; (ii) revised the Results section to ensure that figures and supplementary figures are cited in sequential order and that each panel is explicitly introduced before being discussed, improving readability in the early sections. and (iii) corrected figure callouts to ensure they match the text. In particular, the statement that RAD51C labeling was performed with three different antibodies has been linked to the corresponding figure panels in the Results section. Antibody identifiers, sources, and dilutions are clearly reported in the Methods and in the table in **Supplementary Table S1**.

Reviewer #1 (Significance (Required)):
This paper is novel and should be of significant interest to the field. It has important implications for how we think about the Golgi apparatus, and for how DNA repair pathways may be controlled. The pattern is clearly complex, with many DNA repair proteins localising to the Golgi, and some showing opposite dynamics. However, by focussing on RAD51C and giantin, the paper nicely demonstrates a novel mechanism for controlling DNA repair by these proteins.

Reviewer #2 (Evidence, reproducibility and clarity (Required)):

Background - *Eukaryotic cells rely on tightly regulated DNA repair pathways to preserve genome stability under the constant threat of both endogenous and exogenous genotoxic stress. While the nucleus, and to a lesser extent the mitochondria, is the primary site where DNA damage is detected and repaired, accumulating evidence indicates that extranuclear organelles, particularly the Golgi apparatus, play a surprisingly important role in modulating stress signaling, proteostasis, and the trafficking/activation of key DNA repair factors.*

Emerging evidence has shown that genotoxic stress can result in a major remodeling of the Golgi apparatus; however, the crosstalk between the Golgi and the nucleus, and its contribution to the DNA damage response, remains poorly defined. The present study offers timely insight by examining the spatiotemporal behavior of DNA repair proteins that shuttle between the Golgi and the nucleus, and how this trafficking contributes to the maintenance of genomic stability.

Main findings - *The authors employed the Human Protein Atlas (HPA) project to shortlist proteins that might link Golgi-nuclear function and validated each candidate using an siRNA-mediated antibody-validation pipeline, thereby identifying 163 proteins that localize to both the Golgi and the nucleus. Bioinformatic analysis of these candidates revealed a significant enrichment for DNA damage response (DDR) regulators, including multiple factors from core DNA repair pathways, suggesting that a portion of the DDR machinery may reside in the Golgi at steady state. Interestingly, the authors observed that dual-localizing DDR proteins undergo lesion-specific redistribution between the Golgi and the nucleus in response to specific types of DNA injuries. For instance, BER and MMEJ proteins shifted from nucleus to Golgi in response to doxorubicin, whereas MMR and HR proteins redistributed from Golgi to nucleus. This trend was reversed with H2O2 or KBrO3 treatments.*

To gain further insight into the link between the DDR and Golgi-nuclear communication, the authors focused on the HR factor RAD51C, which also plays a key role during the replicative stress response. The authors noticed that RAD51 is significantly associated with the Golgi, in addition to its known nuclear pool. Interestingly, they demonstrated that doxorubicin triggers the ATM-dependent release of this Golgi-tethered RAD51C pool and its Importin-β-mediated import into the nucleus, where it forms repair-associated foci. They further identified Giantin as the Golgi scaffold that anchors RAD51C at steady state in this subcellular compartment and showed that its depletion leads to premature nuclear accumulation of RAD51C, formation of aberrant RAD51C foci lacking canonical HR markers, reduced ATM activation, elevated genomic instability, and increased cell proliferation.

Together, this study revealed an underappreciated and functionally meaningful spatiotemporal level of regulation within the DDR, suggesting that the Golgi, rather than functioning solely as a trafficking organelle, acts as a platform that anchors, releases, and temporally controls the availability of key DNA repair factors in response to genotoxic stress. In particular, the authors demonstrated that the timely and regulated release of RAD51C from the Golgi is essential for maintaining genome stability and is dependent on canonical DDR signaling pathways, including ATM activation and Importin-β-mediated nuclear import.

Overall Critique - *This manuscript offers a novel and compelling perspective on the regulation of*

the DDR by positioning the Golgi as an active participant in the spatiotemporal control of DNA repair factors. By integrating multiple experimental layers, including a systematic localization screening, a sub-Golgi mapping, several dynamic redistribution assays, and functional perturbation read-outs, the authors built a strong and coherent case for a biologically meaningful Golgi-nucleus communication axis during the DDR. Therefore, the study is timely and highly relevant for the DNA repair field, with broader implications for our understanding of how subcellular organelles coordinate genome maintenance and cellular homeostasis.

While the manuscript is clearly written and the figures are coherent and supportive of the main findings of the study, several issues should be addressed to ensure full interpretability and reproducibility.

Major Comments

1. Limited use of agents causing genotoxic stress - The authors report intriguing lesion-specific shifts in Golgi-nuclear redistribution, yet much of the mechanistic work relies heavily on doxorubicin, a pleiotropic drug that induces diverse forms of DNA damage beyond DSBs. Expanding the core analysis of the study to include a broader panel of mechanistically defined genotoxins (e.g., etoposide, camptothecin, neocarzinostatin, or ionizing radiation) would substantially strengthen the conclusion that the trafficking patterns reflect damage-type specificity rather than drug-specific off-target effects. Such broader analysis would also clarify whether Golgi-nucleus communication responds differentially to replication-associated breaks, Topo II-dependent lesions, oxidative stress, or crosslinks.

Response: We thank the reviewer for this important point. We would first note that while doxorubicin is indeed pleiotropic, its primary and best-established mechanism of action is the poisoning of Topoisomerase II, leading to DNA double-strand breaks, a mechanism it shares with etoposide (van der Zanden *et al*, 2021; Thorn *et al*, 2011). The additional effects of doxorubicin, including reactive oxygen species generation and chromatin remodelling, are well-documented but secondary to this DSB-inducing activity, as we note in the revised manuscript. Nonetheless the goal of this study was not to comprehensively map lesion-specific trafficking for every DDR protein, but rather to establish the existence of a dynamic Golgi-nucleus redistribution axis and then focus mechanistically on the validated targets, in this case RAD51C. The lesion-dependent redistribution patterns are therefore presented as an initial, hypothesis-generating observation emerging from our screening and characterisation framework. A systematic, lesion-by-lesion dissection of redistribution kinetics across the broader DDR network would represent a substantial additional study and is beyond the scope of the present work.

Importantly, our key mechanistic observations for RAD51C are not restricted to doxorubicin. We tested a panel of genotoxic agents covering mechanistically distinct lesion classes: camptothecin (CPT; Topoisomerase I-associated replication breaks), etoposide (ETO; Topoisomerase II-dependent DSBs), and mitomycin C (MMC; interstrand crosslinks) (**Figures S8A–S8I**). Across all DSB-inducing agents, RAD51C consistently redistributed from the Golgi to the nucleus, demonstrating that this response is not a doxorubicin-specific off-target effect. Notably, RAD51C did not redistribute in response to oxidative lesions induced by hydrogen peroxide or potassium

bromate, consistent with its established role in homologous recombination and DSB repair rather than oxidative damage pathways, as discussed in the manuscript. This lesion-type selectivity provides additional evidence that the Golgi-nuclear redistribution we observe is a biologically specific response rather than a non-selective stress effect.

2. Functional implications of RAD51C redistribution for HR efficiency - Although the study convincingly demonstrates a release of RAD51C from the Golgi and its subsequent nuclear foci formation, it remains unclear how this redistribution influences HR efficiency. Incorporating a functional HR assay (e.g., DR-GFP reporter, RAD51 filament assembly, or fork protection assays) would help determine whether Golgi-anchored RAD51C release is directly required for HR or instead primarily modulates upstream DDR signaling.

Response: We thank the reviewer for this important suggestion. We have performed DR-GFP reporter assays to directly assess HR efficiency following Giantin and RAD51C depletion. Depletion of Giantin reduced HR efficiency to approximately 60% of control levels, and RAD51C depletion to approximately 40%, consistent with the HR reduction previously reported in the genome-wide HR screen (Adamson *et al*, 2012). Co-depletion of Giantin and RAD51C reduced HR to levels comparable to RAD51C depletion alone, suggesting that the effect of Giantin on HR is mediated primarily through RAD51C, consistent with RAD51C being the key effector of the Giantin-dependent spatial regulatory mechanism we describe. These data are included in the revised manuscript (**lines 455-465**; **Figure 5L**).

In addition, the manuscript does not fully reconcile how Golgi-tethering of RAD51C fits with its well-established nuclear roles during replication stress, where timely availability of RAD51C is essential for fork stabilization and restart.

Response: We agree that the nuclear function of RAD51C during replication stress is well established and important to reconcile with our findings. Our imaging data consistently show a detectable nuclear RAD51C population at steady state across all cell lines examined, and we do not propose that RAD51C is exclusively Golgi-localised. We suggest that the two pools serve distinct functional purposes: the constitutive nuclear pool supports ongoing replication fork stabilisation and restart, processes that require RAD51C availability independently of acute DNA damage, while the Golgi-tethered fraction represents a damage-responsive reserve that is released acutely upon DSB induction in an ATM-dependent manner. We wish to be transparent that this two-pool model is speculative at present, formally distinguishing the contributions of each pool would require direct labelling of the Golgi-anchored fraction, which was not technically feasible in this system as discussed above. Nonetheless, this model is consistent with established principles of signal-responsive protein sequestration in cell biology, and is directly supported by our Giantin depletion data: premature release of the Golgi pool leads to aberrant nuclear RAD51C foci lacking canonical HR markers and impaired ATM signalling, demonstrating that unscheduled nuclear accumulation is actively detrimental rather than simply redundant. We have added a paragraph to the revised Discussion explicitly framing the two-pool distinction as a working model and identifying direct pool-identity tracking as an important future direction (**lines 566-587**).

3. Specificity of Giantin-related phenotypes - The phenotypes observed upon Giantin depletion

(e.g., increased micronuclei, comet tail moments, impaired ATM signaling, and elevated proliferation) could partially reflect a global dysfunction of the Golgi rather than RAD51C-specific tethering defects. Although co-depletion of RAD51C provides partial rescue, additional controls examining Golgi integrity, trafficking competence, or rescue with siRNA-resistant Giantin would help confirm specificity and distinguish direct from indirect effects.*

Response: We thank the reviewer for raising this important concern, which was a central consideration throughout our investigation. We address it through three complementary lines of evidence.

First, regarding Golgi structural integrity and trafficking competence: as previously reported, Giantin depletion has not been associated with strong Golgi fragmentation or major morphological alterations (Koreishi *et al*, 2013; Bergen *et al*, 2017; Stevenson *et al*, 2021), and we observed no significant Golgi fragmentation upon Giantin knockdown in our system. Consistent with the literature, Giantin has been implicated in specific cargo trafficking, most notably collagen secretion, rather than general secretory pathway function (Stevenson *et al*, 2021). To directly confirm that general Golgi trafficking competence was preserved in our experimental system, we performed the VSV-G-YFP trafficking assay (Presley *et al*, 1997), a well-established functional readout of general secretory trafficking. Giantin depletion did not result in a significant change in trafficking efficiency compared to control siRNA **(Rebuttal Figure 1)**, consistent with the literature and arguing against a general collapse of Golgi function as the basis for the phenotypes observed.

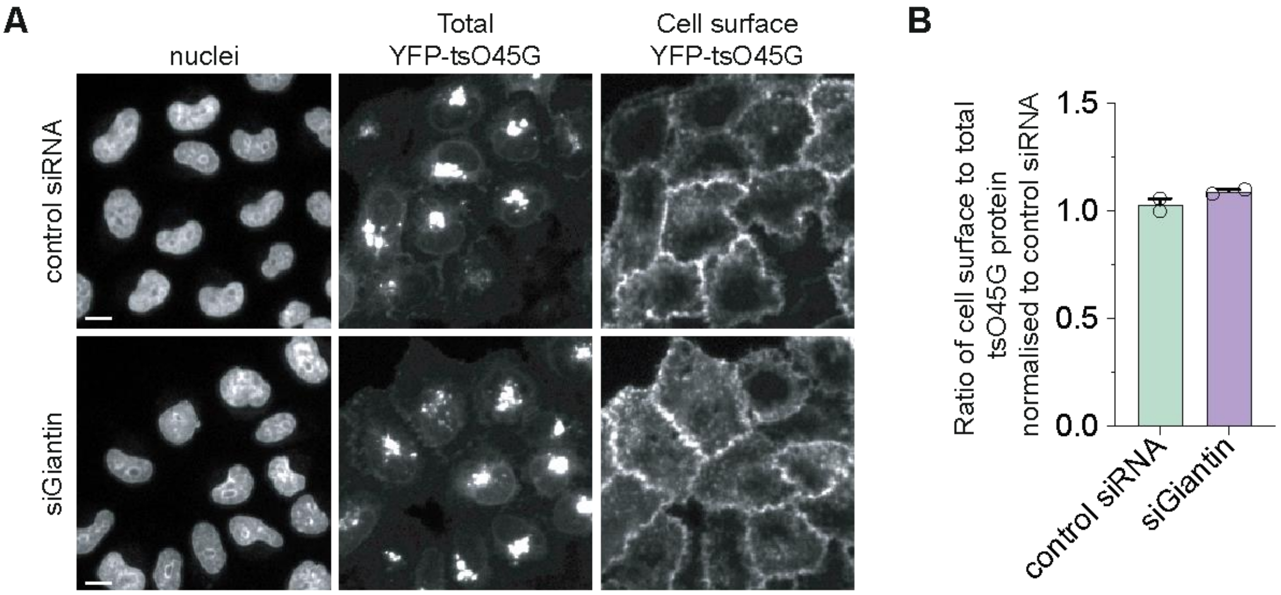

Rebuttal Figure 1. VSV-G-YFP trafficking assay.
(A) *Representative images of cells treated with control siRNA or giantin siRNA. Nuclei are stained with Hoechst. Total VSV-G–YFP (YFP-tsO45G) signal is shown together with antibody staining against VSV-G in non-permeabilized cells to assess cell surface levels. Scale bars, 10 μm.*
(B) *Quantification of VSV-G trafficking from two independent biological replicates.*

Second, the phenotypes are RAD51C-dependent and not a generic Golgi dysfunction: the genomic instability and DDR signalling defects we observe upon Giantin depletion are not phenocopied by GMAP210 depletion, another Golgin family member, indicating that the phenotypes are not a generic consequence of Golgin loss. Critically, we now directly demonstrate using the DR-GFP reporter assay that Giantin depletion reduces HR efficiency to approximately 60% of control, and that co-depletion of RAD51C produces no further reduction beyond RAD51C depletion alone, consistent with RAD51C epistasis over Giantin for HR capacity (**Figure 5L**). This functional epistasis, together with the physical interaction between Giantin and RAD51C by co-immunoprecipitation, their co-localisation within the same Golgi sub-compartment, and the partial rescue of ATM phosphorylation, micronuclei formation and proliferation phenotypes upon RAD51C co-depletion, provides a coherent mechanistic chain linking Giantin specifically to RAD51C-dependent DDR outcomes. While we cannot formally exclude indirect contributions from other Giantin-associated factors, none of our observations are consistent with the phenotype arising from non-specific Golgi perturbation.

Third, Giantin may play a broader role in connecting DDR signalling to cytoplasmic and Golgi-resident processes, beyond RAD51C tethering alone: we consider this a feature of the biology rather than a confound. Golgins are well established as multi-cargo scaffolding platforms, and Giantin in particular occupies a strategic position where several processes converge: the tethering of DDR factors, the regulation of damage-induced signalling cascades, and the directional trafficking of repair factors between compartments. This would explain why Giantin depletion produces a phenotype that extends beyond what RAD51C co-depletion alone can fully rescue, and is consistent with the pathway-level coherence we observe across our screen. Understanding the full complement of Giantin-associated DDR interactions represents one of the most compelling directions emerging from this work.

In response to this comment, we have expanded the Discussion (**lines 545-565**) to explicitly propose that Giantin functions as a broader organisational node coordinating multiple DDR factors, while our data specifically and consistently implicate RAD51C as a primary conduit.

4. Positioning of ATM in the Golgi-nuclear signaling - While ATM inhibition prevents RAD51C release, its spatial and mechanistic basis of this regulation remains obscure. It is not clear whether ATM acts locally at the Golgi, through cytoplasmic pools, or indirectly via nuclear feedback signaling. Clarifying or discussing this point in more depth would improve the mechanistic coherence of the proposed model.

Response: We thank the reviewer for raising this important mechanistic question. The spatial basis of ATM action at the Golgi is indeed an emerging and exciting area of cell biology. A growing body of evidence demonstrates that ATM associates with the Golgi membrane through binding to phosphatidylinositol-4-phosphate (PI4P), and that this Golgi-resident pool modulates the magnitude and kinetics of the nuclear DDR (Ovejero *et al*, 2023). Importantly, the most recent work in this area demonstrates that Golgi-associated ATM is not merely a passive reservoir but is enzymatically active and capable of phosphorylating Golgi-resident substrates (Soulet *et al*,

2026), providing a compelling mechanistic basis for how damage-induced ATM signalling could reach the Golgi to license RAD51C release.

To directly examine whether ATM localises to the Golgi in our system and whether its activation state changes upon DNA damage, we performed a biochemical Golgi enrichment assay using the Minute™ Golgi Apparatus Enrichment Kit (Cat #: GO-037) to examine ATM distribution across *cis*- and *trans*-Golgi fractions. Fraction purity was validated using GM130 (*cis*-Golgi), TGN46 (*trans*-Golgi), and HSP60 (membrane fraction) (**Rebuttal Figure 2A**). This analysis revealed that ATM is detectable in the total membrane fraction and enriched in the *cis*-Golgi fraction under basal conditions (**Rebuttal Figure 2A**). Under normal physiological conditions, activated ATM (pATM) was absent from Golgi-enriched fractions (**Rebuttal Figure 2B**), but was detectable in the *cis*-Golgi fraction following doxorubicin-induced genotoxic stress (**Rebuttal Figure 2C**). While these observations are preliminary and require further validation, they are consistent with the emerging literature and raise the intriguing possibility that ATM is recruited to and activated at the Golgi in a damage-dependent manner, where it could act locally to license RAD51C release.

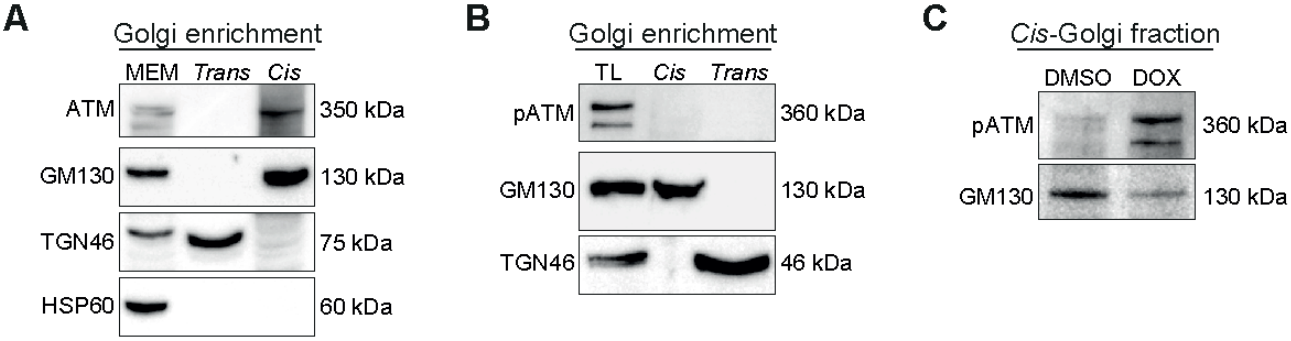

Rebuttal Figure 2. Biochemical Golgi fractionation confirms ATM enrichment in cis-Golgi compartments.
Western blot of HeLa-K fractions enriched for cis- and trans-Golgi membranes, probing for (A) ATM under basal conditions, and (B and C) pATM under basal conditions and (B) pATM (C) after treatment with DOX (40 µM) (markers: GM130 for cis-Golgi, TGN46 for trans-Golgi, HSP60 for membrane fraction (MEM).

We consider the precise spatial and mechanistic dissection of ATM signalling at the Golgi and its relationship to nuclear feedback, one of the most exciting directions to emerge from this work, and one that we hope our study has helped to open. We have expanded the Discussion (**lines 525-543**) accordingly to place our findings in the context of the emerging Golgi-ATM literature and to frame this as an important unresolved question for future investigation.

5. RAD51C is examined in silo, without consideration for the BCDX2 complex - RAD51C is exclusively analyzed in isolation, despite its well-established function as part of the BCDX2 paralog complex (RAD51B-RAD51C-RAD51D-XRCC2). Because RAD51C does not normally operate as a standalone factor, it is unclear why only RAD51C, among all paralogs, would be

subjected to Golgi tethering, ATM-dependent release, and Importin-β-driven nuclear import. This raises important mechanistic questions: Are other BCDX2 members also Golgi-associated? Do they undergo similar trafficking dynamics? Does Golgi tethering selectively regulate RAD51C, or does the complex translocate together? Addressing these points would greatly strengthen the biological plausibility and mechanistic coherence of the proposed model.

Response: We thank the reviewer for raising this important point. We fully agree that RAD51C functions as a core component of the BCDX2 (RAD51B–RAD51C–RAD51D–XRCC2) and CX3 (RAD51C–XRCC3) paralog complexes, and that its canonical roles in HR and replication fork protection occur within these assemblies. Our decision to focus on RAD51C was driven by the screening data: of the DDR proteins identified, RAD51C displayed the most robust Golgi-associated pool, the clearest damage-induced redistribution dynamics, and a tractable anchoring interaction with Giantin that could be interrogated biochemically.

We would also note that extending this analysis to other RAD51 paralogs is not straightforward with current tools. The available commercial antibodies against RAD51B, RAD51D and XRCC2 perform poorly in immunofluorescence applications, and most localisation studies for these proteins have relied on overexpression of tagged constructs, a strategy that, as discussed above, risks perturbing both localisation and complex assembly. The lack of reliable antibodies for endogenous paralog detection at the resolution required for Golgi localisation analysis represents a genuine technical barrier that we encountered directly during this study.

Whether Golgi association and ATM-dependent release involve RAD51C alone or extend to other BCDX2 or CX3 members is therefore a genuinely open and important question. We note that our co-immunoprecipitation data were performed on total cell lysate and cannot distinguish whether the Golgi-associated RAD51C is complexed with other paralogs or represents a monomeric subpopulation. Golgins are well established as multi-cargo scaffolding platforms, and it is entirely plausible that Giantin organises a broader paralog module rather than tethering RAD51C as an isolated subunit. A systematic analysis of RAD51 paralogs for Golgi localisation and lesion-dependent trafficking enabled by improved reagents such as proximity labelling or endogenous tagging approaches compatible with essential proteins would determine whether the BCDX2 complex translocates as a unit or whether individual subunits are differentially regulated, with potentially distinct consequences for HR fidelity. We have revised the manuscript accordingly and identify this as an explicit priority for future work in the revised Discussion (**lines 583-602**).

Minor Comments

1. Pathway-specific sub-Golgi localization patterns - The finding that DDR proteins map to distinct cis/trans Golgi subdomains is an interesting and potentially important observation. However, the dataset is limited to 15 proteins, making the proposed pathway-level trends (e.g., HR factors enriched in cis-Golgi; BER/MMEJ factors enriched in trans-Golgi) preliminary. Strengthening this conclusion by increasing the number of DDR proteins analyzed would help determine whether sub-Golgi compartmentalization contributes meaningfully to DNA repair pathway regulation.

Response: We thank the reviewer for this constructive suggestion. We agree that extending sub-Golgi mapping to a larger number of DDR proteins would be valuable, and we present the current dataset explicitly as a first, hypothesis-generating map rather than a definitive pathway atlas.

We would like to highlight, however, that the value of this observation lies not simply in the number of proteins mapped, but in the biological coherence of the patterns that emerge. The finding that proteins from the same repair pathway tend to occupy the same Golgi sub-compartment: BER and MMEJ factors enriching in the *trans*-Golgi, HR factors in the *medial/cis*-Golgi, and that this sub-compartmental positioning correlates with the direction of their redistribution upon genotoxic stress, is a pattern that would be unlikely to arise by chance across 15 independently validated proteins. This internal consistency argues that the sub-Golgi organisation reflects genuine pathway-level biology rather than noise, even if the dataset is not yet exhaustive. Together with the bioinformatic network analysis, which independently supports pathway-level clustering across the broader validated hit list, these observations reinforce each other as complementary layers of evidence.

2. Is the Golgi-released RAD51C indeed the pool that enters the nucleus? The major assumption of the study is that the RAD51C population released from the Golgi upon DNA damage is the same pool that subsequently accumulates in the nucleus to form repair foci. While the imaging and fractionation data are consistent with this model, the study does not directly track or distinguish Golgi-derived RAD51C from cytoplasmic or pre-existing nuclear pools. Without a method to specifically label, pulse-chase, or track the Golgi-anchored fraction, it remains formally possible that nuclear RAD51C originates from other subcellular reservoirs.
Response: We thank the reviewer for highlighting this important mechanistic point, which we agree cannot be fully resolved with the current dataset. Several independent lines of evidence are nonetheless consistent with a model in which the Golgi-associated pool contributes directly to damage-induced nuclear accumulation.

- Our time-resolved imaging demonstrates a reciprocal decrease at the Golgi and a concurrent increase in the nucleus following genotoxic stress, consistent with redistribution rather than independent compartment-specific changes (**Figures 3E–3I**).
- Biochemical fractionation provides an orthogonal readout of the same reciprocal shift under identical conditions (**Figures 3J and S6D**).
- ATM inhibition simultaneously prevents Golgi loss and blunts nuclear accumulation, while Importin-β perturbation blocks nuclear entry, together supporting an active and regulated translocation route (**Figures 3K–3P**).
- Giantin depletion, which releases the Golgi-tethered RAD51C pool prematurely, leads to aberrant nuclear RAD51C foci lacking canonical HR markers and impaired ATM signalling, strongly supporting that the Golgi-tethered fraction has functional consequences in the nucleus consistent with it being the relevant pool (**Figures 4B–4E and 4J–4M**).
- In the revised manuscript we have included cytoplasmic RAD51C signal quantification across the doxorubicin time course (**Figure 3H**). The cytoplasmic signal shows only a moderate and gradual reduction that is kinetically distinct from the sharp Golgi decrease and does not precede the nuclear increase. This pattern is inconsistent with a large pre-

existing cytoplasmic reservoir driving the nuclear accumulation; if the cytoplasmic pool were the primary source, one would expect a rapid and prominent cytoplasmic decrease coinciding with or preceding nuclear accumulation, which we do not observe. Instead, the data are more consistent with rapid transit of Golgi-released RAD51C through the cytoplasm rather than stable cytoplasmic accumulation prior to nuclear entry.

We acknowledge that definitive pool-identity tracking would require spatially restricted labelling approaches such as Giantin-proximal TurboID or photoactivatable tagging strategies, which are precluded by the technical constraints on RAD51C tagging described above. We have revised the manuscript to avoid overstatement on this point and identify these approaches as important future directions (**lines 297-305 & lines 715-719**).

Reviewer #2 (Significance (Required)):

General assessment - This study presents a novel and conceptually compelling view of the DNA damage response (DDR) by positioning the Golgi apparatus as an active regulator of the spatiotemporal availability of DNA repair factors. The strongest aspects of the work include its integration of a systematic immune-localization screening, a sub-Golgi compartment mapping, dynamic redistribution assays, and functional perturbations to build a coherent model of Golgi-nucleus communication during genotoxic stress. The mechanistic focus on RAD51C provides a clear case study linking organelle-level regulation to genome stability.

Advance - To my knowledge, this is the first comprehensive demonstration that the Golgi can serve as a spatiotemporal coordination node for DDR proteins, including those involved in HR. The identification of a substantial pool of RAD51C, and reportedly other DDR factors, anchored within specific Golgi subdomains represents a significant conceptual advance. The demonstration that Golgi-tethered RAD51C is released in an ATM-dependent manner and subsequently participates in nuclear foci formation suggests a previously unrecognized organelle-level regulatory checkpoint in genome maintenance. This work therefore extends current models of the DDR by revealing a layer of intracellular coordination that bridges classical nuclear pathways with cytoplasmic organelle function.

Audience - This study will be of strong interest to a specialized audience in the fields of DNA repair, genome stability, and cell biology, particularly those studying the spatial organization of repair pathways and intracellular stress signaling. It will also appeal to researchers investigating organelle biology, intracellular trafficking, and the broader coordination of cytoplasmic and nuclear responses to stress. Beyond these communities, the work may be relevant to cancer, as it suggests new mechanisms by which organelle perturbations or Golgi-associated scaffolding proteins could influence therapeutic responses or genomic instability.

Reviewer expertise - Field of expertise: DNA repair, genome stability, organelle biology, cancer cell biology.

Reviewer #3 (Evidence, reproducibility and clarity (Required)):

This study investigates the communication between the Golgi complex and the nucleus of the cell, which remains a largely unexplored field. The authors used publicly available siRNA and antibody data from the Human Protein Atlas as a basis for finding overlap between the proteomes of the two cellular compartments. In validating the data from the HPA, the study finds a novel cluster of DNA repair proteins present in the Golgi, which they validate and resolve to sub-compartmental localization. To do so they use immunofluorescence (IF) localization on ¬cis- and trans-Golgi cisternae marked by GM130 and TGN46, respectively. The authors find that many of the fully validated proteins present in both the nucleus and Golgi redistribute between the Golgi and the nucleus dependent on the protein and the type of DNA lesion. They focused on RAD51C, a recombination factor. They show that RAD51C resides in both the ¬cis- and trans- subsections prior to damage and responds to DNA damage in an ATM-dependent manner via release of a Golgi-based pool bound to Giantin, which is then imported into the nucleus via Importin-β. Knockdown experiments showed that Giantin regulates RAD51C spatially and temporally. The work reveals a dynamic interchange of proteins between the Golgi and nucleus that controls cell functions beyond the classic secretory, membrane trafficking, and PTM roles of the Golgi. The authors build on prior work on Golgi impacts on DDR, offering an alternative cellular compartment for storage of DDR factors prior to damage. Overall, the data is timely and relevant, as it finds new roles for the Golgi in DNA damage response (DDR) regulation. The data is largely convincing and well controlled. The IF data is presented in black and white single channels and merged in color, which allows good comparison of the different protein stains. The scope of the initial screen of HPA antibodies and Golgi/Nuclear dual proteomes is impressive, and the overlap of DDR proteins is characterized for fifteen different proteins at a sub-compartmental level. The focus on RAD51C as a member of the HR pathway was a strong choice, and the study presents interesting information on its regulation by Golgi complex members, as well as a feedback look with pATM. The possibility of the Golgi storing specific DDR factors in specific compartments is well-supported and intriguing. There are a few major and minor points that should strengthen the paper and improve clarity prior to publication.

Major Comments:

1. Much of the strength of the IF data is lost in the choice of scale for presentation of the data. In almost all cases, enlarged sections should be shown of the areas currently indicated by arrow, in all channels. This is done well in Figure 3A, where an area of the Golgi is enlarged and the overlap of RAD51C in the GM130-marked Golgi is clearly visible in the merged channel, even when printed out. I would highly recommend including the white box and enlarged in all images and channels, while keeping the representative fields as is (e.g. if the image is 40mm, draw a 7mm box around representative cells/Golgi, and enlarge to 15mm in the bottom left). This change should be made to F1E, F2F, F3E, F3J, and F3M, as well as having enlarged figures in the corners in all supplementary data IF figures. Where possible, a fully enlarged image of the bounding box could also be included. Some of the IF data would be strengthened by using the nuclei stain to draw a masking outline to include in the black and white channels, to clearly delaminate what is Golgi-localized and what is nuclear.

Response: We thank the reviewer for this helpful suggestion and fully agree that enlarged insets substantially improve the visibility of Golgi-localised signal, particularly when figures are printed. We share the reviewer's view that alternative display formats with larger insets would be preferable, and we have implemented enlarged boxed regions wherever space constraints permitted.

Specifically, we have added boxed regions with enlarged insets to Figure 1E, all panels of Figure 3. For Figure 2, the number of conditions and proteins displayed simultaneously within the constraints of standard journal figure dimensions made it impractical to include enlarged insets for all panels without reducing the overall field size to the point of losing contextual information. We have nonetheless improved the visibility of the Golgi signal in Figure 2 as much as possible within these constraints, and note that the final figure layout will be further optimised in line with the journal's specific formatting guidelines. In addition, all figures have been provided as high-resolution image files to allow electronic magnification, enabling readers to inspect the Golgi-localised signal in detail beyond what is visible in the printed version.

Regarding the use of nuclear outline masks in single-channel images, we tested this approach but found that given the number of structures present within each field, including Golgi stacks, nuclear foci, and cytoplasmic signal, overlaying nuclear outlines on individual channels added visual complexity that made the images harder rather than easier to interpret. As an alternative, we have included a full-colour merged panel, when possible, which we consider a cleaner way to delineate nuclear versus Golgi-localised signal and allows the reader to directly compare compartment-specific distributions across channels.

2. There is a lack of consistency in the representative images shown by IF. For example, Figure 1 gives the impression of very little RAD51C in the nucleus but this is rightly shown to not be the case in Supp. Fig 2A. The same is true of the various images of LIG1. The authors should use representative data that better reflects the distribution of the proteins being studied and maintain consistency across images. If there is a lot of variation in staining patterns, the authors should show images and percentages corresponding to the variations especially for the key gene studied, RAD51C.

Response: We agree and have replaced the representative IF panels for RAD51C and LIG1 with images that better reflect the quantified distributions across biological replicates. The revised panels were selected to match the quantified compartment intensities shown in the accompanying graphs rather than representing outlier cells. We would also note that the apparent discrepancy between **Figure 1E** and **Supplementary Figure S2A** partly reflects a difference in imaging conditions: **Supplementary Figure S2A** and **Figure 2F** were acquired directly from the high-content screening pipeline under uniform, non-optimised antibody and fixation conditions at widefield resolution, whereas **Figure 1E** shows representative single optical section confocal images acquired after candidate identification with antibody conditions optimised for each individual protein. The improved signal-to-noise in the optimised confocal images more faithfully captures the dual Golgi and nuclear localisation of RAD51C, and the apparent difference between the two image sets is therefore expected rather than inconsistent. We have updated the figure

legends to clarify the imaging modality and conditions for each panel. Furthermore, the quantified distribution of RAD51C across Golgi, nuclear and cytoplasmic compartments across multiple cell lines is shown in **Figure 3B** and **3D**, providing a population-level representation of the dual localisation that complements the representative images shown in **Figure 1E**.

3. The initial screening by siRNA-mediated knockdown pipeline that validated and confirmed dual Golgi and nuclear localization of 163 of the 329 dual-localization HPA proteins does not have any data included. This seems like a very large amount of data to gloss over and not include even as supplementary data. This should be included as source data, and discussion of the in-text information should be strengthened. The data included with the networking of these validated proteins is strong, but the process of elimination and validation has not been shown. In addition, the antibody information included in the supplementary data does not include dilution factors or blocking factors is not included, which would be beneficial to future studies to include.

Response: We agree and have addressed this in full. We note that the HPA antibody validation data, including immunofluorescence images and siRNA knockdown results, are publicly available for inspection on the Human Protein Atlas website (www.proteinatlas.org) for the majority of candidates, providing an independent layer of verification. In the revised submission, we additionally provide the complete siRNA-mediated validation dataset generated in our laboratory as source data (**Table S1; lines 1025-1041**), including for each candidate the HPA antibody identifier, gene symbol, Ensembl ID, antibody staining pattern, siRNA identifier, cell number per replicate, and normalised Golgi and nuclear signal ratios for both experimental replicates. This allows readers to inspect the validation metrics directly and apply alternative thresholds if desired. We have also expanded the antibody information to include diluent conditions (4% FBS in 0.1% Triton-X100 for all HPA antibodies used at 2 μg/ml in the screening pipeline), enabling reproducibility and reuse of the dataset by the community.

4. The authors should expand upon the paragraph lines 155-162 to include more discussion on Figure S2A and S2B. The expanse of this data is some of the strongest in the paper, and it should be further discussed in-text. Also, the rationale behind the choice in the specific proteins that are included in these analysis / figures is not always clear in -text, and more attention should be spent on the narrowing down of the analysis to the final proteins. This is also especially important as many of the DDR proteins chosen are not the most common DDR proteins. Also note in text that the Golgi marker GM130 (presumably) was used for the screening, which means that some proteins which are only localizing to the TGN46 trans Golgi might have been lost in the validation step (or, explain why this is not the case).

Response: We expanded the Results text (**lines 141-163**) to discuss Figures S2A and S2B in more depth and clarified the rationale for selecting the final set of DDR proteins taken forward, including considerations of pathway representation, bioinformatic annotations, literature-described roles in DNA repair. We would also note that the identity of the DDR proteins identified in this screen was determined by the HPA dataset and the unbiased validation pipeline rather than by prior assumptions about which repair factors would be present at the Golgi. The presence of less commonly studied DDR factors is therefore a direct reflection of the screen output, and we consider this one of the strengths of the approach.

We would also like to address the reviewer's concern about potential GM130-based bias directly: at the widefield or confocal resolution used in the high-content screening pipeline, the Golgi apparatus appears as a single perinuclear structure and cis- and trans-Golgi subdomains cannot be resolved. GM130 was therefore used purely as a segmentation marker to define the Golgi compartment as a whole rather than to selectively label the cis-Golgi cisternae. The resulting Golgi mask captures signals from the entire Golgi ribbon, including trans-Golgi regions, meaning that proteins with exclusively trans-Golgi localisation would not have been systematically excluded at the screening stage. Sub-compartmental resolution of cis versus trans localisation was only possible in subsequent analyses using nocodazole-dispersed mini-stacks imaged by confocal microscopy with co-staining for both GM130 and TGN46.

5. The relationship between Giantin loss, increased cell proliferation, and elevated endogenous DNA damage as it relates to RAD51C remains insufficiently resolved and requires further clarification. Several of the proliferation assays used are not optimal for addressing changes in cell growth. For example, Figure 5O appears to quantify cell numbers by counting fields from IF images, which is an unconventional approach. This should be done by growth curves, luminescent viability or colony formation assays. In addition, this point will be greatly strengthened by performing rescue experiments for Giantin directly (instead of co-depletion as a means of rescue) and/or using a mutant of RAD51C that does not bind to Giantin. If these additional experiments are beyond the current scope, the conclusions should be softened in the discussion.
Response: We thank the reviewer for raising these important points, which we address in turn:

Giantin–RAD51C relationship and mechanistic interpretation. We acknowledge that establishing the full causal chain between Giantin loss, RAD51C mislocalisation, elevated endogenous DNA damage and increased cell proliferation is challenging within the scope of a single study, and we discuss this openly in the Discussion (**lines 555-564**). Our evidence collectively includes: physical interaction between endogenous Giantin and RAD51C by co-immunoprecipitation (**Figures 4H and 4I**), premature nuclear accumulation of RAD51C upon Giantin depletion (**Figures 4B–4E and 4J–4M**), new additional experiment showing direct reduction of HR efficiency in the DR-GFP assay (**Figure 5L**), impaired ATM signalling (**Figures 5J and 5M**), elevated genomic instability (**Figures 5A–5E**), and epistatic rescue by RAD51C co-depletion (**Figures 5M–5P**). These observations are further contextualised by the established literature on RAD51C function: RAD51C is known to regulate CHK2 phosphorylation and cell cycle checkpoint signalling (Badie *et al*, 2009), stabilise replication forks (Somyajit *et al*, 2015), and promote RAD51 filament formation required for DSB repair (Prakash *et al*, 2015). Dysregulation of these functions through Giantin-dependent mislocalisation provides a mechanistically coherent explanation for the elevated genomic instability and altered proliferation we observe, and is entirely consistent with our model. Together, the experimental evidence and the published biology of RAD51C support a model in which Giantin spatially regulates RAD51C to maintain proper DDR signalling and HR capacity.
We agree that separation-of-function tools would further strengthen this model and identify these as important future priorities. We wish to note however that both approaches face substantial

technical barriers in this system. As described in our response to Reviewer 1 Major Comment 1, RAD51C tagging, whether by CRISPR-mediated endogenous editing or ectopic expression, consistently compromised cell viability and protein function, precluding the generation of interaction-deficient variants at physiological expression levels. Engineering an interaction-deficient Giantin mutant presents an independent and considerable challenge: Giantin is one of the largest Golgi matrix proteins (~376 kDa), composed almost entirely of extended coiled-coil domains that are intrinsically difficult to model structurally, and identifying a discrete interaction interface with RAD51C without disrupting the broader scaffolding function of the protein would require a dedicated structural and biochemical programme. We therefore consider these important but substantial future directions rather than straightforward experimental additions to the current study.

Proliferation assays. Colony formation assays provide a rigorous readout of long-term proliferative capacity, and these data are presented for single knockdown conditions in **Figures 5F–5I**. The cell number quantification in **Figure 5P** was specifically included to assess the double knockdown of Giantin and RAD51C simultaneously, a condition not covered by the colony formation assay. We respectfully note that automated fluorescence microscopy-based nuclear counting is a well-established approach for measuring cell proliferation in siRNA screening contexts. Nuclear counting from high-content imaging has been used as a direct readout of cell growth and proliferation in RNAi screens (Boutros *et al*, 2004; Martin *et al*, 2014; Garvey *et al*, 2016; Mikheeva *et al*, 2024), and has been shown to produce results comparable to or superior to conventional viability assays including MTT and flow cytometry-based methods (Mikheeva *et al*, 2024). We have nonetheless clarified in the revised figure legend that Figure 5P reports relative cell number quantified by automated nuclear counting from high-content imaging fields as a secondary concordant measure alongside the colony formation data, rather than a standalone proliferation assay.

6. It is unclear from the discussion and from presented data whether proteins are directly transported between the Golgi and the nucleus, or whether they go into the cytoplasm for a transient period, presumably when they could interact with Importin β. There is also some data where cytoplasm signal could be quantified to address this (Figure 3E-I).

Response: We thank the reviewer for this mechanistic point. In the revised manuscript we have included cytoplasmic RAD51C signal quantification alongside Golgi and nuclear measurements for the doxorubicin time course (**lines 297-305**; **Figure 3H**). The cytoplasmic signal shows a moderate and gradual reduction distinct in both magnitude and kinetics from the sharp Golgi decrease, consistent with a transient cytoplasmic intermediate rather than a stable pool. Regarding the identity of the translocating pool, two observations directly support a Golgi origin. First, Importazole treatment prevents RAD51C release from the Golgi following genotoxic stress and simultaneously reduces nuclear RAD51C foci formation, demonstrating that Importin-β-mediated import is required both for Golgi clearance and for productive nuclear accumulation. Second, Giantin depletion which prematurely releases the Golgi-tethered pool, leads to aberrant nuclear RAD51C foci, directly linking the Golgi-anchored fraction to nuclear accumulation. Together these data support a model in which Golgi-resident RAD51C transits through the

cytoplasm for Importin-β-mediated nuclear import. We acknowledge that without direct labelling of the Golgi-anchored fraction, the precise contribution of each subcellular pool to the nuclear accumulation cannot be fully resolved with the current dataset. We discuss the development of appropriate tagging strategies as an important future direction to dissect the dynamics of this process in further detail.

7. Statistical analysis on experiments with more than two samples need to be performed with ANOVA and a follow up post-hoc test, not with two-tailed unpaired Student's t-test, which only compares the control and each individual sample. This type of analysis inflates the Type 1 error rates (false positives) in your datasets. For example, the two-tailed unpaired Student's t-test is appropriate in Figure 2F-H, but not in Figure 3 when the samples are timepoints. In this case, a One-way ANOVA with Tukey's post-hoc test (if you want to show all coparisons), or Bonferroni/Sidak if you only need to compare several samples).

Response: We agree with the reviewer and thank them for highlighting this important statistical issue. We have revised the statistical analysis for all experiments involving more than two groups to avoid inflation of Type I error rates caused by multiple pairwise Student's t tests. Specifically, for Figures 3F–I, 4C–E, and Figure 5, the data were reanalysed using one way ANOVA followed by the appropriate multiple comparisons post hoc test. The Methods section and corresponding figure legends have been updated to clearly state the statistical tests used for each dataset.

Minor Comments:

General

1. Throughout the text, the reference to many figures and supplementary figures in the same sentence, with little discussion of the data therein makes it hard to follow. In-text referencing is particularly confusing in the section "Dual-localising DDR proteins dynamically redistribute between the Golgi and nucleus in response to specific types of DNA injuries," where the reader is switching between multiple figures and supplementary figures.

Response: We thank the reviewer for this helpful comment. In the revised manuscript, we have improved the readability of the text and revised the figure references to make them clearer. We hope these revisions make the manuscript easier to follow and allow readers to better inspect the figures.

2. In figures that display technical replicates as individual data points, consider distinguishing each replicate by using different marker shapes (e.g., repeat 1 = upright triangle; repeat 2 = inverted triangle; repeat 3 = diamond). This would provide additional clarity regarding the consistency and repeatability of each technical repeat.

Response: We thank the reviewer for this suggestion. We have updated the data presentation to distinguish biological replicates using different marker shapes in datasets where replicate tracking is of particular relevance to the interpretation. For datasets where individual replicate values are already clearly separable, we have maintained the existing presentation to avoid unnecessary visual complexity.

8. Make sure all western blot data includes the marker size (F3C and F5L has none, F4H/I have size of proteins not size of markers).
Response: We added missing marker sizes to our western blot data in the revised manuscript.

9. Be consistent with use of capitalization in figure legends and graph/figure labels.
Response: We made sure that the capitalisation is consistent in figure legends, graph and figure legends in the revised manuscript.

Figure 2
1. In Figure 2A, please include in the figure itself that GM130 is the cis Golgi, and TGN46 is the trans Golgi (Figures should not be dependent on the text for full understanding).
Response: We revised Figure 2A and 2C to label GM130 as *cis*-Golgi and TGN46 as *trans*-Golgi within the figure, making it self-explanatory.

2. Why are LRIG2 and LRRIQ3 not included in the 2E cis vs trans Golgi data, when all other proteins from F1D are included? Include, or comment on in-text.
Response: Both LRIG2 and LRRIQ3 are included in 2E in both the original and revised manuscript.
3. Be sure to include scale bar data in each figure legend (F2A-E is currently missing it), and include updated scales included in the enlarged data.
Response: Scale bar data is now included in each figure legend in the revised manuscript.

4. In Figure 2F, make sure that the merged green channel is presented at the same intensity as it is in the single black and white channel, as the green looks very overexposed in several of the merged (CCAR1 DMSO merged is the most noticeable).
Response: We agree and thank you for pointing this out. We have now revised the images and corrected the issue by updating all image panels in the figure.

5. In Figure 2G, include the grey label in the figure legend.
Response: We thank the reviewer for this comment. The grey label has now been included in the figure legend in the revised manuscript.

6. In Figure 2G-H, the method of data presentation in the graphs coupled with the statistical analysis is confusing and should be expanded upon in the legend.
Response: We agree that the amount of data presented may appear overwhelming. In the revised figure, we have adjusted the placement of the statistical annotations to improve clarity. Also, we improved the figure legend, to make the figure easier to read and interpret.

Figure 3
1. Figure E/F/G: Is there cytoplasmic quantification as well? Your rationale is that the Golgi RAD51C goes into the nucleus, but via the cytoplasm (due to Importin β import); do you see the cytoplasmic levels increase? Or is it too dilute to notice a difference? At least, this omission needs to be mentioned in-text.

2. Figure H/I also include the quantification of the cytoplasmic fraction. It is mentioned in-text on line 272, but not quantified. This comes up as a big question: Do the proteins go directly between the Golgi and nucleus, or do they go through the cytoplasm?
Response: We thank the reviewer for both of these related points. As described in our response to Major Comment 6 above, we have added cytoplasmic RAD51C signal quantification to the doxorubicin time course in the revised manuscript (Figure 3H) and discuss the implications for the proposed translocation route.

3. Figure 3A, 3E, and if the data is present for 3J and 3M, could all benefit from using the nuclei staining as a mask to draw an outline around the nucleus in the other channels, and then show a merge in full color instead of a nuclei-only channel. Also note from the major comments, that this data especially is so small to see without enlarged images.
Response: We thank the reviewer for this suggestion. Regarding nuclear outline masks, we tested this approach but found that the number of structures present in each field, including Golgi stacks, nuclear foci and cytoplasmic signal, made overlaid outlines visually confusing rather than clarifying. We have instead included a full-colour merged panel in Figure 3E, which we consider a cleaner way to distinguish nuclear from Golgi-localised signal while preserving the spatial context of the data.

Regarding image size, we have added enlarged insets to Figures 3E, 3J and 3M in the revised manuscript. We have chosen to display multiple cells per panel rather than a single enlarged cell in order to capture the heterogeneity of the cell population, which we consider important for an accurate representation of the data. All figures have been provided as high-resolution image files to allow electronic magnification, enabling detailed inspection of the signal beyond what is visible in the printed version. We acknowledge that the constraints of standard journal figure dimensions limit how large individual panels can be, and the final layout will be optimised in line with the journal's formatting guidelines.

4. In-text discussion of the results from Figure 3 has an in-depth discussion of the NLS and NES in RAD51C, but this is not followed up on with site-directed mutagenesis or any data; perhaps move this to the discussion instead of results section.
Response: We have removed the discussion of the NLS and NES from the Results section.

Figure 4
1. Comments from earlier figures hold, with size of enlarged events and using the nuclei as an outline in the single channels. E.g. Figure 4F arrows appear to point to nothing at the chosen scale. The zoom in 4G is insufficient, as the chosen feature is so small it is not even visible in full fields.
Response: We thank the reviewer for this comment. The arrows in Figure 4F indicate individual nocodazole-dispersed Golgi mini-stacks, which are displayed at higher magnification in Figure 4G. The full field in Figure 4F is intentionally shown to illustrate the degree of Golgi dispersion achieved by nocodazole treatment, a context that may be unfamiliar to readers outside the Golgi

field, before zooming into a single representative mini-stack in Figure 4G for the cisternal localisation analysis.

2. Figure 4H and 4I need to show the size of the markers
Response: The size of the markers are now included in the revised manuscript.

3. The representative image in 4L for siGiantin pATM has no pATM foci, while the quantification in 4M has a reduction from ~50% to ~25%, so this image is not representative of this data, or the data quantification is not as strong as the actual data.
Response: We thank the reviewer for this observation. We wish to clarify that the quantification in Figure 4M reports the mean percentage of RAD51C foci co-localising with pATM across the entire cell population from three independent biological replicates. A reduction from ~50% to ~25% therefore reflects a population-level shift in co-localisation frequency, not that every individual cell shows exactly 25% co-localisation. Given the inherent cell-to-cell variability in foci number and co-localisation, individual cells will span a range of values around this mean, and the representative image shown in Figure 4L reflects one such cell.

Figure 5
1. Figure 5A has overexposure of the nuclei stain in order to visualize micronuclei. Readjust the levels, and enlarge the images for better visualization. (is this DAPI-stained? Please label).
Response: The display levels of the nuclear stain in Figure 5A are intentionally set to allow visualisation of micronuclei, which are significantly dimmer than the main nucleus and would not be detectable at display settings optimised for the primary nuclear signal. This is standard practice in micronuclei quantification studies and is necessary to accurately identify and score these structures. The nuclear stain is Hoechst 33342, and this has been explicitly labelled in the revised figure legend.

2. Figure 5A-C: Figure 5A does not show siRAD51, but it is included in the DMSO only graph. Please either show RAD51 data in 5A and 5C, or do not include in 5B. If the DMSO and ETO experiments were performed separately and that accounts for this discrepancy, then show separately.
Response: We thank the reviewer for this observation. The siRAD51C condition is included in Figure 5B as an internal positive control, consistent with its well-established role in genome stability. RAD51C depletion combined with etoposide treatment resulted in severe cellular toxicity and insufficient cell numbers for reliable quantification, and this condition was therefore excluded from Figure 5C. This has been clarified in the revised figure legend.

3. Figure 5M the white label is difficult to see in the green box.
Response: We have updated the label colour in Figure 5M to improve visibility against the green background in the revised manuscript.

Supplementary Figures
1. Consider reordering/ subdividing supplementary figures for ease of reference during reading.

Response: We thank the reviewer for this suggestion. The current supplementary figure structure was intentionally designed to minimise the total number of supplementary figures and maintain a logical correspondence with the main figures, avoiding a situation where readers need to navigate an extensive supplementary section, a concern the reviewer raised regarding figure presentation. We believe the current organisation achieves a reasonable balance between completeness and accessibility.

2. SF1 and SF2A: Include enlarged boxes or full images so that data is visible.
Response: As described in our response to Major Comment 1, all figures have been provided as high-resolution image files to allow electronic magnification. Space constraints within standard journal figure dimensions preclude the addition of enlarged insets to all supplementary panels without substantially reducing the contextual field of view.

3. SF3A, SF4A, and SF5A: Include enlarged images, include nuclei marker if possible (otherwise, the nuclear intensity is not proven nuclear).
Response: We appreciate the suggestion, but adding enlarged insets and nuclei markers to all panels in Figures S3A, S4A and S5A would disproportionately increase the length and complexity of the supplementary section, making it harder rather than easier to navigate. The nuclear intensity measurements are derived from automated segmentation of the Hoechst channel using CellProfiler, which reliably defines nuclear boundaries independently of the antibody channel, and are therefore not dependent on visual confirmation of nuclear localisation in each representative image.

4. SF3B-C, SF4B-C, and SF5 B-D: Change the data presentation in the same method as changed for F2G-H.
Response: We have updated the figure legends for Figures S3B–C, S4B–C and S5B–D to improve readability.

5. SF3D: List proteins in the same order as in B and C.
Response: The proteins in Figure S3D are listed in the same order as in Figures S3B and S3C

6. SF6D: Label M N and C more clearly. Include size labels.
Response: We have added clearer labels for the membrane (M), nuclear (N) and cytoplasmic (C) fractions and included molecular weight size markers in the revised Figure S6D.

7. SF7A-B: Include enlarged.
Response: We respectfully note that the purpose of Figures S7A–B is to display the overall cellular response to inhibitor treatments across the cell population, rather than to highlight specific subcellular structures. Enlarged insets would reduce the number of cells visible per panel and would not add scientific value in this context. The Golgi and nuclear signals are clearly visible at the chosen magnification.

8. SF8: Include arrows as in previous experiments, include enlarge.

Response: Arrows have been added to Figure S8 to indicate Golgi and nuclear RAD51C signal, consistent with the annotation style used in the main figures. The images already show two representative cells per condition to maximise the visible detail at the chosen scale.

9. SF9G: G is labelled, but not included.
Response: Figure S9G has been added in the revised manuscript, showing the pan-cancer overall survival map for GOLGB1 expression across all TCGA cohorts generated using GEPIA2. The figure legend has been updated accordingly.

Reviewer #3 (Significance (Required)):

The work finds new roles for the Golgi in regulation of DNA damage responses and the screen could be an important dataset (but results need to be made available) for the DNA repair community. The scope of the initial screen of HPA antibodies and Golgi/Nuclear dual proteomes is impressive, and the overlap of DDR proteins is characterized for fifteen different proteins at a sub-compartmental level. The work provides important insights into RAD51C regulation, however, there are key mechanistic insights and control experiments missing from the studies involving RAD51C and Giantin, dampening its impact. The idea of an alternative cellular compartment for storage of DDR factors prior to damage is interesting, and suggests the spatial regulation of specific lesion responses are stored in specific sub-compartments of the Golgi, which could contribute to repair regulation.

References:

Adamson B, Smogorzewska A, Sigoillot FD, King RW & Elledge SJ (2012) A genome-wide homologous recombination screen identifies the RNA-binding protein RBMX as a component of the DNA-damage response. *Nat Cell Biol* 14: 318–328

Badie S, Liao C, Thanasoula M, Barber P, Hill MA & Tarsounas M (2009) RAD51C facilitates checkpoint signaling by promoting CHK2 phosphorylation. *J Cell Biol* 185: 587–600

Bergen DJM, Stevenson NL, Skinner REH, Stephens DJ & Hammond CL (2017) The Golgi matrix protein giantin is required for normal cilia function in zebrafish. *Biol Open* 6: 1180–1189

Berti M, Teloni F, Mijic S, Ursich S, Fuchs J, Palumbieri MD, Krietsch J, Schmid JA, Garcin EB, Gon S, *et al* (2020) Sequential role of RAD51 paralog complexes in replication fork remodeling and restart. *Nat Commun* 11: 3531

Boutros M, Kiger AA, Armknecht S, Kerr K, Hild M, Koch B, Haas SA, Paro R, Perrimon N & Heidelberg Fly Array Consortium (2004) Genome-wide RNAi analysis of growth and viability in Drosophila cells. *Science* 303: 832–835

Brinkmann K, Schell M, Hoppe T & Kashkar H (2015) Regulation of the DNA damage response by ubiquitin conjugation. *Front Genet* 6: 98

Garvey CM, Spiller E, Lindsay D, Chiang C-T, Choi NC, Agus DB, Mallick P, Foo J & Mumenthaler SM (2016) A high-content image-based method for quantitatively studying context-dependent cell population dynamics. *Sci Rep* 6: 29752

Ghannoum S, Fantini D, Zahoor M, Reiterer V, Phuyal S, Leoncio Netto W, Sørensen Ø, Iyer A, Sengupta D, Prasmickaite L, *et al* (2023) A combined experimental-computational approach uncovers a role for the Golgi matrix protein Giantin in breast cancer progression. *PLoS Comput Biol* 19: e1010995

Greenhough LA, Liang C-C, Belan O, Kunzelmann S, Maslen S, Rodrigo-Brenni MC, Anand R, Skehel M, Boulton SJ & West SC (2023) Structure and function of the RAD51B–RAD51C–RAD51D–XRCC2 tumour suppressor. *Nature* 619: 650–657

Halim VA, García-Santisteban I, Warmerdam DO, van den Broek B, Heck AJR, Mohammed S & Medema RH (2018) Doxorubicin-induced DNA damage causes extensive ubiquitination of ribosomal proteins associated with a decrease in protein translation. *Mol Cell Proteomics* 17: 2297–2308

Koreishi M, Gniadek TJ, Yu S, Masuda J, Honjo Y & Satoh A (2013) The golgin tether giantin regulates the secretory pathway by controlling stack organization within Golgi apparatus. *PLoS One* 8: e59821

Martin HL, Adams M, Higgins J, Bond J, Morrison EE, Bell SM, Warriner S, Nelson A & Tomlinson DC (2014) High-content, high-throughput screening for the identification of cytotoxic compounds based on cell morphology and cell proliferation markers. *PLoS One* 9: e88338

Meindl A, Hellebrand H, Wiek C, Erven V, Wappenschmidt B, Niederacher D, Freund M, Lichtner P, Hartmann L, Schaal H, *et al* (2010) Germline mutations in breast and ovarian cancer pedigrees establish RAD51C as a human cancer susceptibility gene. *Nat Genet* 42: 410–414

Mikheeva AM, Bogomolov MA, Gasca VA, Sementsov MV, Spirin PV, Prassolov VS & Lebedev TD (2024) Improving the power of drug toxicity measurements by quantitative nuclei imaging. *Cell Death Discov* 10: 181

Ovejero S, Kumanski S, Soulet C, Azarli J, Pardo B, Santt O, Constantinou A, Pasero P & Moriel-Carretero M (2023) A sterol-PI(4)P exchanger modulates the Tel1/ATM axis of the DNA damage response. *EMBO J* 42: e112684

Prakash R, Rawal Y, Sullivan MR, Grundy MK, Bret H, Mihalevic MJ, Rein HL, Baird JM, Darrah K, Zhang F, *et al* (2022) Homologous recombination-deficient mutation cluster in tumor suppressor RAD51C identified by comprehensive analysis of cancer variants. *Proc Natl Acad Sci U S A* 119: e2202727119

Prakash R, Zhang Y, Feng W & Jasin M (2015) Homologous recombination and human health: the roles of BRCA1, BRCA2, and associated proteins. *Cold Spring Harb Perspect Biol* 7: a016600

Presley JF, Cole NB, Schroer TA, Hirschberg K, Zaal KJM & Lippincott-Schwartz J (1997) ER-to-Golgi transport visualized in living cells. *Nature* 389: 81–85

Rawal Y, Jia L, Meir A, Zhou S, Kaur H, Ruben EA, Kwon Y, Bernstein KA, Jasin M, Taylor AB, *et al* (2023) Structural insights into BCDX2 complex function in homologous recombination. *Nature* 619: 640–649

Somyajit K, Saxena S, Babu S, Mishra A & Nagaraju G (2015) Mammalian RAD51 paralogs protect nascent DNA at stalled forks and mediate replication restart. *Nucleic Acids Res* 43: 9835–9855

Soulet C, Catalan J & Moriel-Carretero M (2026) The DNA Damage Response kinase ATM restricts Golgi extension. *bioRxiv*

Stadler C, Hjelmare M, Neumann B, Jonasson K, Pepperkok R, Uhlén M & Lundberg E (2012) Systematic validation of antibody binding and protein subcellular localization using siRNA and confocal microscopy. *J Proteomics* 75: 2236–2251

Stevenson NL, Bergen DJM, Lu Y, Prada-Sanchez ME, Kadler KE, Hammond CL & Stephens DJ (2021) Correction: Giantin is required for intracellular N-terminal processing of type I procollagen. *J Cell Biol* 220

Tang Z, Kang B, Li C, Chen T & Zhang Z (2019) GEPIA2: an enhanced web server for large-scale expression profiling and interactive analysis. *Nucleic Acids Res* 47: W556–W560

Thorn CF, Oshiro C, Marsh S, Hernandez-Boussard T, McLeod H, Klein TE & Altman RB (2011) Doxorubicin pathways: pharmacodynamics and adverse effects. *Pharmacogenet Genomics* 21: 440–446

van der Zanden SY, Qiao X & Neefjes J (2021) New insights into the activities and toxicities of the old anticancer drug doxorubicin. *FEBS J* 288: 6095–6111

Zhang Y-W, Otterness DM, Chiang GG, Xie W, Liu Y-C, Mercurio F & Abraham RT (2005) Genotoxic stress targets human Chk1 for degradation by the ubiquitin-proteasome pathway. *Mol Cell* 19: 607–618

May 27, 2026

RE: JCB Manuscript #202605024T

George Galea
European Molecular Biology Laboratory

Dear Dr. Galea:

Thank you for submitting your revised manuscript entitled "Spatiotemporal dynamics of DNA repair proteins between the Golgi and nucleus maintain genomic stability". Your study has been reassessed by two of the original reviewers from Review Commons who are now supportive of publication. Therefore, we would be happy to publish your paper in JCB pending final revisions necessary to meet our formatting guidelines (see details below).

To avoid unnecessary delays in the acceptance and publication of your paper, please read the following information carefully.

A. MANUSCRIPT ORGANIZATION AND FORMATTING:

Full guidelines are available on our Instructions for Authors page, https://jcb.rupress.org/submission-guidelines#revised.
Submission of a paper that does not conform to JCB guidelines will delay the acceptance of your manuscript.

1) Text limits: Character count for Articles is < 40,000, not including spaces. Count includes abstract, introduction, results, discussion, and acknowledgments. Count does not include title page, figure legends, materials and methods, references, tables, or supplemental legends.

2) Figures limits: Articles may have up to 10 main text figures.

3) Figure formatting: Scale bars must be present on all microscopy images, including inset magnifications. Molecular weight or nucleic acid size markers must be included on all gel electrophoresis. Aspect ratios of images may not be altered. To accommodate readers with red-green color blindness, we suggest that you change all red-green images.

4) Statistical analysis: Error bars on graphic representations of numerical data must be clearly described in the figure legend. The number of independent data points (n) represented in a graph must be indicated in the legend. Statistical methods should be explained in full in the materials and methods. For figures presenting pooled data the statistical measure should be defined in the figure legends. Please also be sure to indicate the statistical tests used in each of your experiments (either in the figure legend itself or in a separate methods section) as well as the parameters of the test (for example, if you ran a t-test, please indicate if it was one- or two-sided, etc.). Also, if you used parametric tests, please indicate if the data distribution was tested for normality (and if so, how). If not, you must state something to the effect that "Data distribution was assumed to be normal but this was not formally tested."

5) Abstract and title: The abstract should be no longer than 160 words and should communicate the significance of the paper for a general audience. The title should be less than 100 characters including spaces. Make the title concise but accessible to a general readership.

6) Materials and methods: Should be comprehensive and not simply reference a previous publication for details on how an experiment was performed. Please provide full descriptions in the text for readers who may not have access to referenced manuscripts.

7) All antibodies, cell lines, animals, and tools used in the manuscript should be described in full, including accession numbers for materials available in a public repository such as the Resource Identification Portal. Please be sure to provide the sequences for all of your primers/oligos and RNAi constructs in the materials and methods. You must also indicate in the methods the source, species, and catalog numbers (where appropriate) for all of your antibodies. Please also indicate the acquisition and quantification methods for immunoblotting/western blots.

8) Microscope image acquisition: The following information must be provided about the acquisition and processing of images:
a. Make and model of microscope
b. Type, magnification, and numerical aperture of the objective lenses
c. Temperature
d. Imaging medium
e. Fluorochromes
f. Camera make and model
g. Acquisition software

h. Any software used for image processing subsequent to data acquisition. Please include details and types of operations involved (e.g., type of deconvolution, 3D reconstitutions, surface or volume rendering, gamma adjustments, etc.).

9) References: There is no limit to the number of references cited in a manuscript. References should be cited parenthetically in the text by author and year of publication. Abbreviate the names of journals according to PubMed.

10) Supplemental materials: There are strict limits on the allowable amount of supplemental data. Articles may have up to 5 supplemental figures. Please reduce your SI count, for example you can move some images into the main figures, and be sure to correct the callouts in the text to reflect all changes. Please also note that tables, like figures, should be provided as individual, editable files. A summary of all supplemental material should appear at the end of the Materials and methods section.

11) eTOC summary: A ~40-50-word summary that describes the context and significance of the findings for a general readership should be included on the title page. The statement should be written in the present tense and refer to the work in the third person.

12) Conflict of interest statement: JCB requires inclusion of a statement in the acknowledgements regarding competing financial interests. If no competing financial interests exist, please include the following statement: "The authors declare no competing financial interests." If competing interests are declared, please follow your statement of these competing interests with the following statement: "The authors declare no further competing financial interests."

13) ORCID IDs: ORCID IDs are unique identifiers allowing researchers to create a record of their various scholarly contributions in a single place. Please note that ORCID IDs are now *required* for all authors. At resubmission of your final files, please be sure to provide your ORCID ID and those of all co-authors.

14) A separate author contribution section following the Acknowledgments. All authors should be mentioned and designated by their full names. We encourage use of the CRediT nomenclature.

Please note that JCB now requires authors to submit Source Data used to generate figures containing gels and Western blots with all revised manuscripts. This Source Data consists of fully uncropped and unprocessed images for each gel/blot displayed in the main and supplemental figures. For assays performed using capillary electrophoresis and/or immunoassay-based detection, authors should instead provide the electropherogram graph(s) for each experiment, plotting fluorescence/chemiluminescence intensity vs. molecular weight/size. Please be sure to provide one Source Data file for each figure gels, blots, and/or capillary electrophoresis assays along with your revised manuscript files. File names for Source Data figures should be alphanumeric without any spaces or special characters (i.e., SourceDataF#, where F# refers to the associated main figure number or SourceDataFS# for those associated with Supplementary figures). For traditional gels and blots, the lanes of the gels/blots should be labeled as they are in the associated figure, the place where cropping was applied should be marked (with a box), and molecular weight/size standards should be labeled wherever possible. For capillary electrophoresis assays, each trace in the graph should be color-coded and labeled to indicate which protein, gene, or sample is being measured (please try to avoid red/green combinations to accommodate our color-blind readers).

Source Data files will be made available to reviewers during evaluation of revised manuscripts and, if your paper is eventually published in JCB, the files will be directly linked to specific figures in the published article.

Source Data Figures should be provided as individual PDF files (one file per figure). Authors should endeavor to retain a minimum resolution of 300 dpi or pixels per inch. Please review our instructions for export from Photoshop, Illustrator, and PowerPoint here: https://rupress.org/jcb/pages/submission-guidelines#revised

Journal of Cell Biology now requires a data availability statement for all research article submissions. These statements will be published in the article directly above the Acknowledgments. The statement should address all data underlying the research presented in the manuscript. Please visit the JCB instructions for authors for guidelines and examples of statements at (https://rupress.org/jcb/pages/editorial-policies#data-availability-statement).

B. FINAL FILES:

Please upload the following materials to our online submission system. These items are required prior to acceptance. If you have any questions, contact JCB's Managing Editor, Lindsey Hollander (lhollander@rockefeller.edu).

-- An editable version of the final text (.DOC or .DOCX) is needed for copyediting (no PDFs).

-- High-resolution figure and MP4 video files: See our detailed guidelines for preparing your production-ready images, https://jcb.rupress.org/fig-vid-guidelines.

-- Cover images: If you have any striking images related to this story, we would be happy to consider them for inclusion on the journal cover. Submitted images may also be chosen for highlighting on the journal table of contents or JCB homepage carousel.

Images should be uploaded as TIFF or EPS files and must be at least 300 dpi resolution.

It is JCB policy that if requested, original data images must be made available to the editors. Failure to provide original images upon request will result in unavoidable delays in publication. Please ensure that you have access to all original data images prior to final submission.

The license to publish form must be signed before your manuscript can be sent to production. A link to the license to publish form will be sent to the corresponding author only. Please take a moment to check your funder requirements before choosing the appropriate license.

Additionally, JCB encourages authors to submit a short video summary of their work. These videos are intended to convey the main messages of the study to a non-specialist, scientific audience. Think of them as an extended version of your abstract, or a short poster presentation. We encourage first authors to present the results to increase their visibility. The videos will be shared on social media to promote your work. For more detailed guidelines and tips on preparing your video, please visit https://rupress.org/jcb/pages/submission-guidelines#videoSummaries.

Thank you for your attention to these final processing requirements. Please revise and format the manuscript and upload materials within 14 days. If you need an extension for whatever reason, please let us know and we can work with you to determine a suitable revision period.

Please contact the journal office with any questions at cellbio@rockefeller.edu.

Thank you for this interesting contribution, we look forward to publishing your paper in Journal of Cell Biology.

Sincerely,

Roger Greenberg
Monitoring Editor

Andrea L. Marat
Deputy Editor

Journal of Cell Biology

Reviewer #1 (Comments to the Authors (Required)):

The authors have addressed the concerns I previously raised in the Review Commons review and I am happy to recommend publication in JCB.

Reviewer #3 (Comments to the Authors (Required)):

In this work, the authors have employed siRNA knockdown screening approach to investigate functional linkages between the Golgi complex and the nucleus. From their screen, the authors find a novel cluster of DNA repair proteins present in the Golgi. Focusing on RAD51C, they show it is present in significant amounts in the Golgi where it is regulated by Giantin. The possibility of the Golgi storing specific DDR factors in specific compartments prior to damage is well-supported and compelling.
Overall, in this resubmission, the authors have put in a great deal of effort in addressing earlier concerns made following the initial Reviewer Commons submission. They have made all the requested edits and have largely sufficiently justified the minor comments that they did not change. In addition, the figures are much easier to see and understand, and the in-text discussion reflects their data. The data is much better presented, the screen and the available supplemental table 1 clarified their workflow. Statistical analyses have also been improved, with added use of ANOVA where requested. Overall, based on the improvements made to my and the other reviewers' earlier comments, I recommend publication in JCB.

Response to reviewers: Spatiotemporal regulation of DNA repair proteins between Golgi and nucleus ensures genome stability

Response to reviewers.

We are grateful to the editors and reviewers for their constructive and thorough engagement with our manuscript across both rounds of reviews. We are delighted that both reviewers recommend publication.

Reviewer #1 (Comments to the Authors (Required))

The authors have addressed the concerns I previously raised in the Review Commons review and I am happy to recommend publication in JCB.

Reviewer #3 (Comments to the Authors (Required)):

In this work, the authors have employed siRNA knockdown screening approach to investigate functional linkages between the Golgi complex and the nucleus. From their screen, the authors find a novel cluster of DNA repair proteins present in the Golgi. Focusing on RAD51C, they show it is present in significant amounts in the Golgi where it is regulated by Giantin. The possibility of the Golgi storing specific DDR factors in specific compartments prior to damage is well-supported and compelling.

Overall, in this resubmission, the authors have put in a great deal of effort in addressing earlier concerns made following the initial Reviewer Commons submission. They have made all the requested edits and have largely sufficiently justified the minor comments that they did not change. In addition, the figures are much easier to see and understand, and the in-text discussion reflects their data. The data is much better presented, the screen and the available supplemental table 1 clarified their workflow. Statistical analyses have also been improved, with added use of ANOVA where requested. Overall, based on the improvements made to my and the other reviewers' earlier comments, I recommend publication in JCB.
